# Adventitial SCA-1^+^ Progenitor Cell Gene Sequencing Reveals the Mechanisms of Cell Migration in Response to Hyperlipidemia

**DOI:** 10.1016/j.stemcr.2017.06.011

**Published:** 2017-07-27

**Authors:** Ioannis Kokkinopoulos, Mei Mei Wong, Claire M.F. Potter, Yao Xie, Baoqi Yu, Derek T. Warren, Witold N. Nowak, Alexandra Le Bras, Zhichao Ni, Chao Zhou, Xiongzhong Ruan, Eirini Karamariti, Yanhua Hu, Li Zhang, Qingbo Xu

**Affiliations:** 1 Cardiovascular Division, King's College London BHF Centre, 125 Coldharbour Lane, London SE5 9NU, UK; 2 John Moorhead Research Laboratory, Centre for Nephrology, University College London, Rowland Hill Street, London NW3 2PF, UK; 3 Department of Cardiology, The First Affiliated Hospital, Zhejiang University, 79 Qingchun Road, Hangzhou 310003, China

**Keywords:** vascular progenitors, adventitial migration, hyperlipidemia, atherosclerosis, extracellular matrix

## Abstract

Adventitial progenitor cells, including SCA-1^+^ and mesenchymal stem cells, are believed to be important in vascular remodeling. It has been shown that SCA-1^+^ progenitor cells are involved in neointimal hyperplasia of vein grafts, but little is known concerning their involvement in hyperlipidemia-induced atherosclerosis. We employed single-cell sequencing technology on primary adventitial mouse SCA-1^+^ cells from wild-type and atherosclerotic-prone (ApoE-deficient) mice and found that a group of genes controlling cell migration and matrix protein degradation was highly altered. Adventitial progenitors from ApoE-deficient mice displayed an augmented migratory potential both *in vitro* and *in vivo*. This increased migratory ability was mimicked by lipid loading to SCA-1^+^ cells. Furthermore, we show that lipid loading increased *miRNA-29b* expression and induced sirtuin-1 and matrix metalloproteinase-9 levels to promote cell migration. These results provide direct evidence that blood cholesterol levels influence vascular progenitor cell function, which could be a potential target cell for treatment of vascular disease.

## Highlights

• Hyperlipidemia affects migration of AdvSca-1^+^ cells
• AdvSca-1^+^ ApoE KO cells show altered matrix of proteins
• Decorin ameliorates extensive migration of AdvSca-1^+^ cells toward the neointima
• *MicroRNA-29* interferes with AdvSca-1^+^ migration via SIRT-1 and MMP-9

## Teaser

In this article, Xu and colleagues show that hyperlipidemia may confer to neointimal formation by extensive mobilization of adventitial progenitors. Using the ApoE KO murine model, they show that increased cholesterol levels augment adventitia progenitor migration, both *in vitro* and *in vivo*, through a modified matrix of proteins. Migratory capability could be ameliorated through mechanisms involving collagen formation and *microRNA* inhibition.

## Introduction

Atherosclerosis is a condition whereby the arteries supplying target organs, such as the brain and the heart, become occluded. It is a disease of chronic inflammation and the leading cause of heart ischemia (Lusis, 2000). It is characterized by subendothelial retention of modified lipoproteins, causing local inflammation and neointimal lesion formation (Williams and Tabas, 1995). This local effect exacerbates the activation and consequent loss of the endothelial cell (EC) layer and profound vascular smooth muscle cell (vSMC) proliferation, as well as recruitment of monocytes and activation of resident macrophages. Consequently, all three layers that comprise the vascular cell wall (adventitia, media, and EC) undergo remodeling with an accumulation of cellular and extracellular matrix (ECM) material. Over the last decade, vascular progenitors have been identified in the vessel wall in humans and rodents (Alessandri et al., 2001, Ferreira et al., 2007, Hill et al., 2010, Hu et al., 2004, Invernici et al., 2008, Passman et al., 2008, Psaltis et al., 2014, Sirker et al., 2009, Tang et al., 2012, Tavian et al., 2005, Tilki et al., 2009, Wu et al., 2008, Xu et al., 2003, Zengin et al., 2006). The murine adventitia contains a heterogeneous population of progenitor/precursor cells with the majority of them expressing stem cell antigen 1 (SCA-1). In atherosclerotic arteries, the adventitial layer displays increased cell proliferation and inflammation (for review see Seidelmann et al., 2014). Recent studies have highlighted the potential role of the adventitia in the development of neointimal lesion of vessel grafts (Chen et al., 2013, Wong et al., 2013). However, less is known about the involvement of vascular stem/progenitor cells in native atherosclerosis, the leading cause of cardiovascular death in the general population.

Resident vascular progenitors possess a bilineage potential, able to differentiate into vSMCs and ECs, both *in vitro* and *in vivo* (Hu et al., 2004, Torsney et al., 2005, Wong et al., 2013). Utilization of an SM22-LacZ mouse model showed that SCA-1^+^β-gal^+^ cells could be traced to neointimal lesions, confirming their differentiation toward an SMC-like phenotype. When adventitial SCA-1^+^ (AdvSCA-1^+^) cells were applied to the external side of vein grafts, prior to isografting to ApoE knockout (KO) and wild-type (WT) mice, they were shown to contribute to lesions via unknown mechanisms.

These murine SCA-1^+^ cells have been identified as the largest vascular progenitor cell population to date, with subpopulations expressing C-KIT and CD34 (Hu et al., 2004). Bone marrow-derived circulating cells may serve as a second potential source of vascular progenitors. In support of this, SCA-1^+^ cells also express the macrophage/monocyte marker CD45 (Psaltis et al., 2014). Noseda et al. (2015) reported that there are at least two distinct types of SCA-1^+^ cells in the adult mouse heart, with one segregating to a cardiogenic potential and the other toward a vascular one. Previously, we demonstrated that SCA-1^+^-derived vSMCs present in neointimal lesions originated from non-bone marrow-derived resident vascular progenitors (Hu et al., 2002a, Hu et al., 2002b, Xu et al., 2003). Although adventitial progenitors have been proposed to contribute to vascular disease (Campagnolo et al., 2015, Chen et al., 2013, Hu et al., 2004, Passman et al., 2008, Psaltis et al., 2014, Tigges et al., 2013), quantitative data on the contribution of local resident versus bone marrow-derived progenitors is still lacking.

For clarifying the role of AdvSCA-1^+^ progenitors in native atherosclerosis, it is essential to elucidate their differential gene expression profile between atherosclerosis-resistant and atherosclerosis-susceptible mice. In identifying which pathways are altered during naturally occurring atherosclerosis, we may better understand their potential contribution to neointimal lesion development and maintenance. Here, we employed single-cell gene expression analysis of AdvSCA-1^+^ cells isolated from the adult aorta of WT and ApoE KO mice. Our sequencing analysis revealed that ApoE KO SCA-1^+^ cells have an altered gene expression profile for cytoskeletal rearrangements compared with WT SCA-1^+^ cells, making them more receptive to extrinsic migratory cues. Subsequent mechanistic analysis with a focus on ApoE KO SCA-1^+^ progenitors identified a potential cell-autonomous mechanism involving *microRNA* in ameliorating adventitial progenitor migration that could be of use in a clinical setting.

## Results

### A Gene Signature of a Heightened Migratory Capability in ApoE KO AdvSCA-1^+^ Cells

**Figure 1:**
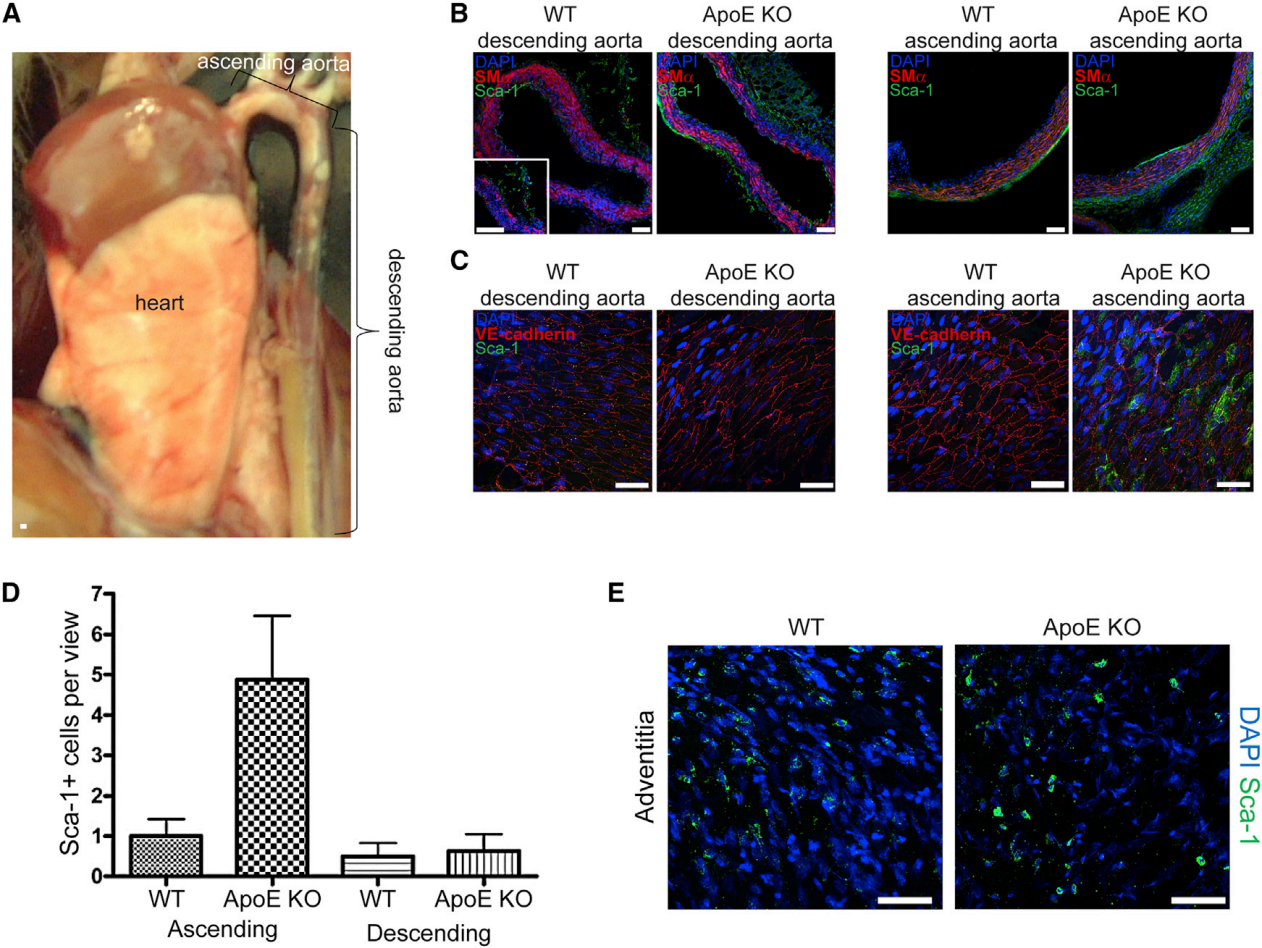
Adventitial ApoE KO SCA-1^+^ Cell Distribution Differs from That of WT (A) Microphotograph of a 6-month-old mouse WT abdominal aorta and heart. (B) Immunohistochemical labeling of WT and ApoE KO thoracic and root aortas for SCA-1 (green) and smooth muscle cell actin (SMα, red). Inset: magnified photo of WT adventitia (n = 3 mouse aortas). (C) *En face* immunohistochemical labeling of WT and ApoE KO ascending and descending EC layer for SCA-1 (green) and endothelial cell marker (VE-cadherin) (n = 4 mouse aortas). (D) Bar graph showing the number of SCA-1^+^ cells in both WT and ApoE KO EC layers of ascending and descending aortas (n = 14 independent experiments). Student's t test, p < 0.01. (E) *En face* immunohistochemical labeling of the adventitia of the descending aorta of WT and ApoE KO 6-month-old mouse SCA-1 (green), with notably more cells labeled in the ApoE KO adventitia (n = 3 mouse aortas). Scale bars, 10 μm.

**Figure 2:**
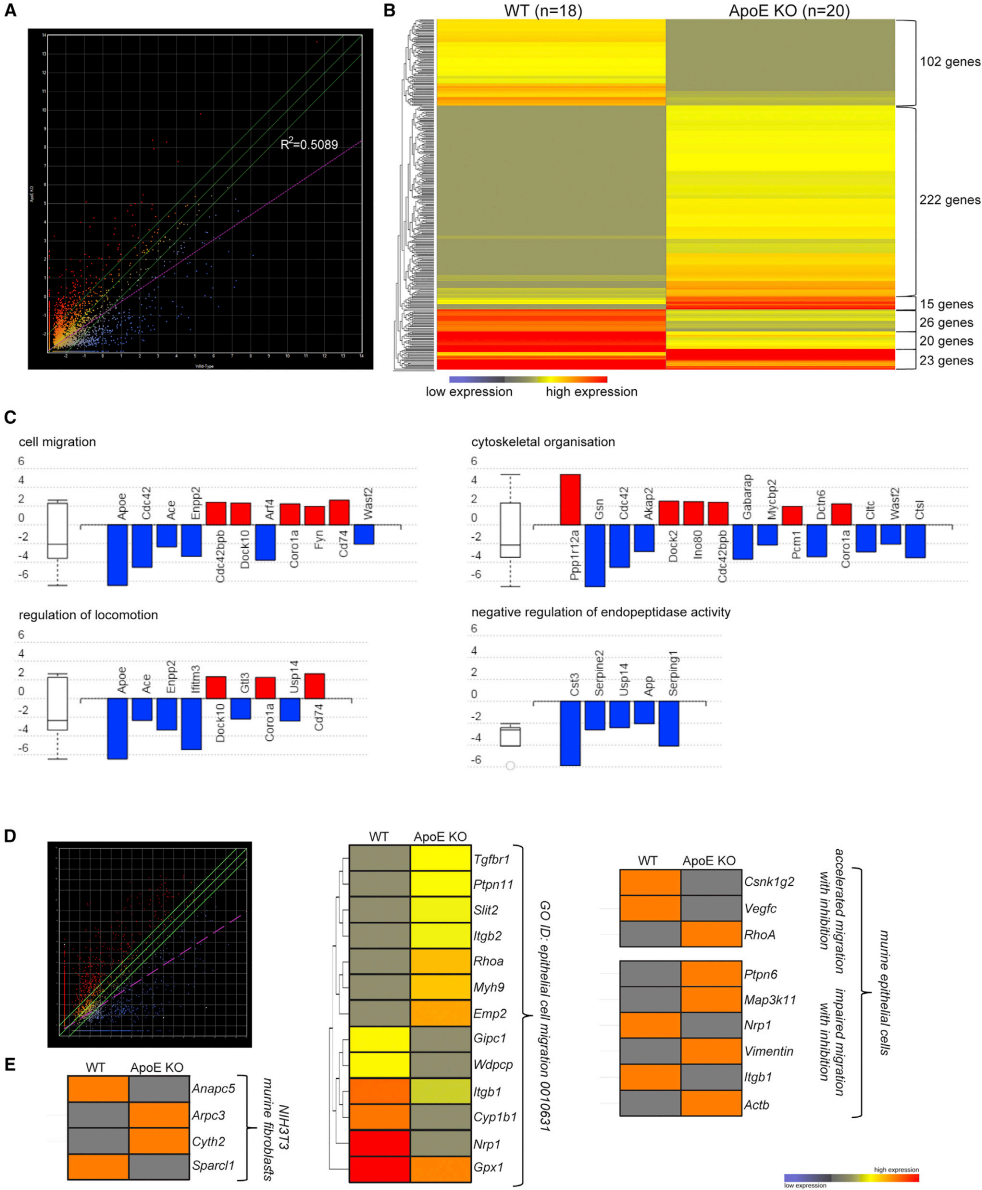
Differential Gene Expression Profile between WT and ApoE KO Adventitial SCA-1^+^ Progenitors (A) Scattergraph representing the relative single-cell expression of the 26,596 genes analyzed between WT (n = 18, y axis) and ApoE KO (n = 20, x axis) AdvSCA-1^+^ cells (type II error rate 0.2, r^2^ = 0.52). (B) Heatmap representation of six major gene clusters showing the median statistically significant difference in relative gene expression between WT and ApoE KO AdvSCA-1^+^ cells (Student's t test, p < 0.01, with a bidirectional 2 SD cutoff). (C) GO biological processes revealed that key genes involved in regulation of cell migration, locomotion, cytoskeletal organization, and endopeptidase activity were downregulated 4-fold in the ApoE KO AdvSCA-1^+^ cell population, in comparison with the WT (Erim pruning, >5 differentially expressed genes). (D) Scattergraph representing the relative gene expression of AdvSCA-1^+^ cells isolated, with high copy number of the *Sca-1* gene, analyzed between WT (n = 4, y axis) and ApoE KO (n = 5, x axis). (E) Heatmap representation of median gene expression involved in NIH3T3 murine fibroblast cell and epithelial cell migration (adapted from Simpson et al., 2008) shows a difference in relative expression between WT and ApoE KO AdvSCA-1^+^ cells (>4-fold gene expression difference, Student's t test, p < 0.01, with a bidirectional 2 SD cutoff).

WT mice do not develop atherosclerosis (Figure S1A). The ApoE KO mice develop neointimal lesions and endothelial layer lipid residues, along with an expanded adventitial layer from 6 months onward (Figure S1B). The adventitia and intima layers have been shown to contain SCA-1^+^ cells (Hu et al., 2004). Immunolabeling of the descending aortas and the root of WT (Figure 1A) and ApoE KO aortas revealed an increase of SCA-1^+^ signal in both the adventitial and intimal layers of the mutant vascular wall (Figure 1B), as well as *in vitro* (Figure S2C). We confirmed this increase by *en face* immunolabeling of both descending and ascending aortas (Figures 1C–1E). To address the innate heterogeneity of the AdvSCA-1^+^ cell population, we collected unpassaged adventitial cells from both WT and ApoE KO mice, obviating potential bias from *in vitro* expansion. These cells were then enriched for SCA-1 surface expression prior to single-cell expression analysis. A total of 25,596 genes were analyzed between the two SCA-1^+^ cell populations (Figure 2A) and statistical analysis revealed 408 clustered genes that were significantly differentially expressed (Figure 2B). Gene Ontology (GO) pathway enrichment analysis revealed four predominant pathways being altered: cell migration, cytoskeletal organization, regulation of locomotion, and endopeptidase activity (Figure 2C). Enrichment for cellular components indicated several pathways being affected, especially concerning ECM organization and maintenance, including the exosome pathway (Figure S1C).

Potential epithelial migration, namely that of AdvSCA-1^+^ progenitor cell migration toward the inner vascular wall, depends on the activity and gene expression of molecules able to disrupt the ECM, as well as promoting cytoskeletal rearrangements, including cell polarity. Our single-cell expression analysis revealed that matrix metallopeptidases, integrins, and collagen gene expression differed between ApoE KO and WT while AdvSCA-1^+^ cells in the mutant setting suggested a heightened epithelial-to-mesenchymal transition (EMT) (Figure S1D). Of note, 5% of WT AdvSCA-1^+^ cells expressed bone marrow hematopoietic progenitor genes, compared with 20% of the ApoE KO cells, from which a subpopulation of 10% was also positive for *Cd45*, 20% positive for C*d34*, and 5% positive for *Thy1* genes, as reported previously (Figure S1E; for review see Bobryshev et al., 2015).

### ApoE KO AdvSCA-1^+^ Enhanced Migration Is Intrinsic

**Figure 3:**
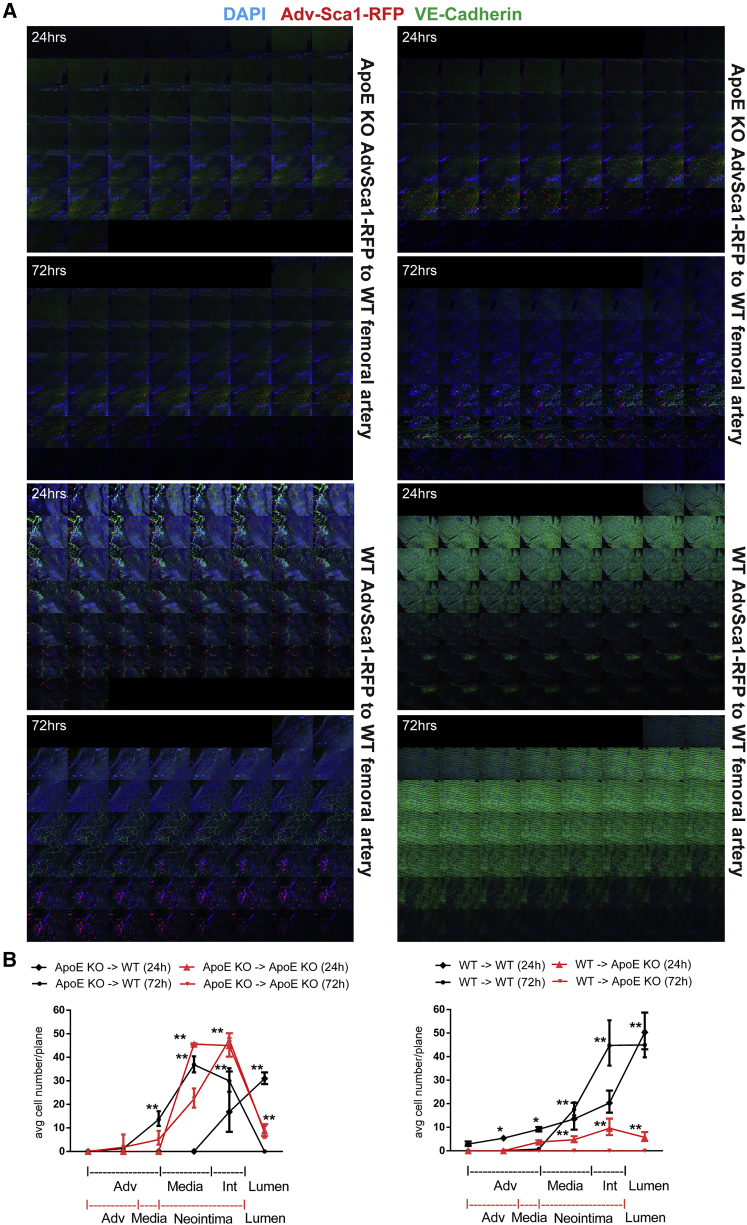
ApoE KO SCA-1^+^ Cells Show a Higher Migration toward the Inner Vascular Wall in Comparison with WT Counterparts (A) Montage microphotographs. WT and ApoE KO AdvSCA-1^+^ cells were transduced with a nuclear RFP-inducing lentiviral construct. 1 × 10^6^ cells in Matrigel plugs were transplanted to the outer side of the vascular wall of femoral arteries, in WT and ApoE KO 6-month-old animals. Cells were allowed to migrate for 24 and 72 hr prior to euthanizing the mice and assessing them with *en face* immunolabeling, using a VE-cadherin (green) antibody to mark the endothelial cell layer. DAPI is blue. (B) Quantitative and spatial analysis of RFP^+^ cells migrating from the adventitia to the lumen from the outer vascular wall after 24 and 72 hr. n = 3 transplantation experiments, geometric means, error bars with 95% CI. ^∗^p < 0.05, ^∗∗^p < 0.01.

To address any mechanisms that allow mutant AdvSCA-1^+^ cells to migrate more prominently, we first enriched our single-cell expression analysis for those AdvSCA-1^+^ cells with a high copy of *Sca-1* (Figure 2D), since in our established *in vitro* system setting passaged cells are used, which are highly enriched for SCA-1 through multiple magnetic bead cell sorting (Mayr et al., 2008, Yu et al., 2016). Our findings were analyzed using genes known to correlate with epithelial mouse cell expression (Seo et al., 2010, Simpson et al., 2008) (Figure 2E). The ApoE KO's epithelial migration gene expression plexus was diverted in comparison with WT. To confirm this, we transduced WT and ApoE KO AdvSCA-1^+^ with an RFP lentiviral construct to permanently label the cells and their progenies, if any. WT and ApoE KO AdvSCA-1^+^-RFP cells were transplanted into the outer wall of the femoral artery of adult WT and ApoE KO mice in all four combinations (Figure 3A). Mice were then euthanized 24 or 72 hr later. In comparison with the WT, ApoE KO AdvSCA-1^+^ displayed a greater tendency to migrate into the ApoE KO femoral artery. WT AdvSCA-1^+^ cells migrated poorly into the ApoE KO femoral artery (Figure 3B).

**Figure 4:**
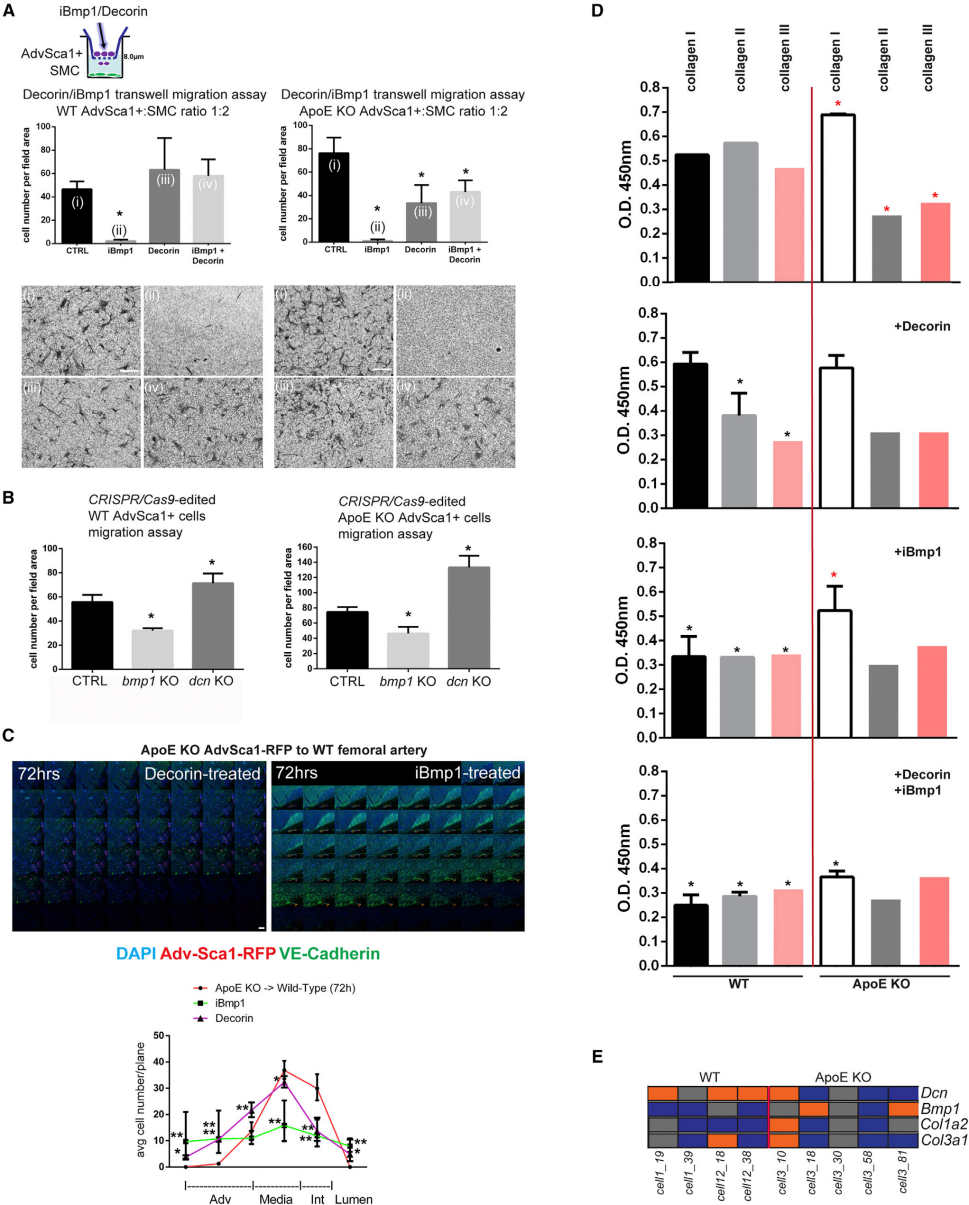
Intervention Targets for Adventitial ApoE KO SCA-1^+^ Inhibition of Cell Migration toward the Media (A) 1 × 10^5^ WT or ApoE KO AdvSCA-1^+^ cells were loaded with 10 μg/mL iBMP1 and/or DCN in 0.2% fetal bovine serum (FBS) and allowed to pass through 8.0-μm transwells seeded with 1 × 10^5^ WT vSMCs for 24 hr. n = 3 independent experiments, geometric means, error bars with 95% CI. ^∗^p < 0.05. Scale bars, 50 μm. (B) CRISPR/Cas9-edited AdvSCA-1^+^ cells were induced to migrate in 0.2% FBS passing through 8.0-μm transwells for 24 hr. n = 3 independent experiments. Error bars are SEM. ^∗^p = 0.05 compared with control. (C) Montage microphotographs. WT and ApoE KO AdvSCA-1^+^ cells were transduced with a nuclear RFP-inducing lentiviral construct. Matrigel plugs containing 1 × 10^6^ cells were transplanted to the outer side of the vascular wall, in WT and ApoE KO 6-month-old animals (n = 3 transplantation experiments). Cells were allowed to migrate for 72 hr prior to euthanizing the mice and assessing them with *en face* immunolabeling, using a VE-cadherin antibody to mark the EC layer. DAPI is blue. Quantitative and spatial analysis of RFP^+^ cells migrating from the adventitia to the lumen from the outer vascular wall. ^∗^p < 0.05 and ^∗∗^p < 0.01 compared with the untreated group. (D) 3 × 10^4^ WT or ApoE KO AdvSCA-1^+^ cells were seeded onto 12-well plates and treated with iBMP1 and/or DCN. Supernatant was collected after 24 hr. ELISA assays were performed against pro-collagens I and II, and collagen III. n = 3 independent experiments. ^∗^p < 0.05 compared with untreated control. ^∗^p < 0.05, WT compared with ApoE KO. (E) Heatmap of *Sca-1*^high^ cells differentially expressing *Dcn*, *Bmp1*, *Col1a2*, and *Col3a1*.

We assessed whether specific genes are differentially expressed between the ApoE-derived AdvSCA-1^+^ cells compared with their WT counterparts (Figures S2A and S2B). Thirteen genes identified were known to have an effect on migration; six of them were also important for EC lumen formation, and five were important for leukocyte homing. Of those, *Cystatin c*, *Bone morphogenic protein 1* (*Bmp1*), and *Decorin* (*Dcn*) were selected for further investigation of AdvSCA-1^+^ cell migration. Since *Bmp1* expression was elevated, we assessed whether soluble Decorin (DCN) and a BMP1-specific inhibitor (C_15_H_24_N_4_O_4_, iBMP1) would be able to modulate cell migration in a wound-healing assay (von Marschall and Fisher, 2010) (Figure S2D). DCN was detected in the adventitia of WT aortas (Al Haj Zen et al., 2006), confirming the single-cell data outcome (Figure S2E). In contrast, DCN expression was observed in the medial layer of the ApoE KO in SMCs. This was confirmed by ELISA on WT and ApoE KO AdvSCA-1^+^ as well as in SMCs, *in vitro*, in concert with our single-cell expression profiling. DCN and iBMP1 both decreased the migration of ApoE KO AdvSCA-1^+^ cells when administered individually and in combination. We then tested the effect of these substances in transwell migration assays (Figure 4A). Cystatin C had no effect on migration (data not shown), while iBMP1 affected both mutant and WT AdvSCA-1^+^ cells. DCN influenced ApoE KO AdvSCA-1^+^ cell migration toward SMCs. To confirm this, we used the CRISPR/Cas9 genome-editing tool to knock out *Dcn* and *Bmp1* in WT and mutant cells and then assess their migration potential (Figures 4B, S3A, and S3B). Our data showed that BMP1 absence significantly mitigated migration, while absence of DCN enhanced it. We sought to investigate whether DCN and inhibition of BMP1 could ameliorate the increased migration observed in ApoE KO AdvSCA-1^+^ cells *in vivo* (Figure 4C). When ApoE KO cells were treated with DCN and transplanted to the WT extravascular cell wall of the femoral artery, they were mostly retained in the adventitial and medial layers. BMP1 inhibitor treatment caused an even distribution of transplanted cells throughout the vascular wall. These results demonstrate that DCN and BMP1 levels influence AdvSCA-1^+^ cell migration.

Based on these findings, we hypothesized that DCN and BMP1 may induce a synergistic effect on their most prominent target, collagen I (Ge and Greenspan, 2006, Keene et al., 2000). To test this we set up 24-hr and 48-hr cell cultures of semi-confluent WT and ApoE KO AdvSCA-1^+^, from which supernatant was collected. Collagens I, II, and III and BMP1 protein levels were measured via ELISA in the presence of DCN and/or iBMP1 (Figures 4D and S3C). Protein levels in untreated cell cultures were in sync with gene expression levels (Figure 4E). Collagen expression was altered in the ApoE KO in comparison with WT. DCN had no effect on collagen I protein in the ApoE KO, while collagens II and III were affected in the WT, implying a conditioned collagen assembly dysregulation in the ApoE-deficient progenitors. Direct inhibition of BMP1 affected collagens I, II, and III of the WT progenitors only. Administration of both reagents adjusted collagen expression.

### LDL-Bound and Free Cholesterol Are Potent Inducers of AdvSCA-1^+^ Cell Migration, Inhibiting Their Differentiation

**Figure 5:**
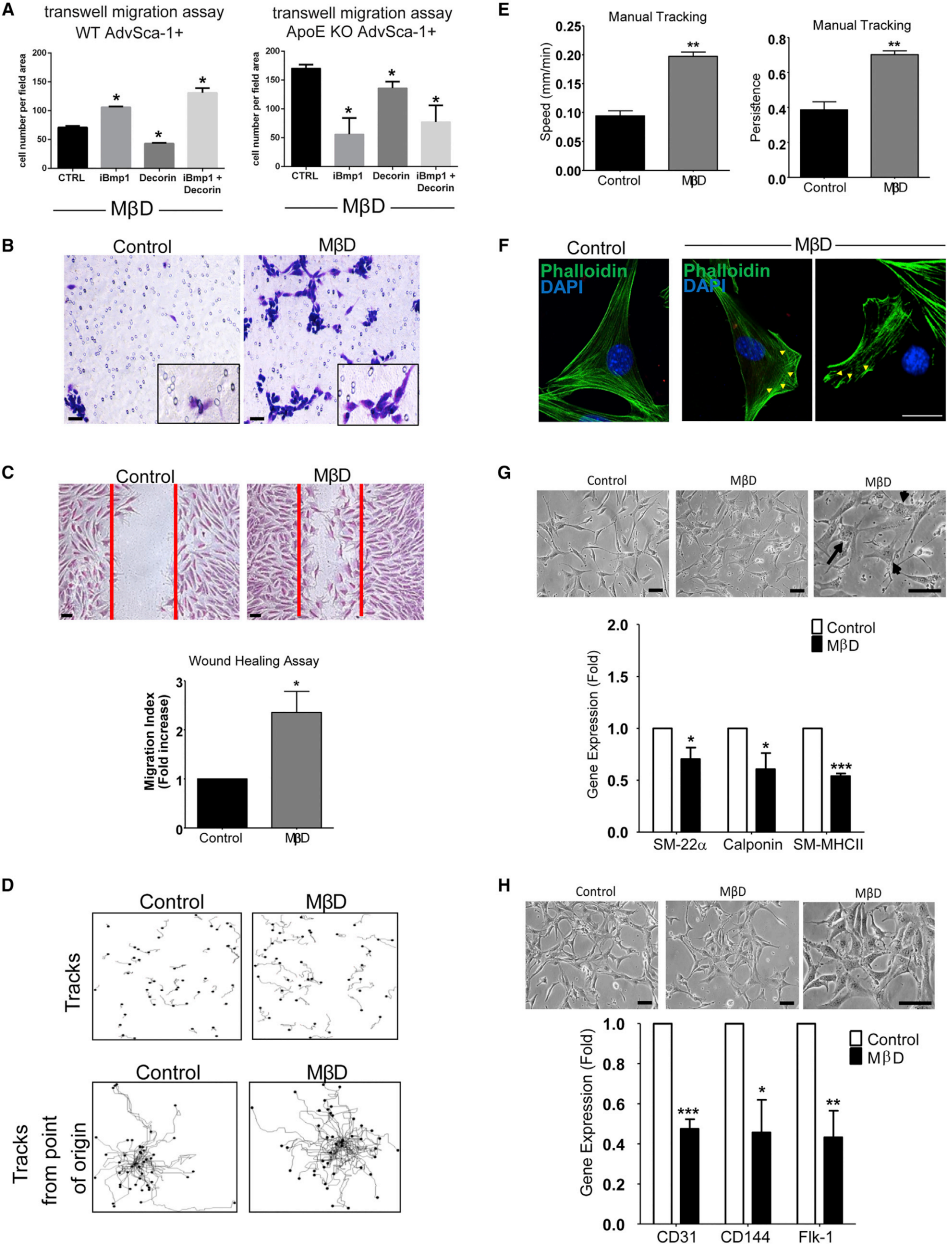
Cholesterol Induces SCA-1^+^ Cell Migration and Inhibits Differentiation (A and B) 1 × 10^5^ WT or ApoE KO AdvSCA-1^+^ cells were loaded with 20 μg/mL cholesterol along with 10 μg/mL iBMP1 and/or DCN in serum-free media and allowed to pass through 8.0-μm transwells in the presence of 0.2% FBS for 24 hr. (C) Migration of SCA-1^+^ progenitor cells was evaluated using a wound-healing assay. Migration index for both assays was defined as the mean number of progenitors counted per 5 random fields of view at 20×. (D) Vascular progenitor cells were seeded at 1 × 10^4^ cells per well of 6-well plate and subjected to 48 hr of 20 μg/mL chol-MβD loading. (E) Single-cell migration was documented using time-lapse microscopy, and quantified for speed and persistence. (F) Changes in F-actin distribution was analyzed (yellow arrowheads) using immunofluorescence staining with phalloidin (Alexa 488; green) and DAPI (blue). (G and H) The effect of cholesterol on smooth muscle cell (G) and endothelial cell (H) differentiation was observed using phase-contrast light microscopy. Black arrows represent a change in cell morphology. Total RNA was harvested for analysis of smooth muscle cell gene expression using real-time RT-PCR. Graphs are shown as mean ± SEM of three independent experiments (n = 3). ^∗^p < 0.1, ^∗∗^p < 0.05, ^∗∗∗^p < 0.01 compared with untreated control. Scale bars, 20 μm.

In ApoE KO mice hyperlipidemia contributes to atherosclerosis, with ApoE KO AdvSCA-1^+^ cells displaying enhanced cell migration. Cholesterol has been documented to be present in the adventitia (Yatera et al., 2010). To interrogate the underlying mechanisms, we loaded WT and ApoE KO AdvSCA-1^+^ with chol-MβD (water-soluble cholesterol) to evaluate the effect on progenitor cell migration. Transwell migration assays were performed, demonstrating that ApoE KO AdvSCA-1^+^ progenitors displayed enhanced migration in response to chol-MβD compared with their WT counterparts (Figures 5A and 5B); DCN ameliorated this response, while iBMP1 administration showed contrasting results. Since ApoE KO AdvSCA-1^+^ cell migration was influenced by cholesterol and low-density lipoprotein (LDL) administration (Figures S3D and S3E), we hypothesized that *in vitro* lipid loading would increase cell migration in wound-healing assays. Subsequent wound-healing assays also indicated a marked increase in the migration of chol-MβD-loaded cells when compared with untreated cells (Figure 5C). We confirmed these observations by documenting the migration of single AdvSCA-1^+^ progenitors using time-lapse microscopy showing that chol-MβD-loaded progenitor cells possessed increased migrational speed and persistence (Figures 5D and 5E). Furthermore, immunofluorescence staining of the chol-MβD-loaded progenitors revealed F-actin reorganization, particularly nearer to the leading edge of the cells, as indicated by the yellow arrowheads in Figure 5F. Treatment with modified LDLs such as oxidized LDL (Ox-LDL) and acetylated LDL (Ac-LDL) also induced AdvSCA-1^+^ cell migration, to the same extent as chol-MβD loading (Figure S3), confirming that the effects of water-soluble cholesterol administration are similar to those of natural cholesterol carriers. Additional data from experiments performed using cell counting, bromodeoxyuridine assays, and cyclin D1 expression profiling with real-time RT-PCR indicated that chol-MβD, Ox-LDL, and Ac-LDL inhibited progenitor cell proliferation (Figure S4). Together, these data suggest that cholesterol can induce SCA-1^+^ cell increased migration.

Since AdvSCA-1^+^ progenitors have a potential to differentiate to other cells that make up the vascular wall, we investigated whether chol-MβD or modified LDL loading could induce this machinery. Interestingly, these molecules did not induce differentiation into foam cells judged by their gene expression profile (Figure S5). Furthermore, the pre-treatment of progenitor cells with chol-MβD seemed to have resulted in inhibition of SMC-related (Figure 5G) and EC-related (Figure 5H) gene expression.

### Cholesterol Promotes Migration toward Pro-inflammatory Cytokines

**Figure 6:**
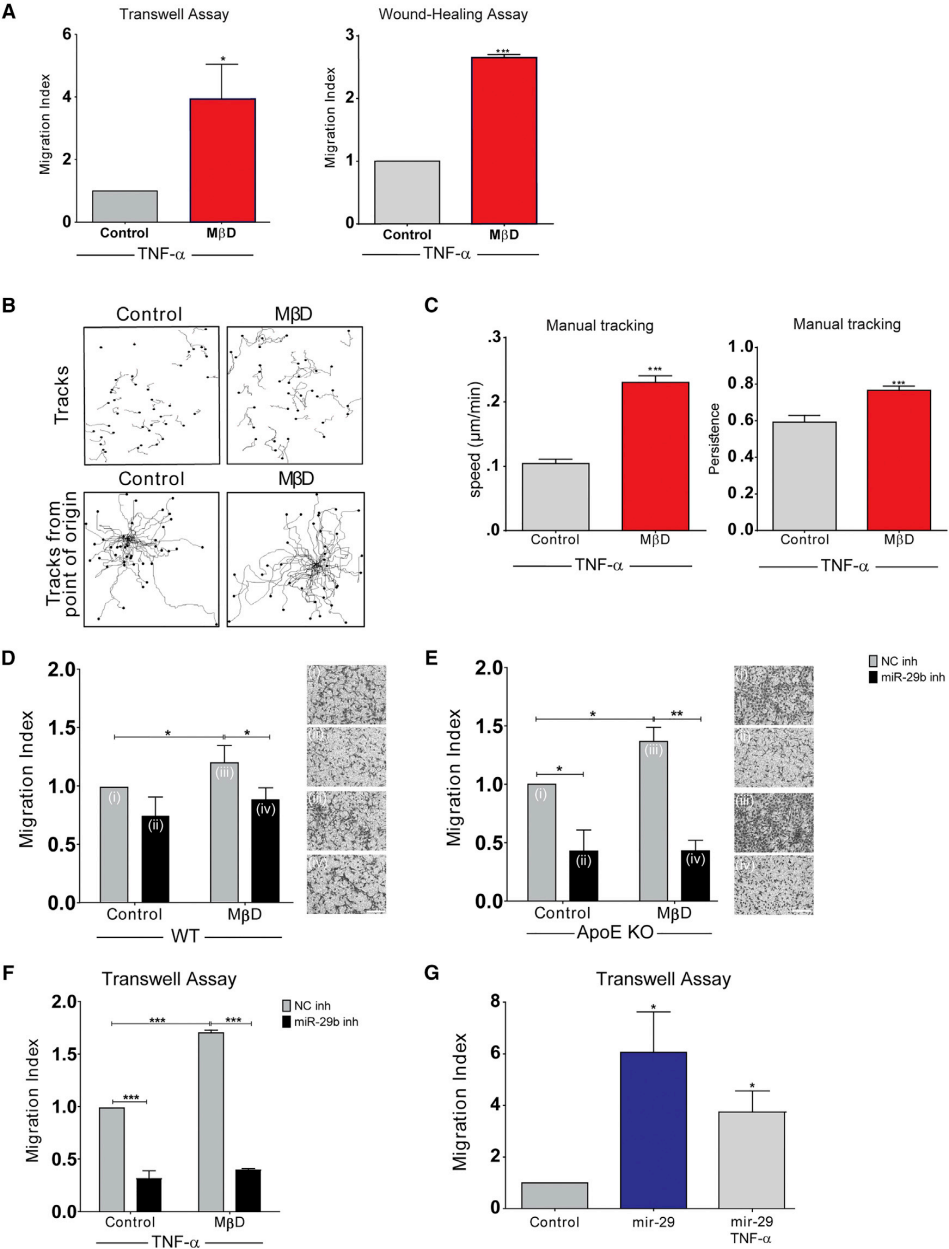
Cholesterol Can Induce SCA-1^+^ Cell Migration toward Pro-inflammatory Cytokines, along with *MicroRNA 29b* (A) Migration of vascular progenitor cells, either untreated or loaded with 20 μg/mL chol-MβD for 48 hr, toward 10 ng/mL TNF-α was evaluated using transwell and wound-healing assays. (B and C) SCA-1^+^ cells were seeded at 5 × 10^3^ cells per well of 6-well plate and subjected to 48 hr of 20 μg/mL chol-MβD loading and 10 ng/mL TNF-α. Single-cell migration was documented using time-lapse microscopy and quantified for speed and persistence. (D and E) Migration of SCA-1^+^ cell toward serum-free media, loaded with 20 μg/mL chol-MβD and in the presence of hsa-miR-29b-3p miRNA inhibitor. Scale bars, 100 μm. (F and G) Migration of SCA-1^+^ cell toward serum-free media, or loaded with 20 μg/mL chol-MβD and TNF-α (10 ng/mL) in the presence of *hsa-miR-29b-3p* miRNA and its inhibitor. Data in graphs are shown as mean ± SEM of three independent experiments. ^∗^p < 0.1, ^∗∗^p < 0.05, ^∗∗∗^p < 0.01 compared with untreated control.

In parallel, we investigated the ability of AdvSCA-1^+^ progenitors to migrate toward a panel of pro-inflammatory cytokines, coinciding with altered gene expression of their prospective surface receptors, in comparison with WT AdvSCA-1^+^ cells (Figures S6A–S6C). Although interferon-γ and granulocyte macrophage colony-stimulating factor showed a high migration index, no difference was observed in combination with chol-MβD (data not shown). Instead, SCA-1^+^ cells were able to augment their migration and locomotion in response to tumor necrosis factor α (TNF-α) combined with chol-MβD, as shown using transwell and wound-healing assays (Figure 6A). These observations were confirmed in single-cell tracking experiments using time-lapse microscopy (Figures 6B and 6C). In a similar fashion, pre-treatments with Ox-LDL and Ac-LDL were also found to induce the migration of progenitor cells in response to TNF-α (Figure S6D).

### *MiRNA-29b* Mediates Cholesterol-Induced SCA-1^+^ Cell Migration

We aimed to elucidate *microRNA* expression that drives the chol-MβD-mediated chemotactic induction of AdvSCA-1^+^ progenitors. We evaluated the potential changes of a panel of miRNA following loading with chol-MβD (Figure S7A). Chol-MβD markedly induced the expression of several miRNAs including miRNAs *29b-3p* and *32-5p*, while several miRNAs including *488-3p* were inhibited. *MicroRNA*s *10a* and *10b* are known to act in a compensatory manner (for review see Feinberg and Moore, 2016). The induction of *miRNA*s *29b-3p* and *32-5p* and inhibition of miRNA *488-3p* were subsequently confirmed using real-time PCR (Figures S7B–S7F).

*MicroRNA 29b* has been shown to participate in coronary artery disease (Chen et al., 2011). To test whether *miR-29b* is involved in the chol-MβD-mediated induction of AdvSCA-1^+^ progenitor migration, we overexpressed miRNA 29b in the AdvSCA-1^+^ progenitors by treating them with an mmu-miR-29b-3p mirVana miRNA mimic (Figure 6D). We observed that overexpression of *miR-29* significantly increased SCA-1^+^ progenitor migration, both stochastically and in the presence of TNF-α. Subsequent experiments using an miRNA29 inhibitor showed a significant reduction in *miR-29* levels and attenuated the migration of chol-MβD-mediated cells (Figure 6E). It is thus tempting to postulate that cholesterol can enhance AdvSCA-1^+^ progenitors' migratory response toward pro-inflammatory cytokines via *miR-29b* upregulation.

### Cholesterol-Induced Migration Is Mediated by Sirtuin-1 Inhibition via MMP-9 Signaling

**Figure 7:**
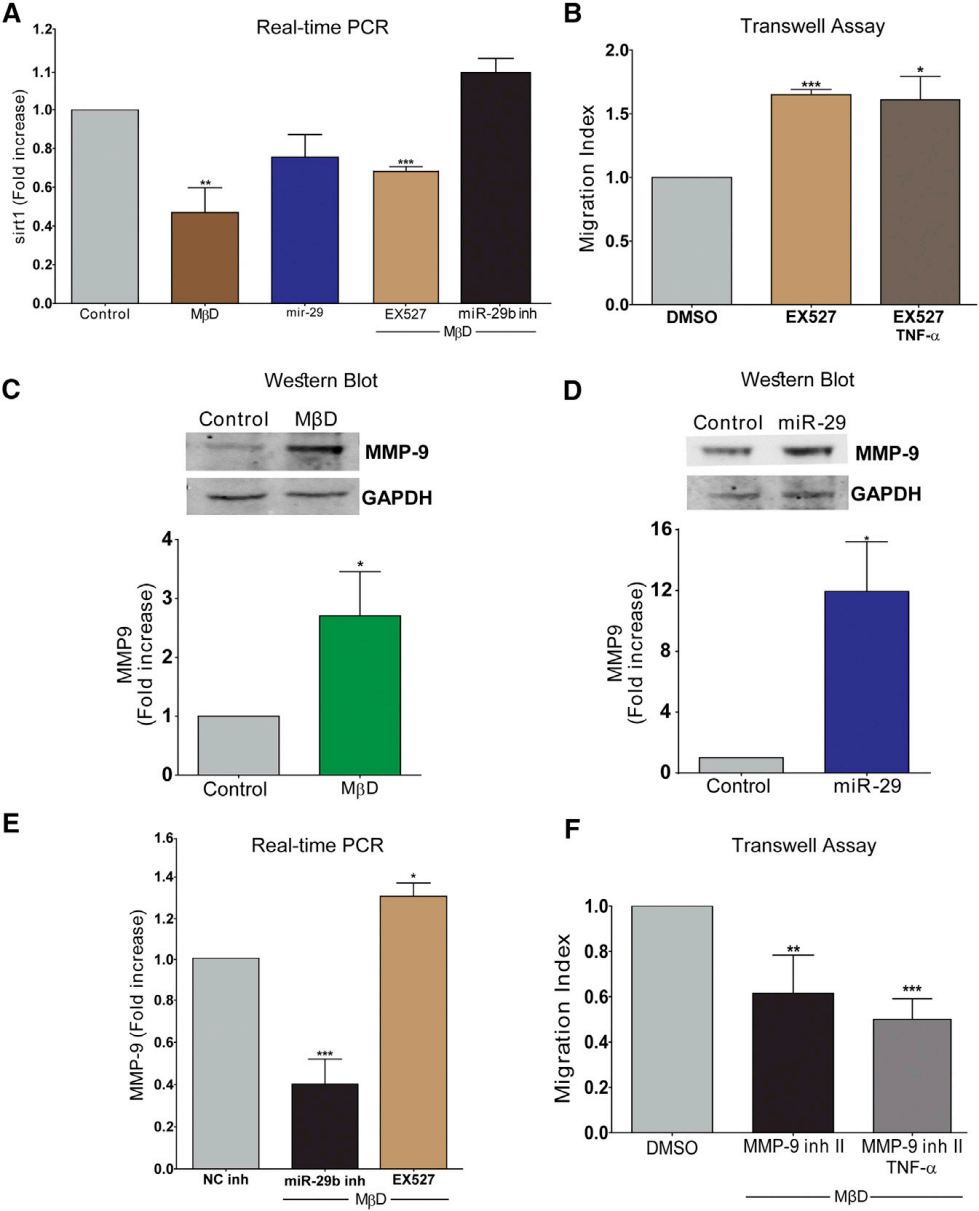
*MicroRNA 29b* Affects Cholesterol-Loaded SCA-1^+^ Cell Migration via Sirtuin-1/MMP-9 (A) SCA-1^+^ cells were loaded with 20 μg/mL chol-MβD, overexpressed with *miRNA-29*, pre-treated with 50 μM EX-527 (SIRT-1 inhibitor), or treated with 20 μg/mL chol-MβD and a hsa-miR-29b-3p ID: MH10103 mirVana miRNA inhibitor. A mirVana miRNA inhibitor Negative Control #1 or DMSO were used as controls where appropriate. Cell lysates were harvested and subjected to real-time PCR for the detection of *Sirt-1* mRNA expression. (B) Migration of SCA-1^+^ cell toward serum-free media, with TNF-α (10 ng/mL) in the presence of 50 μM EX-527 evaluated using 8.0-μm transwell assays. (C and D) Cell lysates were collected and western blotting was performed for MMP-9 detection. (E) SCA-1^+^ cells were pre-loaded with chol-MβD and treated with a hsa-miR-29b-3p ID: MH10103 *mir*Vana miRNA inhibitor or 50 μM EX-527. Cell lysates were harvested and subjected to real-time RT-PCR. (F) The effect of MMP-9 inhibition (pre-treatment with 20 μM MMP-9 inhibitor II) on the migration of chol-MβD loaded cells toward serum-free media or TNF-α (10 ng/mL) was evaluated using 8.0-μm transwell assays. Data in graphs are shown as mean ± SEM of three independent experiments. ^∗^p < 0.1, ^∗∗^p < 0.05, ^∗∗∗^p < 0.01 compared with untreated control.

We next tested whether *Sirtuin-1* (*Sirt1*) played a role in the chol-MβD-mediated effects, since this molecule has been involved in endothelial progenitor migration (Li et al., 2015). Treatment of AdvSCA-1^+^ progenitor cells with chol-MβD resulted in a suppression of *Sirt1* mRNA levels using RT-PCR (Figure 7A). Furthermore, the overexpression and inhibition of *miR-29* were observed to cause an inhibition and induction of S*irt1* expression, respectively. Additionally the inhibition of SIRT1, using a specific inhibitor (EX-527), markedly increased AdvSCA-1^+^ progenitor cell migration, both randomly and toward TNF-α (Figure 7B).

Furthermore, treatment with chol-MβD or *miR-29* caused a marked increase in matrix metalloproteinase 9 (MMP-9) expression in AdvSCA-1^+^ progenitor cells (Figures 7C and 7D). Consistently, the inhibition of *miR-29* and SIRT-1 in progenitor cells, in the presence of cholesterol, caused a reduction and increase in *Mmp-9* expression, respectively (Figure 7E). We also observed that treatment with an MMP-9 inhibitor regulated chol-MβD-mediated migration, even in the presence of TNF-α (Figure 7F). Taken together, these data suggest that chol-MβD can induce *MiR-29*, which in turn suppresses *Sirt1* and upregulates MMP-9 levels to induce AdvSCA-1^+^ progenitor cell migration.

## Discussion

Previous studies have demonstrated that adventitial fibroblasts migrate from the adventitia to the neointima upon balloon injury in rats (Li et al., 2000), while AdvSCA-1^+^ progenitors potentially participate in neointimal aggravation of vein grafts (Hu et al., 2004). Studies from our laboratory have demonstrated their potential role in native atherosclerosis (Mayr et al., 2008). A recent report using lineage-tracing analysis reported that more than 50% of neointimal SMCs derived from adventitial stem cells (Kramann et al., 2016). We provide definitive evidence that atherosclerosis-prone-derived AdvSCA-1^+^ progenitors have a different migration pattern to AdvSCA-1^+^ deriving from atherosclerosis-protected mice. In the present study, we show that hyperlipidemia triggers changes in gene expression in AdvSCA-1^+^ cells, promoting ECM remodeling and cell migration. Collagen ratios are altered in ApoE KO AdvSCA-1^+^ progenitors. Increase in collagen I/III ratio due to fatty acid excess has been shown to cause cardiac “stiffness” (Beam et al., 2015). A metabolomics analysis revealed a significant increase in fatty acid metabolites in ApoE KO AdvSCA-1^+^ progenitors (our unpublished data). The presence of pro-collagen II is an indication of the unavoidable discrepancies that appear between *in situ* and *in vitro* experimental systems. It is also quite possible that since SCA-1^+^ cells were allowed to migrate for 24 hr, in the absence of leukemia inhibitory factor, they also began producing collagen II due to differentiation.

There are several implications that derive from this study. First, *in situ* single-cell gene sequencing indicates that the majority of AdvSCA-1^+^ cells are local resident progenitor/precursor cells, since a very small proportion of AdvSCA-1^+^ displayed bone marrow cell markers. Second, water-soluble cholesterol and modified LDLs are able to alter the gene expression and function of AdvSCA-1^+^ cells related to migration. Third, by combining single-cell differential gene expression analysis with established *in vitro* and *in vivo* cell migration systems, we reveal the use of potential molecules that can regulate AdvSCA-1^+^ migration.

We specifically link DCN and BMP1 to extracellular collagen remodeling in cholesterol-loaded AdvSCA-1^+^ cells; in contrast, *microRNA 29b* induces migration, possibly via *Sirt1* (Schematic Diagram S1). DCN is a small leucine-rich proteoglycan that plays important roles in atherosclerosis (Singla et al., 2011). In Fischer et al. (2000), reported an amelioration of intimal formation upon balloon injury in rats after *Dcn* gene overexpression. Although its expression is primarily observed in the *tunica adventitia*, it affects SMC response to growth factors (Fischer et al., 2001, Nili et al., 2003, Ueda et al., 2015). Decorin overexpression reduces atherosclerosis in ApoE KO mice (Al Haj Zen et al., 2006). Here, we show that in the same mouse model there is a shift of DCN expression/secretion from the adventitia to the medial layer. Whether this is a compensatory mechanism required for neointima stabilization remains to be elucidated. Studies performed in the skin of ApoE and DCN double KOs showed an overall reduced DCN expression, similar to our findings in the aorta (Hiebert et al., 2011). In diabetic nephropathy, DCN acts in a protective manner, by reducing ECM accumulation (Merline et al., 2009); its absence attracts more mononuclear cells to the sites of insult, while in ocular fibroblasts a combination of suramin and DCN specifically inhibits collagen production (Mietz et al., 1997). A recent study in abdominal aortic aneurysm showed that DCN stabilizes the ECM and inflammation, preventing induced pathology (Ueda et al., 2015). The authors cautiously note the dual role of this molecule, promoting macrophage-induced pathology while ameliorating a vSMC-induced one. It is therefore highly likely that DCN's role in atherosclerosis is cell specific, as shown recently, in the human heart (Barallobre-Barreiro et al., 2016).

DCN has been linked to collagen synthesis and BMP1 function (Keene et al., 2000, von Marschall and Fisher, 2010). We found that *Bmp1* is overexpressed in the mutant, while *collagen I* and *collagen III* gene expression was altered. The function of BMP1 on transforming growth factor β (TGF-β) cleavage has been reported (Ge and Greenspan, 2006), while DCN also affects TGF-β's function (Ferdous et al., 2007). BMP1 is a potent pro-collagen cleavage protease that is involved in atherosclerosis (Derwall et al., 2012); in fact, its reduction decreases vascular wall calcification. We hypothesized that these molecules act synergistically in collagen production in AdvSCA-1^+^ cells. The addition of iBMP1 nearly abolished AdvSCA-1^+^ migration, with minimal cell toxicity, while DCN addition affected only ApoE KO SCA-1^+^ cells but not WT SCA-1^+^ cells. Thus, addition of DCN or DCN/iBMP1 fine-tunes ApoE KO AdvSCA-1^+^ cells into a perhaps more WT AdvSCA-1^+^ migratory (atheroprotective) behavior.

The search for cues important for AdvSCA-1^+^ migration led us to investigate a cohort of miRNAs in relation to this. Lipid loading showed that ApoE KO progenitors were indeed more prone to migrate toward SMCs, possibly due to their altered ECM gene expression profile. Recently, we reported that AdvSCA-1^+^ cells respond to vSMC-derived chemokine cues contributing to neointima formation (Yu et al., 2016). Since 1998, when the ApoE KO mouse model was employed for studies of atherosclerosis and hyperlipidemia (Hofker et al., 1998), it has not been shown how LDLs contribute to specific effects on AdvSCA-1^+^ cells. It has been recently documented that DNA hypomethylation is important for the onset of the disease, along with lifestyle factors (Jiang et al., 2012). Thus, miRNA expression levels may have a strong influence in controlling the disease. Our experiments show that *miR-29b* elicits a response to the migratory potential of lipid-loaded AdvSCA-1^+^, possibly via *Sirt1* and MMP-9, with the latter documented to play a role in atherosclerosis (Chen et al., 2008, Gough et al., 2006, Pleva et al., 2015), while the former is a molecule now shown to be involved in AdvSCA-1^+^ progenitors. Of note, it has been reported that human *MiR-29b* has been implicated in ECM remodeling including collagen regulation (Kriegel et al., 2012), while its dysregulation in murine cardiac fibroblasts upon post-myocardial infraction led to decreased collagen formation and fibrosis (van Rooij et al., 2008). It was also interesting how pro-inflammatory cytokines such as TNF-α affected this pathway, meaning that innate immunity may play a crucial role.

Atherosclerosis is a chronic inflammatory disease characterized by lipoprotein accumulation that carries cholesterol inside the arteries. Accumulation of LDL and cholesterol leads to aberrant activation of vSMCs and macrophages within the vessel wall (Xu et al., 2015). Modified LDLs have been also known to increase foam cell formation, influencing lesion development (Cox et al., 2007). Although these molecules have been implicated in pro- and anti-atherosclerotic responses, their effect on Sca-1^+^ progenitors has not been investigated. Loading of AdvSCA-1^+^ with MβD had an immediate effect on their migration potential, causing cytoskeletal rearrangements characteristic of increased locomotion. Ox- and Ac-LDL administration had a modest effect on migration, along with affecting SCA-1^+^ cell proliferation and viability; in line with these results, mRNA levels of known scavenger receptors that bind modified LDL molecules such as *Cd63*, *Cd68*, *Lox-1*, *Msra*, and others remained unchanged. We thus attempted to identify the mechanism by which chol-MβD induces SCA-1^+^ ECM modifications that induce migration. The role of MMP-9 in this process has been documented in macrophage function and arterial enlargement during atherosclerosis (Chen et al., 2008, Gough et al., 2006, Karwowski et al., 1999, Tronc et al., 2000). While this paper was being written, it was reported that *Mmp-9-deficient* mice had an abnormal cholesterol metabolism, leading to atherosclerosis (Hernandez-Anzaldo et al., 2016). Here, we show that AdvSCA-1^+^ MMP-9 levels were elevated significantly upon exposure to chol-MβD, underscoring the potential ability of AdvSCA-1^+^ to alter the ECM. Based on this, we investigated whether *Sirt1* inhibition, whose expression has been reported to inhibit atherosclerosis in vSMCs (Gorenne et al., 2013), also had an effect on SCA-1^+^ progenitor migration. Indeed, by using a specific inhibitor or loading with chol-MβD, progenitors' migration index was increased in a linearly inverted fashion. *MicroRNA 29b-3p* expression levels affected chol-MβD-loaded AdvSCA-1^+^. Two of the mechanisms of action were via a modest *Sirt1* change and a massive MMP-9 upregulation. To our knowledge, no report has shown to date that an miRNA can alter the lipid-loaded migratory effect of AdvSCA-1^+^ progenitors.

In summary, our results provide some clarity of the migratory mechanisms of resident AdvSCA-1^+^ progenitors. (1) Hyperlipidemic mice possess more HSC-derived progenitors in the adventitia, in concert with previous studies (Hu et al., 2002a, Hu et al., 2002b); (2) AdvSCA-1^+^ cells from ApoE KO mice are undergoing EMT, which could be an early sign of vascular stiffening; (3) ApoE KO AdvSCA-1^+^ cells have a higher migratory potential than their WT counterparts; finally, (4) it seems that the AdvSCA-1^+^ ApoE KO cells show an altered ECM, possibly due to dysfunctional lipid loading, which may trigger or augment an unwanted para-inflammatory immune response, a characteristic of age-related disease (Chen et al., 2012, Xu et al., 2009). Therefore, these results could provide a potential strategy for the treatment of atherosclerosis by directing adventitial cell migration.

## Experimental Procedures

### Animals

All animal procedures were performed according to the protocol reviewed by the Institution Ethics Committee and approved by the UK Home Office. All mice used were on identical genetic background.

### Single-Cell Gene Expression Analysis

Mouse adventitial cells were collected and pooled from six aortas of 6-month-old mice, for each genotype. SCA-1^+^ cells were isolated from primary cultured cells upon reaching 90% confluence using a microbeads kit (Miltenyi Biotec, Bergisch Gladbach, Germany) containing anti-SCA-1 immunomagnetic microbeads and a magnetic cell-sorting system column. Cells were further analyzed using the Fluidigm C1 machine and workflow following the manufacturer's protocol.

### AdvSCA-1^+^ Progenitor Cell Culture and Differentiation

Mouse adult progenitor cells were derived from an outgrowth of adventitial tissues of vessel grafts as previously described (Hu et al., 2004, Tsai et al., 2012).

### Cell Transplantations

For the *in vivo* cell transplantations, 1 × 10^6^ cells were immersed in Matrigel for 30 min on ice prior to transplanting them to the extra-adventitial space of the femoral artery of 6-month-old mice. Decorin and iBMP1 were administered along with the Matrigel, where appropriate. Mice were then euthanized 24 and 72 hr later, and femoral arteries were collected and fixed on 4% paraformaldehyde for 15 min prior to further analysis.

### Exogenous Proteins, Antagonists, Cholesterol, and Modified LDL Loading

For a detailed description, see Supplemental Experimental Procedures.

### Transwell Chemotaxis and Scratch-Wound Assays

For a detailed description, see Supplemental Experimental Procedures.

### MicroRNA Transient Transfection

Manipulation of miRNA levels in vascular progenitor cells (cultured to 60%–70% confluence) was carried out using an *mmu-miR-29b-3p* mouse *mir*Vana miRNA mimic (5 μM), according to the manufacturer's protocol.

## Author Contributions

I.K. designed and performed experiments, analyzed data, and wrote the majority of the paper; M.M.W. designed and performed experiments, analyzed data, and wrote part of the paper; C.M.F.P. performed experiments; Y.X. performed transplantations; B.Y. and Y.H. provided tissues; D.T.W. assisted with cell tracking; W.N.N. helped in metabolomics and cell surface analysis; A.l.B. provided reagents; Z.N. helped with experiments; C.Z., X.R., L.Z., and E.K. provided help with the study design; Q.X. conceived the major part of the study; L.Z. and Q.X. designed the whole study and obtained funding.

## Supplemental Information

Document S1. Supplemental Experimental Procedures, Figures S1–S7, and Schematic Diagram S1

Document S2. Article plus Supplemental Information
