# Supplementary material for: Adventitial SCA-1+ Progenitor Cell Gene Sequencing Reveals the Mechanisms of Cell Migration in Response to Hyperlipidemia
Source: Stem Cell Reports. 2017 Jul 27;9(2):681–96. doi: 10.1016/j.stemcr.2017.06.011 (PMC5549964; doi:10.1016/j.stemcr.2017.06.011)
Supplement: Document S1. Supplemental Experimental Procedures, Figures S1–S7, and Schematic Diagram S1 [file mmc1.pdf]

Stem Cell Reports, Volume *9*

Supplemental Information

Adventitial SCA-1$^+$ Progenitor Cell Gene Sequencing Reveals the Mechanisms of Cell Migration in Response to Hyperlipidemia

Ioannis Kokkinopoulos, Mei Mei Wong, Claire M.F. Potter, Yao Xie, Baoqi Yu, Derek T. Warren, Witold N. Nowak, Alexandra Le Bras, Zhichao Ni, Chao Zhou, Xiongzhong Ruan, Eirini Karamariti, Yanhua Hu, Li Zhang, and Qingbo Xu

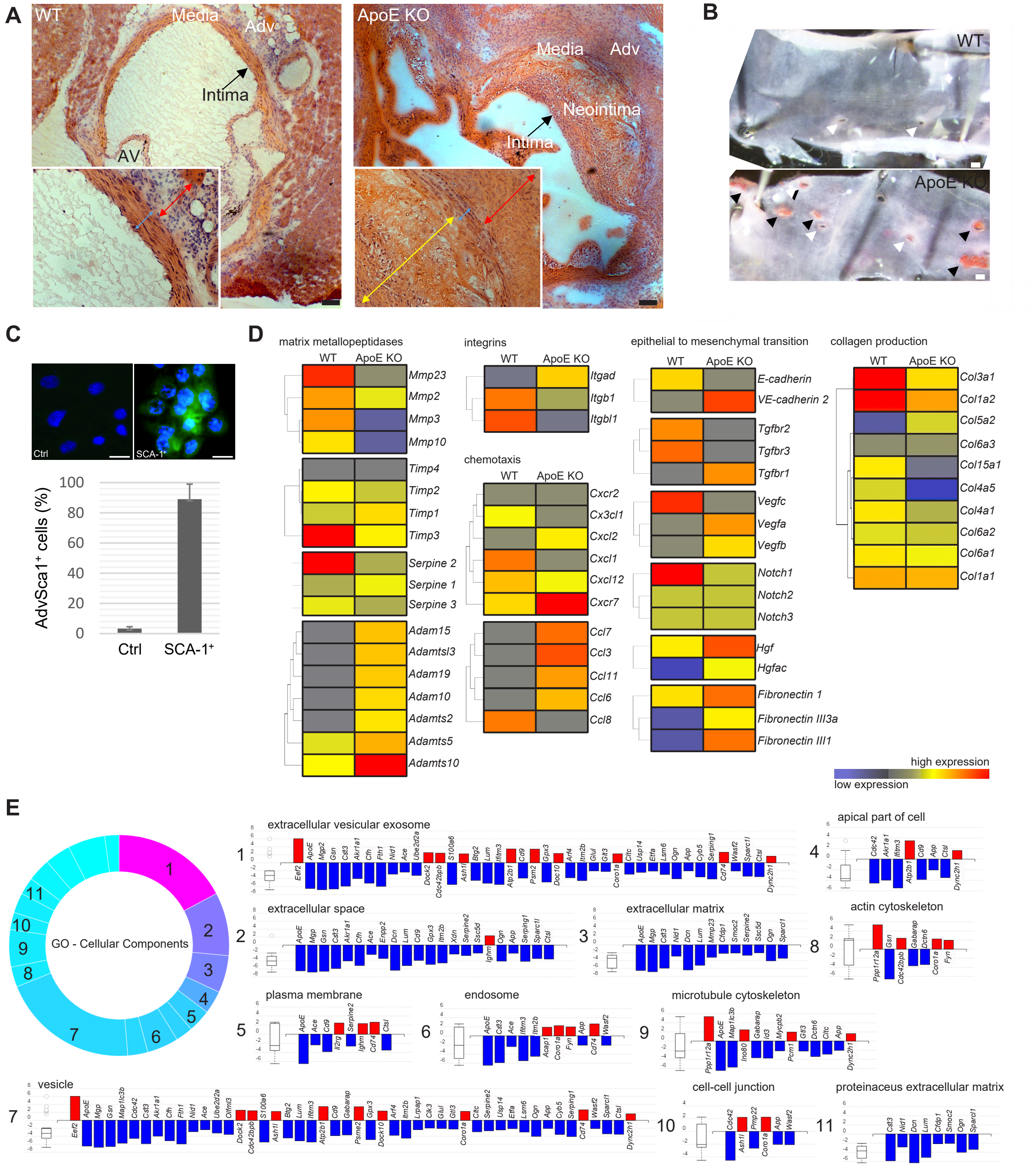

F

bone marrow HSC genes	*Ptprc (Cd45)*	*Thy-1 (Cd90)*	*Cd38*	*Cd34*	*C-kit*	*Cd105*
**WT**	N/A	N/A	N/A	**5%**	N/A	N/A
**ApoE KO**	**10%**	**5%**	N/A	**20%**	N/A	N/A

Figure S1. Single-cell gene expression profile indicates an altered response to extracellular components of adventitial ApoE KO SCA-1+ in comparison to WT AdvSCA-1+ cells

(**A**) H&E cross-sections of WT and ApoE KO aortic roots, indicating the significant morphological alteration caused in the ApoE KO 6-month old mice, with the formation of neointimal areas (black arrow), and the overall thickening of both media (yellow arrow) and adventitia (red arrow), in comparison to the WT.

(**B**) Flattened thoracic aortic tissues stained with Oil Red, indicating the lipid lesions predominantly appearing in the ApoE KO (black arrowheads).

(**C**) Immunostaining for SCA-1+ cells isolated with micro-beads. Aortic adventitial cells from ApoE KO mice were dissociated with collagenase and isolated with micro-beads coupled with an anti-SCA-1 antibody. The isolated cells were labelled with normal rat Ig (Ctrl) or anti-SCA-1 antibody, visualised with anti-rat Ig-conjugated with FITC and counterstained for nuclear visualisation. Means SEM of three experiments.

(**D**) Matrix metallopeptidases and integrins, involved in cell migration, showed an altered gene expression profile in the ApoE KO AdvSCA-1+ cell population, in comparison to the WT. Genes involved in chemotaxis were differentially expressed in the ApoE KO, while genes involved in Epithelial to mesenchymal transition (EpMT), showed that ApoE KO AdvSCA-1+ had a genotypic profile closer to a more mesenchymal than an epithelial cell, in comparison to WT. Collagen production was showed an altered gene expression pattern, predominantly affecting *Collagens I, III, IV and V*.

(**E**) GO cellular components revealed that genes involved in cell membrane responses to the extracellular environment were generally downregulated in the ApoE KO AdvSCA-1+ cell population, in comparison to the WT (Erim pruning, >5 differentially expressed genes). This indicated that the former would behave differently in the presence of different stimuli(s) from the microenvironment, than the latter.

(**F**) Haematopoietic stem cell (HSC) genes were expressed in subpopulations of AdvSCA-1+ cells, demonstrating a heterogeneous population.

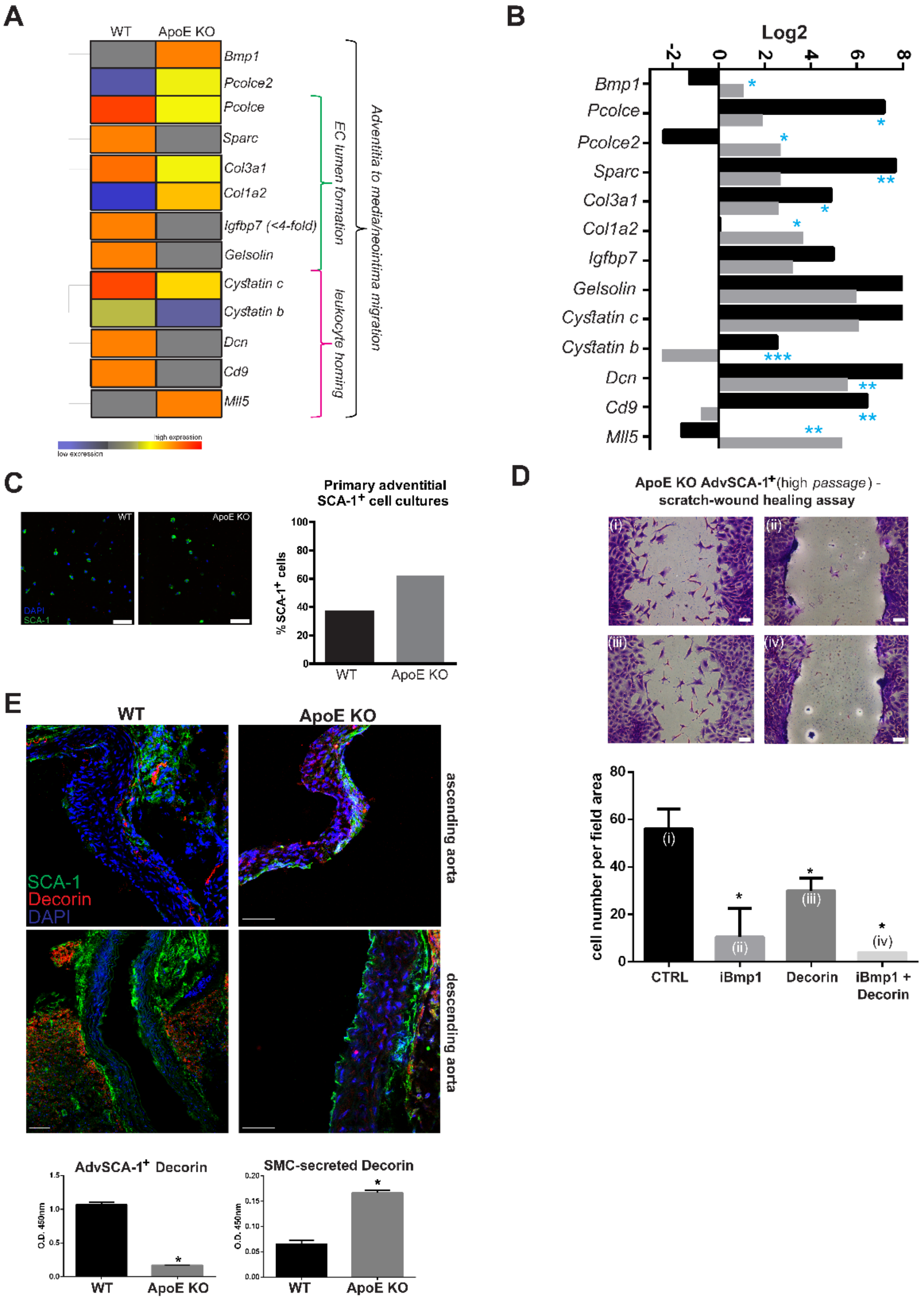

Figure S2. Potential targets for AdvSCA-1+ migration intervention

(**A,B**) Single cell differential gene expression statistical analysis revealed potential targets for Endothelial Cell (EC) lumen formation, leukocyte homing and migration. Black bars represent WT, grey bars ApoE KO.

(**C**) Confirmation of increased SCA-1+ expression in primary ApoE KO adventitia primary cells, in comparison to WT.

(**D**) Wound-healing assays of ApoE KO AdvSCA-1+ cells showed that the use of and inhibitor of BMP1 (iBMP1) with or without DCN, nearly abolished migration, with no apparent cell loss, after 24 hours in culture. DCN administration also showed a statistical decrease in cell migration.

(**E**) DCN expression is downregulated in ApoE KO AdvSCA-1+ 6-month old mice in comparison to age-matched WT animals. SCA-1 (green), DCN (red) and DAPI (blue). Sandwich ELISA indicated the decreased presence of secreted DCN *in vitro*, while in ApoE KO smooth muscle cells (SMCs), DCN was over-secreted, in comparison to WT SMCs.

Graphs are shown as mean ± SEM of three independent experiments. One Way ANOVA with Whitman test. *$*p<0.1$, $**p<0.05$ $***p<0.01$.*

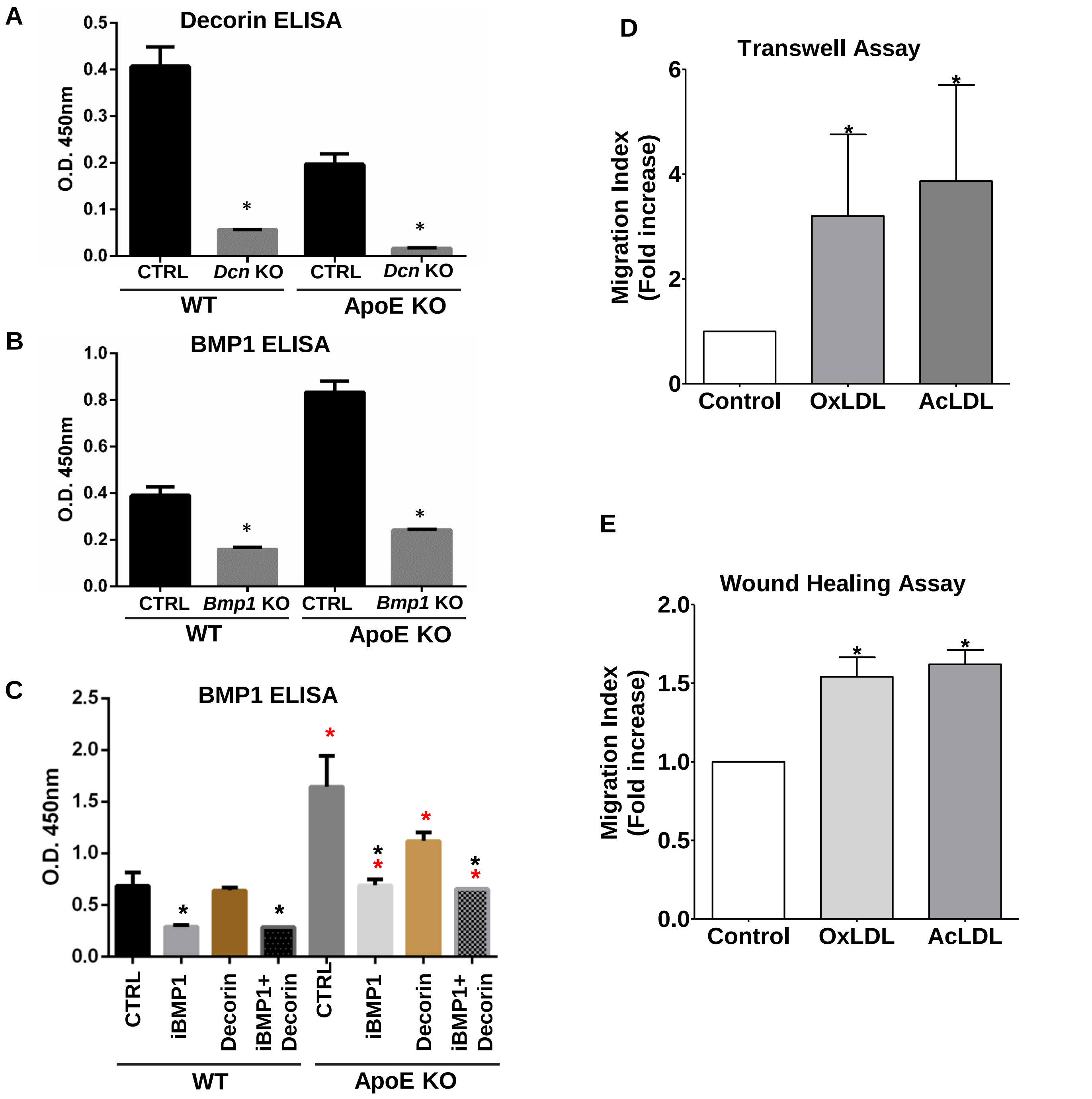

Figure S3. CRISPR KO and BMP1 ELISA assays. Oxidised-LDL and Acetylated-LDL can also induce SCA-1$^+$ cell migration.

(**A,B**) 100,000 cells WT or ApoE KO AdvSCA-1$^+$ *Dcn* and *Bmp1* KO were seeded on petri dishes. Supernatant were collected after 24 hours and ELISA assays were performed. **p<0.05*, *CTRLs* compared to *CRISPR KO*.

(**C**) 30,000 WT or ApoE KO AdvSCA-1$^+$ cells were seeded onto 12 wells plates and treated with iBMP1 and/or DCN. Supernatant were collected after 24 hours. An ELISA assay was performed against BMP1. Graphs are shown as mean ± SEM of three independent experiments. One Way ANOVA with Whitman test. **p<0.1*, compared with untreated control. **p<0.05* compared *WT* to *ApoE KO*.

(**D**) ApoE KO AdvSCA-1$^+$ were treated either with 5μg/ml Ox-LDL or 20μg/ml Ac-LDL in medium containing 0.2% FBS for 48 hours prior to migration assays. Untreated cells were used as a control. Chemotaxis of vascular progenitor cells in 8.0 μm transwells was documented following 1% crystal violet staining.

(**E**) Migration of vascular progenitor cells was evaluated using a wound-healing assay. Migration index for both assays were defined as the mean number of progenitor cells counted per 5 random fields of view at 20x.

Graphs are shown as mean ± SEM of three independent experiments. **p<0.05* compared with untreated control.

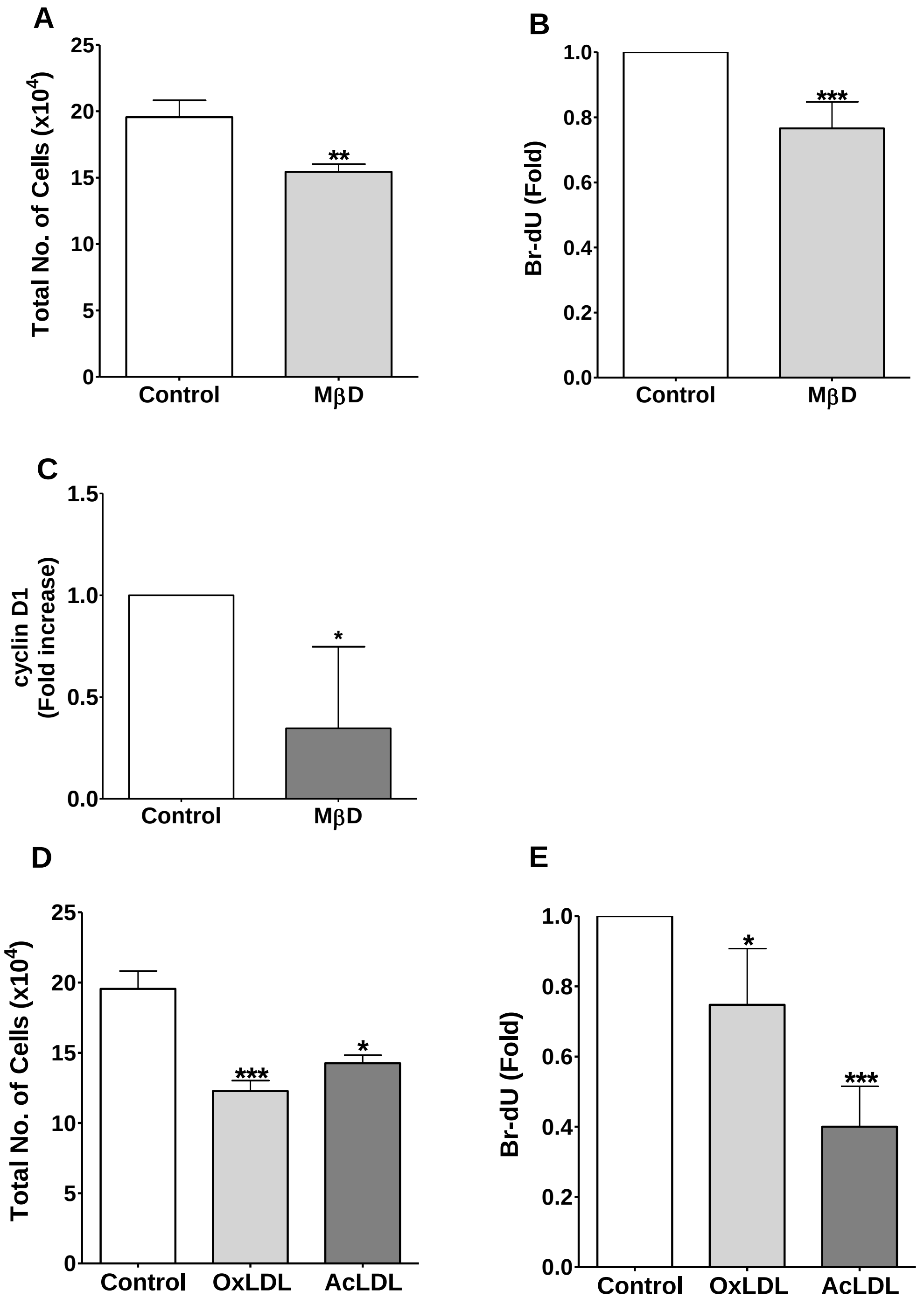

Figure S4. Cholesterol, oxidised-LDL and acetylated-LDL suppresses ApoE KO AdvSCA-1^{+} progenitor cell proliferation Evaluation of cell proliferation in response to 48 hours of chol-MβD loading, 5μg/ml Ox-LDL or 20μg/ml Ac-LDL was confirmed using (**A, D**) quantification of total cell number, (**B, E**) a BrdU incorporation assay and (**C**) detection of cyclin D1 mRNA expression using real time PCR. Graphs are shown as mean ± SEM of three independent experiments. **p<0.1, **p<0.05, ***p<0.01* compared with untreated control.

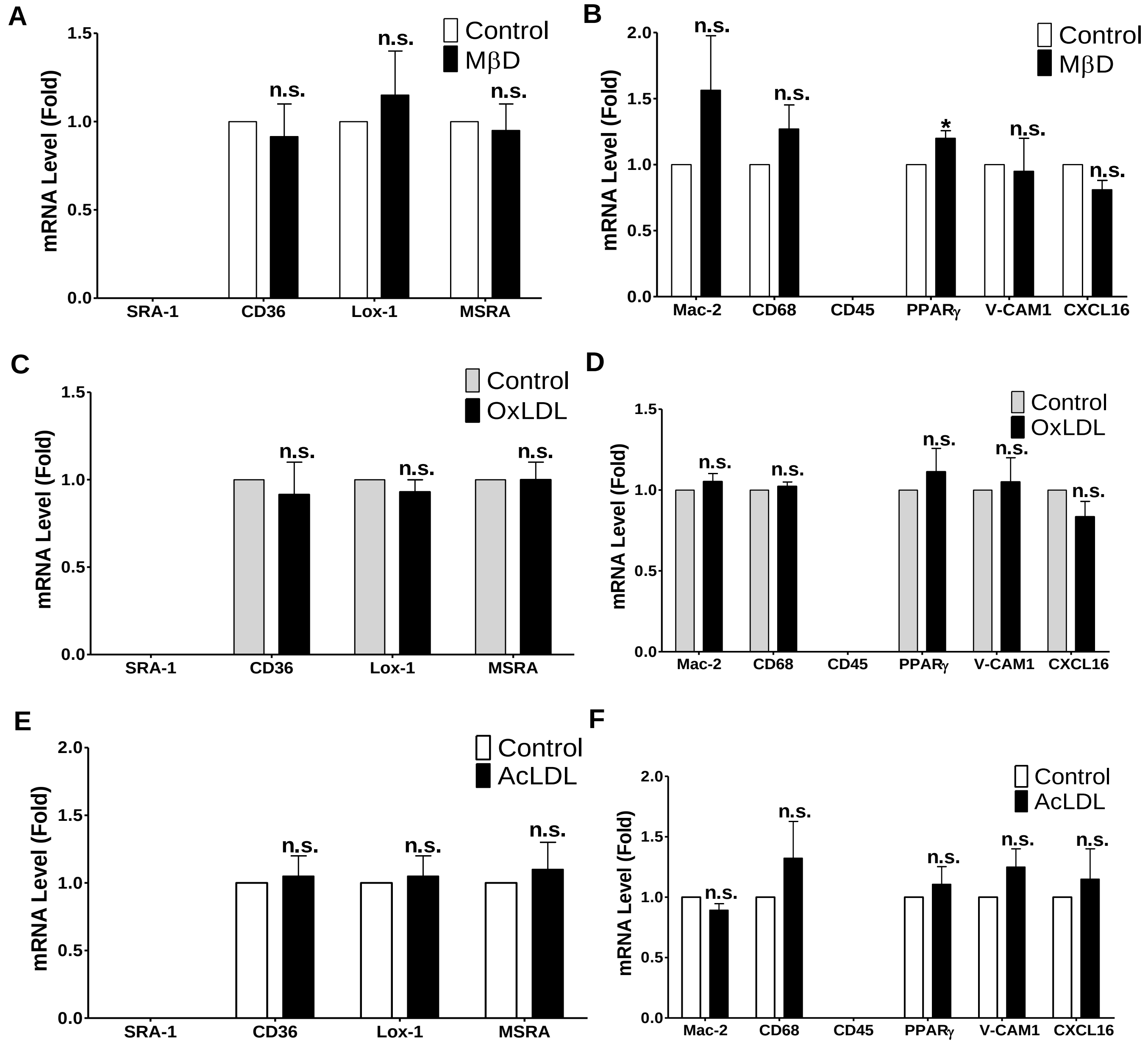

<u>**Figure S5**</u>. Neither cholesterol, Ox-LDL, or Ac-LDL induce ApoE KO AdvSCA-1$^+$ progenitor cell foam cell-differentiation or scavenger receptor gene-expression changes

(**A, B**) SCA-1$^+$ progenitor cells were loaded with 20μg/ml chol-MβD for 48 hours and cell lysates were subject to real time RT-PCR for detection of foam cell markers at the gene level.

(**C, D**) SCA-1$^+$ progenitor cells were treated for 48 hours with 5μg/ml Ox-LDL and cell lysates were subject to real time RT-PCR for detection of foam cell markers at the gene level.

(**E, F**) SCA-1$^+$ progenitor cells were treated with 20μg/ml Ac-LDL for 48 hours and cell lysates were subject to real time RT-PCR for detection of foam cell markers at the gene level.

SRA-1: steroid receptor RNA activator-1; Lox-1: oxidised low-density lipoprotein receptor-1; MSRA: methionine sulfoxide reductase A; PPAR-γ: peroxisome proliferator-activated receptor-γ; V-CAM1: vascular cell adhesion protein 1; CXCL16: chemokine (C-X-C motif) ligand 16. Graphs are shown as mean ± SEM of three independent experiments. *$p<0.05$ compared with untreated control.

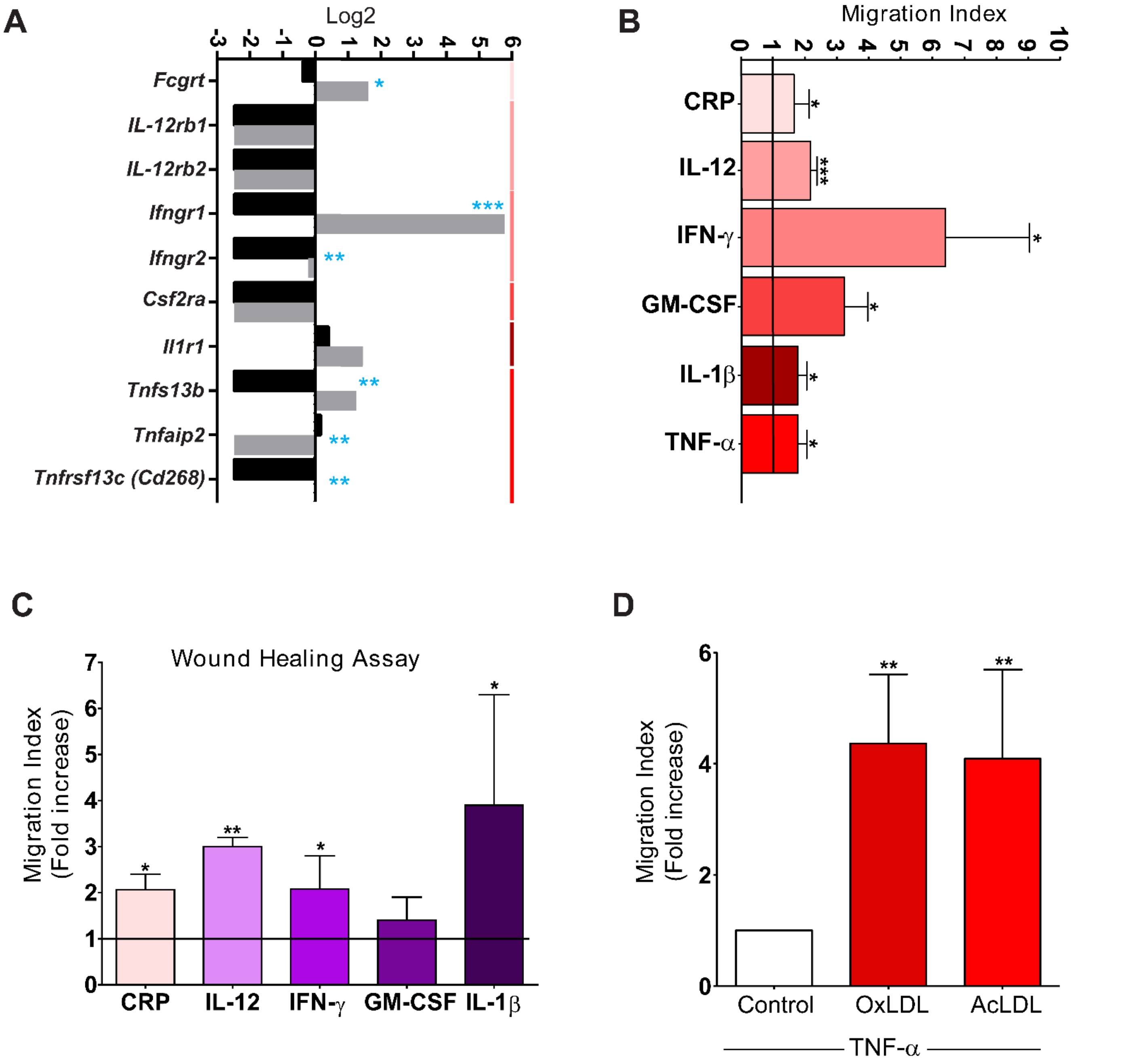

<u>**Figure S6.**</u> Cholesterol can induce SCA-1+ cell migration towards TNF-α, and a panel of pro-inflammatory cytokines

(**A**) Single-cell gene expression of surface receptors that respond to different cytokines. Black bars represent WT, grey bars ApoE KO.

(**B, C**) Migration of chol-MβD-loaded (or untreated) progenitor cells in response to either CRP (50 ng/ml), IL-12 (10 ng/ml), IFN-γ (50ng/ml), GM-CSF (50ng/ml), IL-1β (10ng/ml) and TNF-α (10ng/ml) was carried out using 8.0μm transwell and wound healing assays.

(**D**) Cells were treated for 48 hours either with 5μg/ml Ox-LDL or 20μg/ml Ac-LDL in medium containing 0.2% FBS prior to migration assays, where they were loaded with TNF-α (10ng/ml). Chemotaxis of vascular progenitor cells in 8.0μm transwells was documented following 1% crystal violet staining.

Migration index for both assays were defined as the mean number of vascular progenitor cells counted per 5 random fields of view at 20x. **p<0.1, **p<0.05, ***p<0.01*.

A

Mature miRNA ID or Gene Symbol	Fold Regulation
mmu-miR-29b-3p	1.9053
mmu-miR-30a-5p	1.5052
mmu-miR-130a-3p	1.4241
mmu-miR-27b-3p	1.5052
mmu-miR-125b-5p	1.5157
mmu-miR-32-5p	2.1287
mmu-miR-28a-5p	1.7654
mmu-miR-10a-5p	1.8277
mmu-miR-10b-5p	-1.4845
mmu-miR-99a-5p	1.6133
mmu-miR-488-3p	-1.3947
mmu-miR-141-3p	3.387

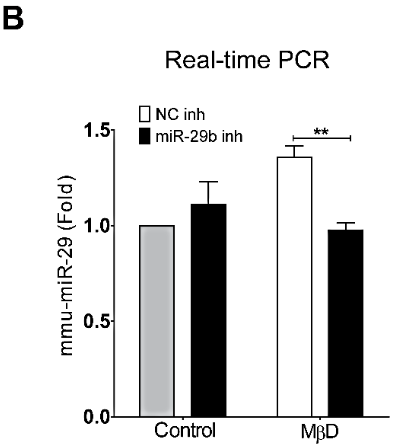

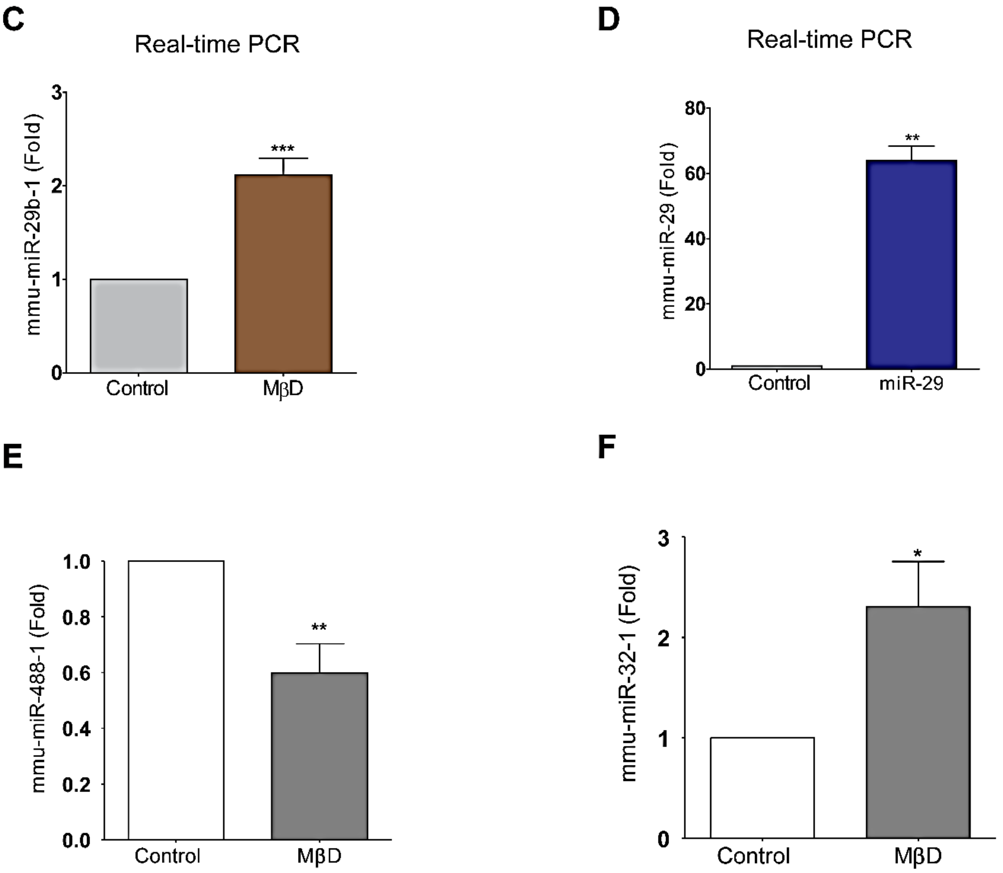

<u>**Figure S7**</u>. Cholesterol can induce SCA-1$^+$ progenitor cell migration via *miRNA-29b-1* upregulation

(**A**) SCA-1$^+$ vascular progenitor cells loaded with 20µg/ml chol-MβD for 48 hours were harvested and subjected to a miScript miRNA PCR Array Mouse miFinder. The miRNAs that showed a statistically significant difference have been highlighted.

(**B**) The induction of miRNA-29b-1 expression in progenitor cells following chol-MβD loading was confirmed using real time RT-PCR.

(**C**) miRNA-29 was over-expressed in SCA-1$^+$ progenitor cells after treatment with a mmu-miR-29b-3p mouse mirVana® miRNA mimic and confirmed using real time RT-PCR. A mirVana ™ miRNA Mimic Negative Control #1 was used as a non-targeting control.

(**D**) miRNA-29b expression in chol-MβD loaded cells was inhibited by treatment with a MH10103 mirVana™ miRNA inhibitor or a mirVana™ miRNA inhibitor Negative Control #1.

Progenitor cells were loaded with 20µg/ml chol-MβD and lysates were harvested for detection of either (**E**) miRNA-488-1 or (**F**) miRNA-32-1 expression using real time RT-PCR.

Graphs are shown as mean ± SEM of three independent experiments. *$^*p<0.1$, $^{**}p<0.05$, $^{***}p<0.01$* compared with untreated control.

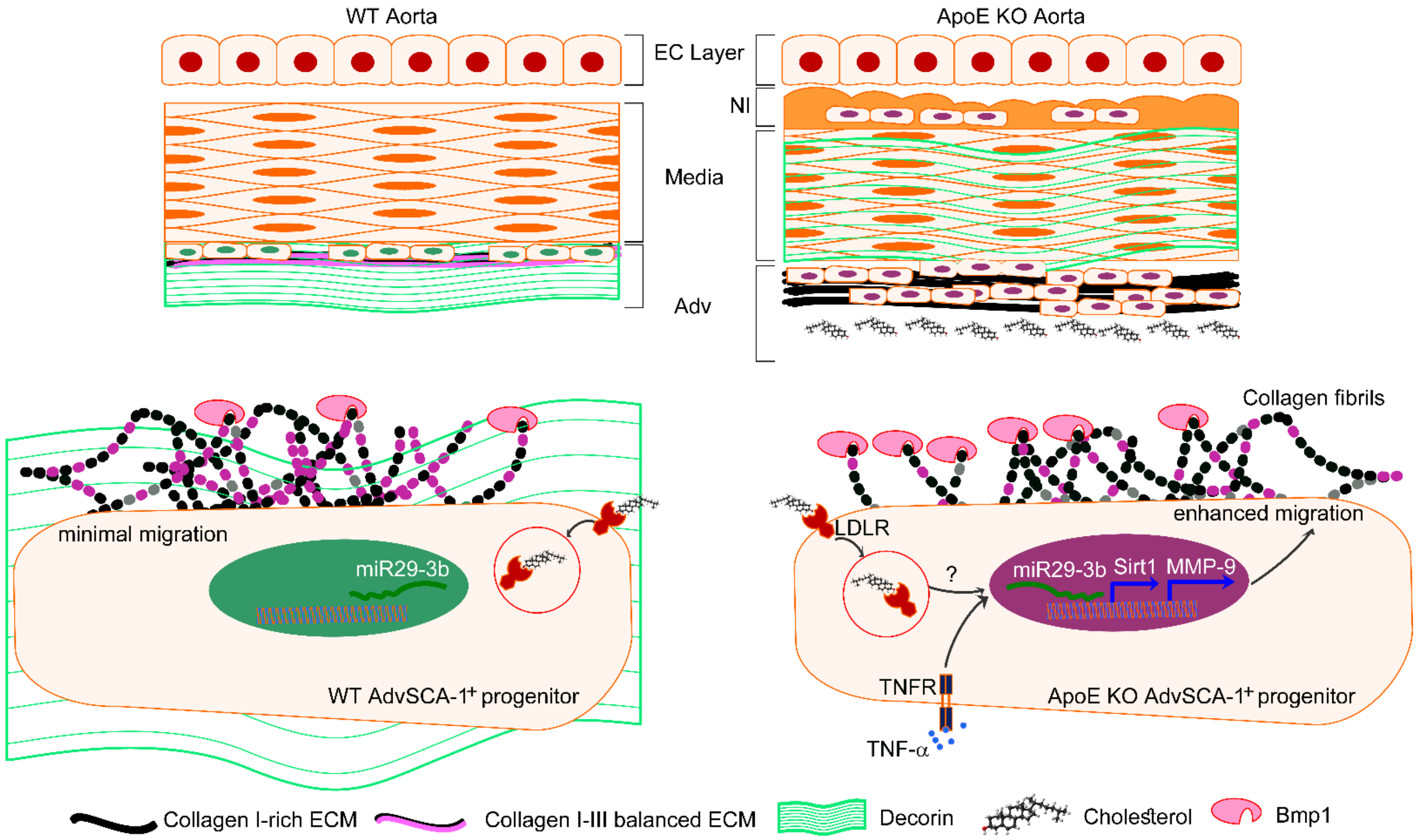

Schematic Diagram S1. Schematic representation of the vascular wall and the intracellular mechanism affecting AdvSCA-1^{+} migration.

Supplemental Experimental Procedures

Single-cell gene expression analysis

Date from Fluidigm were processed to obtain FASTAQ raw counts. RAW counts were converted to BAM files using Partek Suite, passing QA/QC. For analysis, iPathway software was used for Gene Ontology and DNASTAR Lasergene software for genome alignment (*Mus musculus* mm9) and differential gene expression, using Student’s T-test and FDR.

Vascular Progenitor Cell Culture and Differentiation

Tissue grafts were harvested with dexterity from vessel graft samples under a dissection microscope and explanted. Adventitial SCA-1$^+$ cells were obtained as described earlier. The purity of isolated AdvSCA-1$^+$ cells was confirmed using flow cytometry as shown previously (Hu et al., 2004; Xiao et al., 2007). The SCA-1$^+$ progenitor cells were cultured on 2% gelatin-coated flasks in complete stem cell medium (American Type Culture Collection, Rockville, Massachusetts, USA) containing 10% FBS, leukaemia inhibitory factor (10 ng/ml), 0.1mM 2-mercaptoethanol, penicillin (100 U/ml) and streptomycin (100 mg/ml). Smooth muscle differentiation of vascular progenitor cells was performed by culturing the cells on mouse collagen IV (5µg/ml)-coated plates in differentiation medium (DMEM supplemented with 10% FBS, 2mM L-glutamine, 100mg/l gentamicin and 0.5 mM 2-mercapotoethanol) for 5 days. Endothelial differentiation was carried out by culturing the cells on mouse collagen IV (5µg/ml)-coated plates in differentiation medium in the presence of VEGF (10ng/ml) with medium change every 2 days for 10 days. Differentiation was evaluated using real-time RT-PCR to detect the expression of either smooth muscle cell (SMC) markers such as SM-22α, Calponin, and SM-MHCII, or EC markers such as CD31, CD144 and Flk-1.

Oil Red O and H and E Staining

Aortas were obtained from WT and ApoE KO mice aged between 6 and 9 months for staining with Oil Red O. Mouse hearts and aortic roots were obtained from 6 month-old WT and ApoE KO mice and snap frozen in liquid. The tissues were mounted in OCT and sliced into 8 µm thick sections which were mounted on slides and stained with haematoxylin and eosin.

Real-time RT-PCR for RNA detection

Total RNA was isolated from vascular progenitor cells using an RNeasy Mini kit (QIAGEN Inc.) according to manufacturer’s instructions. In brief, 2 µg RNA were reverse-transcribed into cDNA with random primers by MMLV reverse transcriptase (RT) (Promega) and real time RT-PCR was performed using 2ng of cDNA per sample with a SYBR Green Master Mix in a 25-µl reaction. Ct values were measured using ABI PRISM 7000 Sequence Detector (Applied Biosystems) and GAPDH RNA was used as an endogenous control to normalize the amounts of RNA in each sample. Sequences of primer sets used in this study are as follows;
Sirt1:5'>GTAAGCGGCTTGAGGGTAAT<3’ 5’>GTTACTGCCACAGGAACTAGAG<3’,
Lox-1:5'>CAGATGTTAGCCCAGCAGAA<3' 5'>CTCCTCCTGCTCTTTGGATTT<3',
Msra:5'>GTAACAGCCAAACACCATGTC<3' 5'>GAAGCAGCCCATTCCAAATAC<3',
Mac-2:5'>AGGAGAGGGAATGATGTTGCC <3' 5'>GGTTTGCCACTCTCAAAGGG<3',
Cd68:5'>TTGGGAACTACACACGTGGGC<3' 5'>CGGATTTGAATTTGGGCTTG<3',
Cd45:5'>CAGAGCATTCCACGGGTATT<3' 5'>GGACCCTGCATCTCCATTTAT<3',
Ppar-y:5'>GAACCTGCATCTCCACCTTATT<3' 5'>TGGAAGCCTGATGCTTTATCC<3',
Cxcl16:5'>CCCTTGTCTCTTGCGTTCTT<3' 5'>TCTGGGTGCCAGAAGAAATG<3',
Cyclin D1:5'> AGACCTGTGCGCCCTCCGTA<3' 5'>GGCCAGCGGGAAGACCTCCT<3'.
Sequences of other SMC markers 28, 29, EC markers5, CD366, SRA-16 and V-CAM16 primer sets used were as previously described both by our laboratory and others, respectively. *Mmp-9* mRNA detection was based on the use of fully validated primer sequences designed and purchased from Primer Design, UK.

Immunocytochemistry

Progenitor cells were seeded in gelatin-coated chamber slides (BD Biosciences) prior to loading with 20µg/ml chol-MβD or treatment with 5µg/ml Ox-LDL and 20µg/ml Ac-LDL. Cells cultured in the absence of cholesterol or the modified LDL were used as controls. After a 48 hour incubation, the cells were fixed with 4% paraformaldehyde, permeabilized with 0.1% Triton X-100 in PBS and blocked with 10% normal swine serum (Dako). Incubation of cells with phalloidin (either Alexa Fluor® 488 or Alexa Fluor® 546) was performed at 4°C overnight, followed by at least 3 thorough washes with PBS of 5 mins each time. Cells were counterstained with DAPI (1:1000 in PBS) for 3mins at room temperature and mounted with fluorescent mounting medium (Dako) before image acquisition using an Axio Imager.M2 microscope and AxioVision Digital Imaging System (Carl Zeiss Ltd.).

Immunohistochemistry (*en face* and cryosections)

Aortas were fixed by perfusion with 4% PFA before permeabilisation with 0.2% Triton X-100. Primary antibodies were (VE-Cadherin Santa-Cruz, #sc-6458, 1:250 and SCA-1 Abcam, #ab51317, 1:100, DCN Abcam, # ab137508, 1:100) and incubations were carried out overnight at 4ºC. Secondary antibody (AlexaFluor 488 and AlexaFluor 594 1:500) incubations were for 1 hour at room temperature. The stained arch and 5 mm thick aortic rings were separated and opened on to slides before mounting in a hard set mounting medium. When dry slides were viewed using a Leica SP5 Confocal Microscope. Additionally, aortas were isolated from WT and ApoE KO mice, dissected laterally and the intima and medial layer peeled away allowing for the staining of the adventitia as previously described.

Enzyme-linked immunosorbent assays (ELISA) assays

ELISAs were employed to compare the level of proteins both in cell extracts and supernatants between WT and ApoE KO AdvSCA-1$^+$ cells, according to the manufacturers' protocols; Decorin (Abcam, #ab207618), Procollagen I alpha 1 (Abcam, #ab210579), procollagen II (MyBiosource, #MBS2000208), collagen II (MyBiosource, #MBS720538) and BMP1 (MyBiosource, #MBS2019285). All assays were sandwich ELISAs, following the manufacturers' instructions.

Exogenous Proteins, Antagonists, Cholesterol and Modified Low-Density Lipoprotein (LDL) Loading

Cholesterol (chol-MβD) was obtained from Sigma-Aldrich, whilst human copper (Cu++) oxidised (Ox-LDL) and acetylated (Ac-LDL) low-density lipoproteins were obtained from Cell Biolabs Inc. and Molecular Probes, respectively. Murine recombinant proteins of pro-inflammatory cytokines TNF-α, IL-12, IFN-Y, GM-CSF, and IL-1β were purchased from Peprotech UK. Mouse recombinant C-reactive protein (CRP) was obtained from R&D Systems. Inhibitor EX-527 (C13H13ClN2O) was purchased from Santa Cruz Biotechnology and MMP-9 Inhibitor II (C16H17F2N3O3S) from Merck Millipore. The inhibitors were used at concentrations of 50µM and 20µM, respectively.

Cholesterol was loaded into SCA-1$^+$ cells using a Chol-MβD complex obtained as "water-soluble cholesterol" that contained ≈50 mg of cholesterol/g solid (molar ratio, 1:6 cholesterol/MβD). Chol-MβD was reconstituted in dH20 and kept in the fridge for up to 7 days. Progenitor cells were incubated with Chol-MβD (20 µg/ml) for 48 hours in DMEM, alpha-MEM containing only 0.2% FBS. Progenitor cells were also incubated with either Ox-LDL (5µg/ml) or Ac-LDL 20 µg/ml) for 48 hours in medium containing 0.2% FBS. Cells incubated for 48 hours in medium (containing 0.2% FBS) without Chol-MβD, Ox-LDL or Ac-LDL served as controls for all experiments.

Western Blot Analysis

Harvested progenitor cells were lysed with IP-A buffer (25 mM Tris-HCl pH 7.5, 150 mM NaCl, 1 mM EDTA pH 8.0, 1% Triton X-100 plus protease inhibitors) and proteins were sequentially measured using the Bradford method. 30 µg of lysate was applied to SDS-PAGE before being transferred to a nitrocellulose membrane (Amersham Biosciences), followed by a standard western blotting procedure. Polyclonal antibody against MMP-9 was purchased from Santa Cruz Biotechnology, Inc. and used to detect the respective protein. The bound primary antibody was detected using an HRP-conjugated secondary antibody and an ECL detection system (Amersham Biosciences).

CRISPR/Cas9 genome editing

Dcn and *Bmp1* gene sequences were disrupted using Genescript's *Dcn* and *Bmp1* CRISPR guide RNA in the pSpCas9 BB-2A-Puro (PX459) v2.0 plasmid along with the creation of supercoiled plasmids for optimal delivery and cellular expression. For plasmid delivery (2.4 µg per cell type) to both $5x10^5$ WT and ApoE KO AdvSCA-1$^+$ cells, an 4D Amaxa nucleofector™ core unit and the reagents provided were used (program FI-115, P3 solution), following the manufacturer's protocol. Plasmid uptake and expression were tested using a constitutively expressed GFP construct (data not shown). Transduced cells were treated with 4 µg puromycin (Gibco) after plating for 48 hours. Viable cells were *passaged* >4 times to excluded possible non-integrated plasmid puromycin resistance cassette expression along with consecutive rounds of puromycin treatment. WT and ApoE KO AdvSCA-1$^+$ *Dcn* and *Bmp1* KO cells were tested for protein expression using ELISA (for DCN and BMP1) on supernatants, in order to confirm the knockdown of both proteins.

Transwell Chemotaxis & Scratch-wound Assays

Migration assays were carried out using transwell inserts with 8.0 micron pore membrane filters (Becton Dickinson Labware, USA). Progenitor cells were incubated for 48 hours in medium containing only 0.2% FBS in the absence or presence of either chol-MβD, Ox-LDL or Ac-LDL. Cells were harvested using trypsin-EDTA and subsequently loaded onto the upper chamber at $5x10^4$ cells/ml of serum free medium. The bottom chamber contained serum-free medium with either TNF-α (10ng/ml) or other pro-inflammatory cytokines (i.e. IL-12 (10ng/ml), IFN-γ (50ng/ml), GM-CSF (50ng/ml), CRP (50ng/ml) and IL-1β (10ng/ml)). After an overnight incubation, non-migrating cells on the upper side of filters were washed with care using PBS and removed using cotton tip applicators. Vascular progenitors on the underside of the membrane were fixed with 4% PFA for 10 mins before staining with 1% crystal violet solution (diluted with dH20) at room temperature for at least 10 mins. Data was expressed as the mean number of migrated vascular progenitor cells in 5 random fields of view (at 20x). For experiments

that involved inhibitors (i.e. EX-527, MMP-9 inhibitor), progenitor cells were pre-treated with the respective chemicals for an hour before loading onto transwells. Serum free medium in the bottom chamber also contained the respective antagonists/inhibitors.

Progenitor cells were seeded in a 12-well plate at $4x10^5$ cells per well in complete culture medium. Upon reaching confluency, the cells were incubated for 48 hours in medium containing 0.2% FBS in the absence or presence of either chol-MβD, Ox-LDL or Ac-LDL. The next day, a straight scratch was made using a 1ml pipette tip to generate a 'wound'. The pipette tip was kept at an angle of around 30 degrees during the scratch to ensure that scratch widths were consistent in every well. The wells were gently washed twice with PBS to remove all cellular debris as a result of the scratch prior to treatment with either TNF-α (10ng/ml) or other pro-inflammatory cytokines (IL-12 (10ng/ml), IFN-γ (50ng/ml), GM-CSF (50ng/ml), CRP (50ng/ml) and IL-1β (10ng/ml). After an overnight incubation, the migration of vascular progenitor cells into the 'wound' area was documented using a phase contrast microscope and subsequently quantified. Data was expressed as the mean number of migrated vascular progenitor cells in 5 random fields of view of the 'wound' (at 20x). For experiments that involved inhibitors (i.e. EX-527, MMP-9 inhibitor or DMSO as vehicle control), the vascular progenitor cells were pre-treated with the respective chemicals for an hour before performing the scratch. Medium that was used during the migration also contained the respective antagonists/inhibitors.

BrdU Cell Proliferation Assay

Upon reaching confluence, $SCA-1^+$ progenitors were cultured overnight in the absence (serum free medium only) or presence of either chol-MβD, Ox-LDL or Ac-LDL. Progenitor cell proliferation was quantified using a Cell Proliferation ELISA Assay, BrdU (colorimetric) (Roche) according to manufacturer's instructions. Cells were incubated in BrdU labelling solution (10μM) for 2 hours at 37°C before adding 200μl of FixDenat solution into each well for 30 mins at room temperature. The solution was removed thoroughly prior to incubation with BrdU conjugated antibody for 90 mins. After 3 washes with PBS, 100μl of substrate solution was added and incubated at room temperature until a change in colour was detected. An amount of 25μl 1M H2SO4 was immediately added to stop the reaction before measuring the absorbance at 450nm (correction at 690nm).

Single Cell Migration and Tracking using Time-Lapse Microscopy

ApoE KO $AdvSCA-1^+$ progenitor cells were seeded at $5x10^3$ cells per well of a 6-well plate and allowed to adhere to the bottom of the wells. Subsequently, the cells were loaded either with chol-MβD, Ox-LDL or Ac-LDL in the presence of medium containing 0.2% FBS for 48 hours. The plate was then placed into a microscope chamber of a fully motorised, multi-field time-lapse microscope (Eclipse TE 2000-E; Nikon) with a charge-coupled device camera (ORCA; Hamamatsu Photonics), and maintained at 37˚C and 5% CO2. Images were acquired from 10 random fields per well, every 5 minutes for 20 hours, using a 4x objective with bright field settings. Images were acquired using a Volocity software (PerkinElmer) and 10 random single cells in each field were tracked using the ImageJ manual tracking plugin (National Institute of Health). Analysis of migrational speed and persistence was performed using Mathematica 6.0 (Wolfram Research Ltd, USA) custom-written notebooks kindly provided from Professor Graham Dunn & Daniel Soong, King's College London.

Micro-RNA Extraction, Reverse Transcription and Real-Time RT-PCR

Extraction of total RNA including miRNA was carried out using a miRNeasy mini kit, according to the manufacturer's protocol. Briefly, 700µl of QIAzol Lysis Reagent was added to each sample and incubated for 5mins at room temperature. Subsequently, 140µl of chloroform was added to the tubes, shaken vigorously and incubated at room temperature for 2-3mins before centrifugation for 15mins at 12,000xg at 4˚C. The upper aqueous phase was mixed with 1.5 volumes of 100% ethanol and transferred into an RNeasy® Mini column before further centrifugation at ≥8000xg for 15s. A total of 700µl Buffer RWT was added followed by 500µl Buffer RPE twice; samples were centrifuged ≥8000xg and flow through was discarded between every step. In a new collection tube, 40µl RNase-free water was added to the mini columns and centrifuged for 1min at ≥8000xg to elute RNA.

Micro-RNA reverse transcription and amplification of 1µg of sample were performed using a miScript® II RT Kit with miScript HiSpec Buffer as described by the manufacturer. Briefly, template RNA was added to tubes containing a master mix (5x miScript HiSpec Buffer, 10x miScript Nucleics Mix, RNase-free water and miScript Reverse Transcriptase Mix). The tubes were incubated for 60mins at 37˚C, followed by 95˚C for 5mins.

A total of 1ng of cDNA (per well) was subjected to a miScript miRNA PCR Array Mouse miFinder (cat. no. MIMM 001Z) that was used in combination with a miScript SYBR Green PCR Kit to assess the expression of a panel of miRNAs and carried out according to the manufacturer's protocol. Individual miRNA of mmu-miR-29b-3p, mmu-miR-488-3p and mmu-miR-32-5p were confirmed using respective miScript miRNA PCR Arrays, also in combination with miScript SYBR Green PCR Kits. In brief, template cDNA was dispensed into individual wells of a 96-well PCR plate containing a reaction mix (2x QuantiTect SYBR Green PCR Master Mix, 10x miScript Universal Primer, 10x miScript Universal Assay and RNase-free water) before performing the cycler program (15mins at 95˚C and 15s at 94˚C, 30s at 55˚C, 30s at 70˚C for 40 cycles) on a

qPCR machine (Eppendorf Mastercycler® ep Realplex2, Eppendorf UK). All reagents were otherwise purchased from Qiagen.

Micro-RNA Transient Transfection

An mmu-miR-488-3p mouse mirVana® miRNA mimic (5µM), a mmu-miR-32-5p mouse mirVana® miRNA mimic (5µM), a mmu-miR-29b-3p mouse mirVana® miRNA mimic (5µM), or a non-targeting control (mirVana™ miRNA Mimic Negative Control #1, 5µM) (Ambion Life Technologies). Inhibition of miR-29b was performed using a hsa-miR-29b-3p ID: MH10103 mirVana™ miRNA inhibitor (60nM) or a mirVana™ miRNA inhibitor Negative Control #1 (60nM) (Ambion Life Technologies). All transfections were performed on SCA-1^+ progenitor cells seeded at $2x10^5$ cells/well of 6-well plates using Lipofectamine™ RNAiMAX (Invitrogen), according to the manufacturer's protocol.

Quantification of Mean Cell Size

ApoE KO AdvSCA-1^+ progenitor cells were seeded in a $T75cm^2$ flask at $5x10^5$ cells and cultured complete medium. The next day, the cells were cultured overnight in the absence (serum free medium only) or presence of either chol-MβD (cholesterol), Ox-LDL or Ac-LDL. The cells were then trypsinized and subjected to cell size calculation using a Multisizer 3 Coulter Counter (Beckman Coulter) according to the manufacturer's instructions.

Statistical Analysis

All data in this study were presented as the mean and standard error of the mean (SEM) of at least three separate experiments. The analysis was performed using GraphPad Prism V.4 (GraphPad Software, San Diego CA) using statistical analysis with analysis of variance (ANOVA) followed by Dunnett's multiple comparison tests, or Student's T-Test. Significance was considered when $p<0.05$. For Figures 3 and 4, a 2-way ANOVA was performed for comparison between similar time points or treatments against the untreated control. For Figure 4B, a Student's one-tailed T-Test was performed.

Supplemental References

Hu, Y., Zhang, Z., Torsney, E., Afzal, A.R., Davison, F., Metzler, B., and Xu, Q. (2004). Abundant progenitor cells in the adventitia contribute to atherosclerosis of vein grafts in ApoE-deficient mice. J Clin Invest *113*, 1258-1265.
Xiao, Q., Zeng, L., Zhang, Z., Hu, Y., and Xu, Q. (2007). Stem cell-derived SCA-1+ progenitors differentiate into smooth muscle cells, which is mediated by collagen IV-integrin alpha1/beta1/alphav and PDGF receptor pathways. Am J Physiol Cell Physiol *292*, C342-352.
