# Supplementary material for: Adventitial SCA-1+ Progenitor Cell Gene Sequencing Reveals the Mechanisms of Cell Migration in Response to Hyperlipidemia
Source: Stem Cell Reports. 2017 Jul 27;9(2):681–96. doi: 10.1016/j.stemcr.2017.06.011 (PMC5549964; doi:10.1016/j.stemcr.2017.06.011)
Supplement: Document S2. Article plus Supplemental Information [file mmc2.pdf]

Stem Cell Reports
Article

OPEN ACCESS

Adventitial SCA-1$^+$ Progenitor Cell Gene Sequencing Reveals the Mechanisms of Cell Migration in Response to Hyperlipidemia

Ioannis Kokkinopoulos,[1,4] Mei Mei Wong,[1,4] Claire M.F. Potter,[1,4] Yao Xie,[1] Baoqi Yu,[1] Derek T. Warren,[1] Witold N. Nowak,[1] Alexandra Le Bras,[1] Zhichao Ni,[1] Chao Zhou,[2] Xiongzhong Ruan,[2] Eirini Karamariti,[1] Yanhua Hu,[1] Li Zhang,[3,*] and Qingbo Xu[1,*]

[1]Cardiovascular Division, King's College London BHF Centre, 125 Coldharbour Lane, London SE5 9NU, UK
[2]John Moorhead Research Laboratory, Centre for Nephrology, University College London, Rowland Hill Street, London NW3 2PF, UK
[3]Department of Cardiology, The First Affiliated Hospital, Zhejiang University, 79 Qingchun Road, Hangzhou 310003, China
[4]Co-first author
*Correspondence: li_zhang@zju.edu.cn (L.Z.), qingbo.xu@kcl.ac.uk (Q.X.)
http://dx.doi.org/10.1016/j.stemcr.2017.06.011

SUMMARY

Adventitial progenitor cells, including SCA-1$^+$ and mesenchymal stem cells, are believed to be important in vascular remodeling. It has been shown that SCA-1$^+$ progenitor cells are involved in neointimal hyperplasia of vein grafts, but little is known concerning their involvement in hyperlipidemia-induced atherosclerosis. We employed single-cell sequencing technology on primary adventitial mouse SCA-1$^+$ cells from wild-type and atherosclerotic-prone (ApoE-deficient) mice and found that a group of genes controlling cell migration and matrix protein degradation was highly altered. Adventitial progenitors from ApoE-deficient mice displayed an augmented migratory potential both *in vitro* and *in vivo*. This increased migratory ability was mimicked by lipid loading to SCA-1$^+$ cells. Furthermore, we show that lipid loading increased *miRNA-29b* expression and induced sirtuin-1 and matrix metalloproteinase-9 levels to promote cell migration. These results provide direct evidence that blood cholesterol levels influence vascular progenitor cell function, which could be a potential target cell for treatment of vascular disease.

INTRODUCTION

Atherosclerosis is a condition whereby the arteries supplying target organs, such as the brain and the heart, become occluded. It is a disease of chronic inflammation and the leading cause of heart ischemia (Lusis, 2000). It is characterized by subendothelial retention of modified lipoproteins, causing local inflammation and neointimal lesion formation (Williams and Tabas, 1995). This local effect exacerbates the activation and consequent loss of the endothelial cell (EC) layer and profound vascular smooth muscle cell (vSMC) proliferation, as well as recruitment of monocytes and activation of resident macrophages. Consequently, all three layers that comprise the vascular cell wall (adventitia, media, and EC) undergo remodeling with an accumulation of cellular and extracellular matrix (ECM) material. Over the last decade, vascular progenitors have been identified in the vessel wall in humans and rodents (Alessandri et al., 2001; Ferreira et al., 2007; Hill et al., 2010; Hu et al., 2004; Invernici et al., 2008; Passman et al., 2008; Psaltis et al., 2014; Sirker et al., 2009; Tang et al., 2012; Tavian et al., 2005; Tilki et al., 2009; Wu et al., 2008; Xu et al., 2003; Zengin et al., 2006). The murine adventitia contains a heterogeneous population of progenitor/precursor cells with the majority of them expressing stem cell antigen 1 (SCA-1). In atherosclerotic arteries, the adventitial layer displays increased cell proliferation and inflammation (for review see Seidelmann et al., 2014). Recent studies have highlighted the potential role of the adventitia in the development of neointimal lesion of vessel grafts (Chen et al., 2013; Wong et al., 2013). However, less is known about the involvement of vascular stem/progenitor cells in native atherosclerosis, the leading cause of cardiovascular death in the general population.

Resident vascular progenitors possess a bilineage potential, able to differentiate into vSMCs and ECs, both *in vitro* and *in vivo* (Hu et al., 2004; Torsney et al., 2005; Wong et al., 2013). Utilization of an SM22-LacZ mouse model showed that SCA-1$^+$β-gal$^+$ cells could be traced to neointimal lesions, confirming their differentiation toward an SMC-like phenotype. When adventitial SCA-1$^+$ (AdvSCA-1$^+$) cells were applied to the external side of vein grafts, prior to isografting to ApoE knockout (KO) and wild-type (WT) mice, they were shown to contribute to lesions via unknown mechanisms.

These murine SCA-1$^+$ cells have been identified as the largest vascular progenitor cell population to date, with subpopulations expressing C-KIT and CD34 (Hu et al., 2004). Bone marrow-derived circulating cells may serve as a second potential source of vascular progenitors. In support of this, SCA-1$^+$ cells also express the macrophage/monocyte marker CD45 (Psaltis et al., 2014). Noseda et al. (2015) reported that there are at least two distinct types of SCA-1$^+$ cells in the adult mouse heart, with one segregating to a cardiogenic potential and the other toward a vascular one. Previously, we demonstrated that SCA-1$^+$-derived vSMCs present in neointimal lesions originated from non-bone marrow-derived resident vascular

CrossMark
This is an open access article under the CC BY license (http://creativecommons.org/licenses/by/4.0/).

progenitors (Hu et al., 2002a, 2002b; Xu et al., 2003). Although adventitial progenitors have been proposed to contribute to vascular disease (Campagnolo et al., 2015; Chen et al., 2013; Hu et al., 2004; Passman et al., 2008; Psaltis et al., 2014; Tigges et al., 2013), quantitative data on the contribution of local resident versus bone marrow-derived progenitors is still lacking.

For clarifying the role of AdvSCA-1$^+$ progenitors in native atherosclerosis, it is essential to elucidate their differential gene expression profile between atherosclerosis-resistant and atherosclerosis-susceptible mice. In identifying which pathways are altered during naturally occurring atherosclerosis, we may better understand their potential contribution to neointimal lesion development and maintenance. Here, we employed single-cell gene expression analysis of AdvSCA-1$^+$ cells isolated from the adult aorta of WT and ApoE KO mice. Our sequencing analysis revealed that ApoE KO SCA-1$^+$ cells have an altered gene expression profile for cytoskeletal rearrangements compared with WT SCA-1$^+$ cells, making them more receptive to extrinsic migratory cues. Subsequent mechanistic analysis with a focus on ApoE KO SCA-1$^+$ progenitors identified a potential cell-autonomous mechanism involving *microRNA* in ameliorating adventitial progenitor migration that could be of use in a clinical setting.

RESULTS

A Gene Signature of a Heightened Migratory Capability in ApoE KO AdvSCA-1$^+$ Cells

WT mice do not develop atherosclerosis (Figure S1A). The ApoE KO mice develop neointimal lesions and endothelial layer lipid residues, along with an expanded adventitial layer from 6 months onward (Figure S1B). The adventitia and intima layers have been shown to contain SCA-1$^+$ cells (Hu et al., 2004). Immunolabeling of the descending aortas and the root of WT (Figure 1A) and ApoE KO aortas revealed an increase of SCA-1$^+$ signal in both the adventitial and intimal layers of the mutant vascular wall (Figure 1B), as well as *in vitro* (Figure S2C). We confirmed this increase by *en face* immunolabeling of both descending and ascending aortas (Figures 1C–1E). To address the innate heterogeneity of the AdvSCA-1$^+$ cell population, we collected unpassaged adventitial cells from both WT and ApoE KO mice, obviating potential bias from *in vitro* expansion. These cells were then enriched for SCA-1 surface expression prior to single-cell expression analysis. A total of 25,596 genes were analyzed between the two SCA-1$^+$ cell populations (Figure 2A) and statistical analysis revealed 408 clustered genes that were significantly differentially expressed (Figure 2B). Gene Ontology (GO) pathway enrichment analysis revealed four predominant pathways being altered: cell migration, cytoskeletal organization, regulation of locomotion, and endopeptidase activity (Figure 2C). Enrichment for cellular components indicated several pathways being affected, especially concerning ECM organization and maintenance, including the exosome pathway (Figure S1C).

Potential epithelial migration, namely that of AdvSCA-1$^+$ progenitor cell migration toward the inner vascular wall, depends on the activity and gene expression of molecules able to disrupt the ECM, as well as promoting cytoskeletal rearrangements, including cell polarity. Our single-cell expression analysis revealed that matrix metallopeptidases, integrins, and collagen gene expression differed between ApoE KO and WT while AdvSCA-1$^+$ cells in the mutant setting suggested a heightened epithelial-to-mesenchymal transition (EMT) (Figure S1D). Of note, 5% of WT AdvSCA-1$^+$ cells expressed bone marrow hematopoietic progenitor genes, compared with 20% of the ApoE KO cells, from which a subpopulation of 10% was also positive for *Cd45*, 20% positive for *Cd34*, and 5% positive for *Thy1* genes, as reported previously (Figure S1E; for review see Bobryshev et al., 2015).

ApoE KO AdvSCA-1$^+$ Enhanced Migration Is Intrinsic

To address any mechanisms that allow mutant AdvSCA-1$^+$ cells to migrate more prominently, we first enriched our single-cell expression analysis for those AdvSCA-1$^+$ cells with a high copy of *Sca-1* (Figure 2D), since in our established *in vitro* system setting passaged cells are used, which are highly enriched for SCA-1 through multiple magnetic bead cell sorting (Mayr et al., 2008; Yu et al., 2016). Our findings were analyzed using genes known to correlate with epithelial mouse cell expression (Seo et al., 2010; Simpson et al., 2008) (Figure 2E). The ApoE KO's epithelial migration gene expression plexus was diverted in comparison with WT. To confirm this, we transduced WT and ApoE KO AdvSCA-1$^+$ with an RFP lentiviral construct to permanently label the cells and their progenies, if any. WT and ApoE KO AdvSCA-1$^+$-RFP cells were transplanted into the outer wall of the femoral artery of adult WT and ApoE KO mice in all four combinations (Figure 3A). Mice were then euthanized 24 or 72 hr later. In comparison with the WT, ApoE KO AdvSCA-1$^+$ displayed a greater tendency to migrate into the ApoE KO femoral artery. WT AdvSCA-1$^+$ cells migrated poorly into the ApoE KO femoral artery (Figure 3B).

We assessed whether specific genes are differentially expressed between the ApoE-derived AdvSCA-1$^+$ cells compared with their WT counterparts (Figures S2A and S2B). Thirteen genes identified were known to have an effect on migration; six of them were also important for EC lumen formation, and five were important for leukocyte homing. Of those, *Cystatin c*, *Bone morphogenic protein 1*

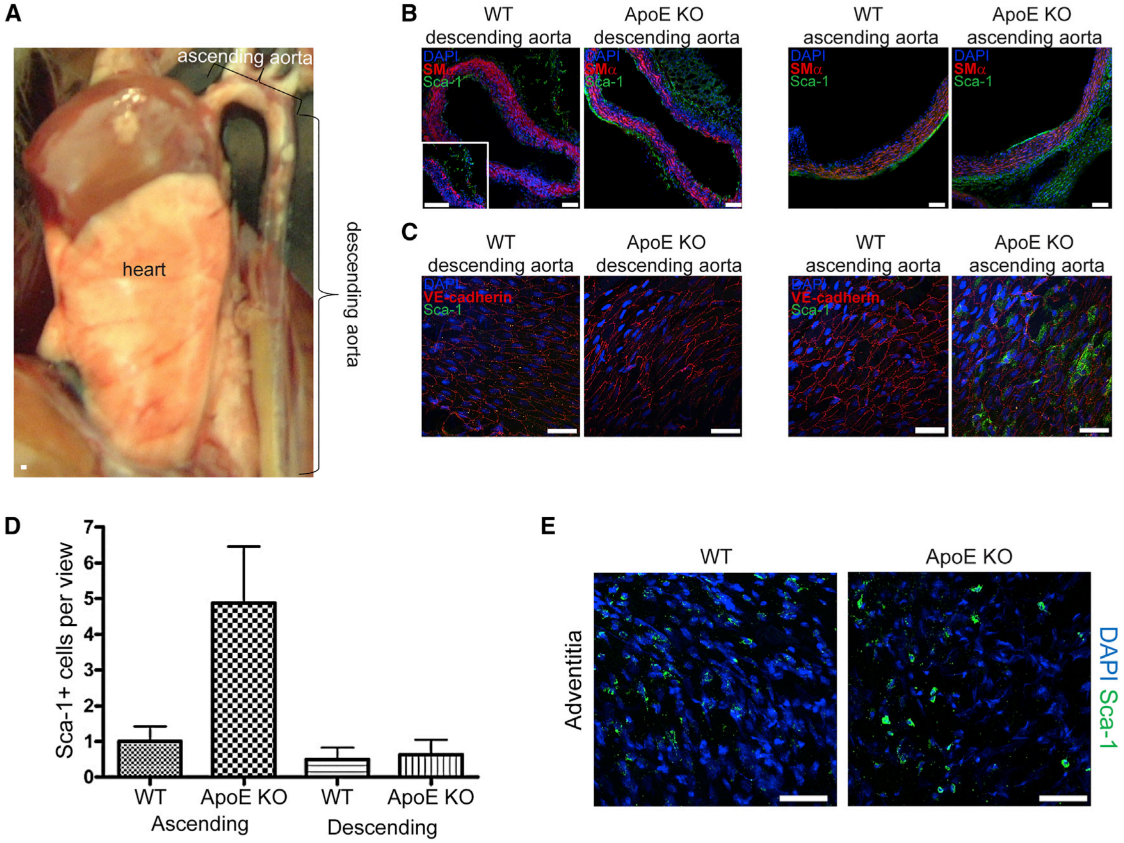

Figure 1. Adventitial ApoE KO SCA-1$^+$ Cell Distribution Differs from That of WT

(A) Microphotograph of a 6-month-old mouse WT abdominal aorta and heart.

(B) Immunohistochemical labeling of WT and ApoE KO thoracic and root aortas for SCA-1 (green) and smooth muscle cell actin (SMα, red). Inset: magnified photo of WT adventitia (n = 3 mouse aortas).

(C) *En face* immunohistochemical labeling of WT and ApoE KO ascending and descending EC layer for SCA-1 (green) and endothelial cell marker (VE-cadherin) (n = 4 mouse aortas).

(D) Bar graph showing the number of SCA-1$^+$ cells in both WT and ApoE KO EC layers of ascending and descending aortas (n = 14 independent experiments). Student's t test, $p < 0.01$.

(E) *En face* immunohistochemical labeling of the adventitia of the descending aorta of WT and ApoE KO 6-month-old mouse SCA-1 (green), with notably more cells labeled in the ApoE KO adventitia (n = 3 mouse aortas).

Scale bars, 10 μm.

(*Bmp1*), and *Decorin* (*Dcn*) were selected for further investigation of AdvSCA-1$^+$ cell migration. Since *Bmp1* expression was elevated, we assessed whether soluble Decorin (DCN) and a BMP1-specific inhibitor ($C_{15}H_{24}N_4O_4$, iBMP1) would be able to modulate cell migration in a wound-healing assay (von Marschall and Fisher, 2010) (Figure S2D). DCN was detected in the adventitia of WT aortas (Al Haj Zen et al., 2006), confirming the single-cell data outcome (Figure S2E). In contrast, DCN expression was observed in the medial layer of the ApoE KO in SMCs. This was confirmed by ELISA on WT and ApoE KO AdvSCA-1$^+$ as well as in SMCs, *in vitro*, in concert with our single-cell expression profiling. DCN and iBMP1 both decreased the migration of ApoE KO AdvSCA-1$^+$ cells when administered individually and in combination. We then tested the effect of these substances in transwell migration assays (Figure 4A). Cystatin C had no effect on migration (data not shown), while iBMP1 affected both mutant and WT AdvSCA-1$^+$ cells. DCN influenced ApoE KO AdvSCA-1$^+$ cell migration toward SMCs. To confirm this, we used the CRISPR/Cas9 genome-editing tool to knock out *Dcn* and *Bmp1* in WT and mutant cells and then assess their migration potential (Figures 4B, S3A, and S3B). Our data showed that BMP1 absence significantly mitigated migration, while absence of DCN enhanced it. We sought to investigate whether DCN and inhibition of BMP1 could ameliorate the increased migration observed in ApoE KO AdvSCA-1$^+$ cells *in vivo* (Figure 4C). When ApoE KO cells

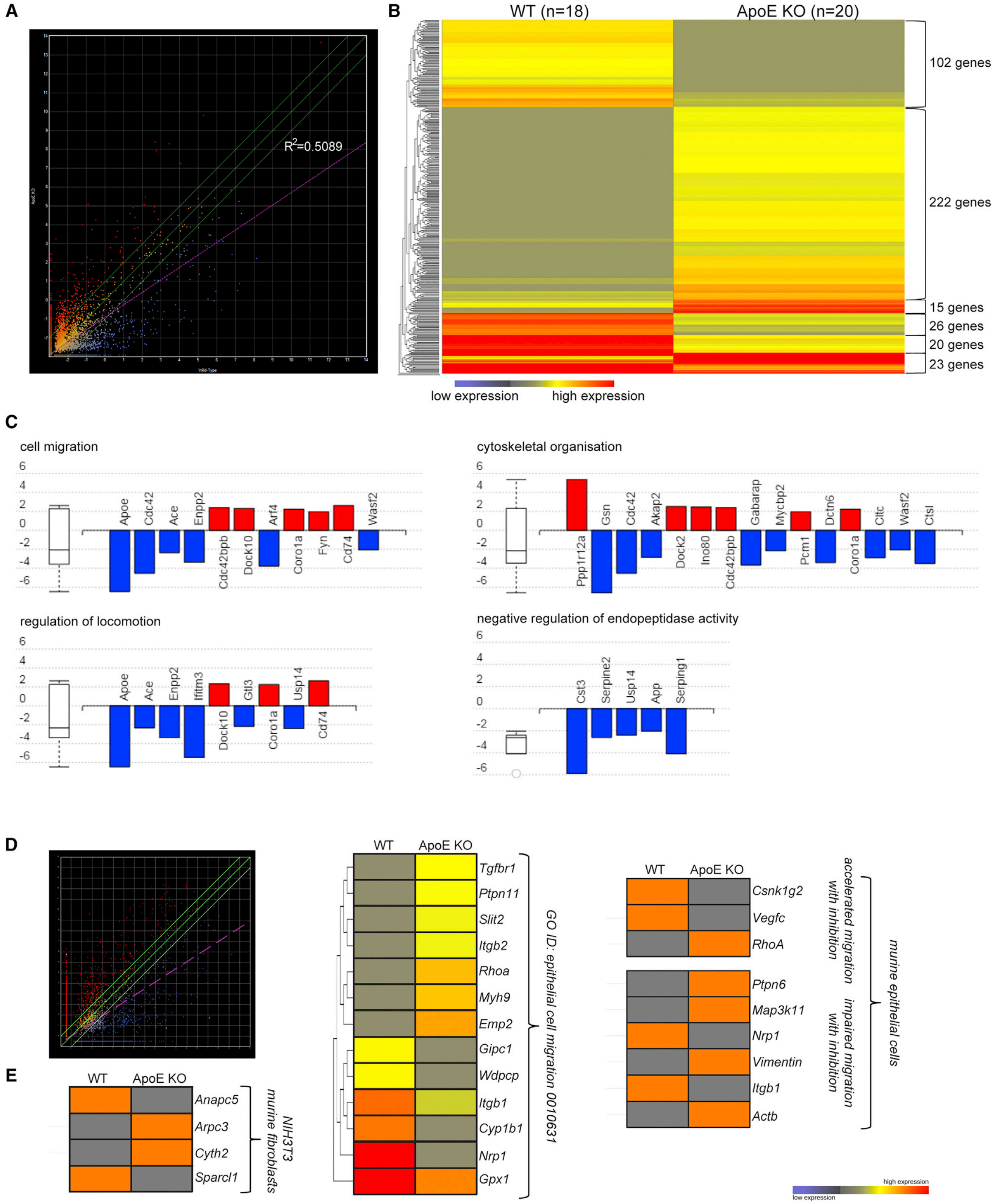

(legend on next page)

were treated with DCN and transplanted to the WT extravascular cell wall of the femoral artery, they were mostly retained in the adventitial and medial layers. BMP1 inhibitor treatment caused an even distribution of transplanted cells throughout the vascular wall. These results demonstrate that DCN and BMP1 levels influence AdvSCA-1$^+$ cell migration.

Based on these findings, we hypothesized that DCN and BMP1 may induce a synergistic effect on their most prominent target, collagen I (Ge and Greenspan, 2006; Keene et al., 2000). To test this we set up 24-hr and 48-hr cell cultures of semi-confluent WT and ApoE KO AdvSCA-1$^+$, from which supernatant was collected. Collagens I, II, and III and BMP1 protein levels were measured via ELISA in the presence of DCN and/or iBMP1 (Figures 4D and S3C). Protein levels in untreated cell cultures were in sync with gene expression levels (Figure 4E). Collagen expression was altered in the ApoE KO in comparison with WT. DCN had no effect on collagen I protein in the ApoE KO, while collagens II and III were affected in the WT, implying a conditioned collagen assembly dysregulation in the ApoE-deficient progenitors. Direct inhibition of BMP1 affected collagens I, II, and III of the WT progenitors only. Administration of both reagents adjusted collagen expression.

LDL-Bound and Free Cholesterol Are Potent Inducers of AdvSCA-1$^+$ Cell Migration, Inhibiting Their Differentiation

In ApoE KO mice hyperlipidemia contributes to atherosclerosis, with ApoE KO AdvSCA-1$^+$ cells displaying enhanced cell migration. Cholesterol has been documented to be present in the adventitia (Yatera et al., 2010). To interrogate the underlying mechanisms, we loaded WT and ApoE KO AdvSCA-1$^+$ with chol-MβD (water-soluble cholesterol) to evaluate the effect on progenitor cell migration. Transwell migration assays were performed, demonstrating that ApoE KO AdvSCA-1$^+$ progenitors displayed enhanced migration in response to chol-MβD compared with their WT counterparts (Figures 5A and 5B); DCN ameliorated this response, while iBMP1 administration showed contrasting results. Since ApoE KO AdvSCA-1$^+$ cell migration was influenced by cholesterol and low-density lipoprotein (LDL) administration (Figures S3D and S3E), we hypothesized that *in vitro* lipid loading would increase cell migration in wound-healing assays. Subsequent wound-healing assays also indicated a marked increase in the migration of chol-MβD-loaded cells when compared with untreated cells (Figure 5C). We confirmed these observations by documenting the migration of single AdvSCA-1$^+$ progenitors using time-lapse microscopy showing that chol-MβD-loaded progenitor cells possessed increased migrational speed and persistence (Figures 5D and 5E). Furthermore, immunofluorescence staining of the chol-MβD-loaded progenitors revealed F-actin reorganization, particularly nearer to the leading edge of the cells, as indicated by the yellow arrowheads in Figure 5F. Treatment with modified LDLs such as oxidized LDL (Ox-LDL) and acetylated LDL (Ac-LDL) also induced AdvSCA-1$^+$ cell migration, to the same extent as chol-MβD loading (Figure S3), confirming that the effects of water-soluble cholesterol administration are similar to those of natural cholesterol carriers. Additional data from experiments performed using cell counting, bromodeoxyuridine assays, and cyclin D1 expression profiling with real-time RT-PCR indicated that chol-MβD, Ox-LDL, and Ac-LDL inhibited progenitor cell proliferation (Figure S4). Together, these data suggest that cholesterol can induce SCA-1$^+$ cell increased migration.

Since AdvSCA-1$^+$ progenitors have a potential to differentiate to other cells that make up the vascular wall, we investigated whether chol-MβD or modified LDL loading could induce this machinery. Interestingly, these molecules did not induce differentiation into foam cells judged by their gene expression profile (Figure S5). Furthermore, the pre-treatment of progenitor cells with chol-MβD seemed to have resulted in inhibition of SMC-related (Figure 5G) and EC-related (Figure 5H) gene expression.

Figure 2. Differential Gene Expression Profile between WT and ApoE KO Adventitial SCA-1$^+$ Progenitors

(A) Scattergraph representing the relative single-cell expression of the 26,596 genes analyzed between WT (n = 18, y axis) and ApoE KO (n = 20, x axis) AdvSCA-1$^+$ cells (type II error rate 0.2, $r^2 = 0.52$).

(B) Heatmap representation of six major gene clusters showing the median statistically significant difference in relative gene expression between WT and ApoE KO AdvSCA-1$^+$ cells (Student's t test, $p < 0.01$, with a bidirectional 2 SD cutoff).

(C) GO biological processes revealed that key genes involved in regulation of cell migration, locomotion, cytoskeletal organization, and endopeptidase activity were downregulated 4-fold in the ApoE KO AdvSCA-1$^+$ cell population, in comparison with the WT (Erim pruning, >5 differentially expressed genes).

(D) Scattergraph representing the relative gene expression of AdvSCA-1$^+$ cells isolated, with high copy number of the *Sca-1* gene, analyzed between WT (n = 4, y axis) and ApoE KO (n = 5, x axis).

(E) Heatmap representation of median gene expression involved in NIH3T3 murine fibroblast cell and epithelial cell migration (adapted from Simpson et al., 2008) shows a difference in relative expression between WT and ApoE KO AdvSCA-1$^+$ cells (>4-fold gene expression difference, Student's t test, $p < 0.01$, with a bidirectional 2 SD cutoff).

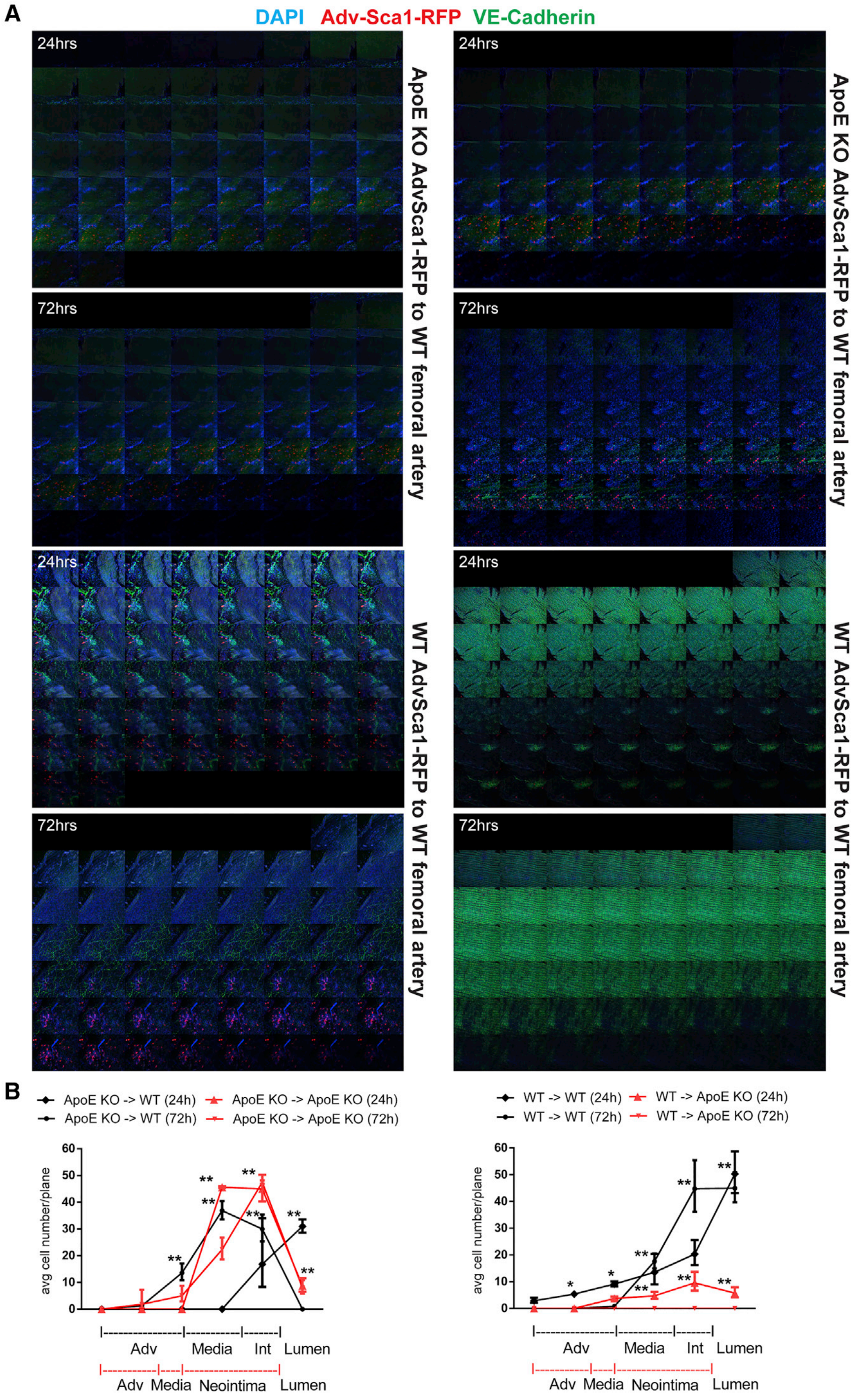

Figure 3. ApoE KO SCA-1⁺ Cells Show a Higher Migration toward the Inner Vascular Wall in Comparison with WT Counterparts

(A) Montage microphotographs. WT and ApoE KO AdvSCA-1⁺ cells were transduced with a nuclear RFP-inducing lentiviral construct. 1×10^6 cells in Matrigel plugs were transplanted to the outer side of the vascular wall of femoral arteries, in WT and ApoE KO 6-month-old animals. Cells were allowed to migrate for 24 and 72 hr prior to euthanizing the mice and assessing them with *en face* immunolabeling, using a VE-cadherin (green) antibody to mark the endothelial cell layer. DAPI is blue.

(B) Quantitative and spatial analysis of RFP⁺ cells migrating from the adventitia to the lumen from the outer vascular wall after 24 and 72 hr. n = 3 transplantation experiments, geometric means, error bars with 95% CI. $^*p < 0.05$, $^{**}p < 0.01$.

Cholesterol Promotes Migration toward Pro-inflammatory Cytokines

In parallel, we investigated the ability of AdvSCA-1⁺ progenitors to migrate toward a panel of pro-inflammatory cytokines, coinciding with altered gene expression of their prospective surface receptors, in comparison with WT AdvSCA-1⁺ cells (Figures S6A–S6C). Although interferon-γ and granulocyte macrophage colony-stimulating factor showed a high migration index, no difference was observed in combination with chol-MβD (data not shown). Instead, SCA-1⁺ cells were able to augment their migration and locomotion in response to tumor necrosis factor α (TNF-α)

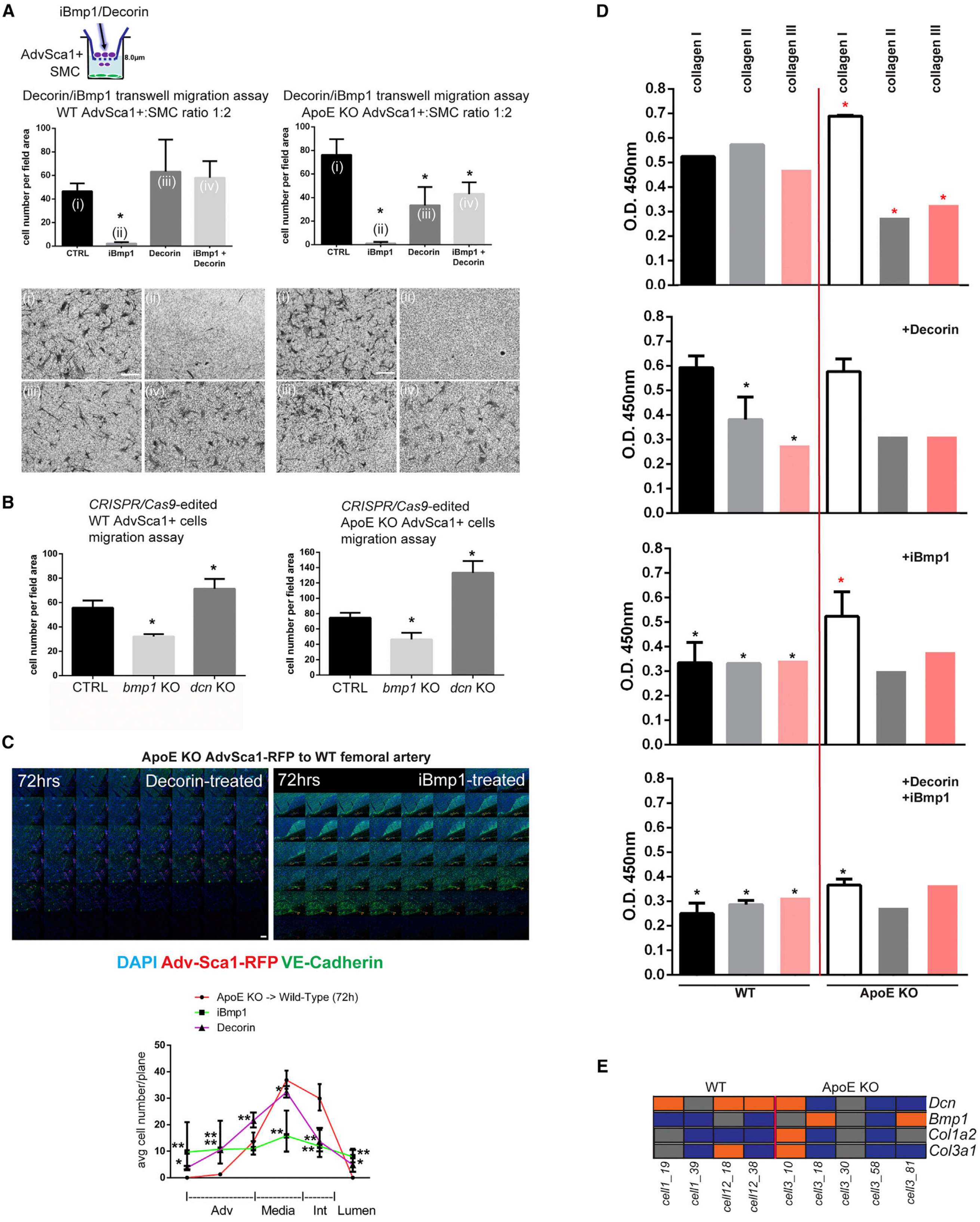

(legend on next page)

combined with chol-MβD, as shown using transwell and wound-healing assays (Figure 6A). These observations were confirmed in single-cell tracking experiments using time-lapse microscopy (Figures 6B and 6C). In a similar fashion, pre-treatments with Ox-LDL and Ac-LDL were also found to induce the migration of progenitor cells in response to TNF-α (Figure S6D).

MiRNA-29b Mediates Cholesterol-Induced SCA-1$^+$ Cell Migration

We aimed to elucidate *microRNA* expression that drives the chol-MβD-mediated chemotactic induction of AdvSCA-1$^+$ progenitors. We evaluated the potential changes of a panel of miRNA following loading with chol-MβD (Figure S7A). Chol-MβD markedly induced the expression of several miRNAs including miRNAs *29b-3p* and *32-5p*, while several miRNAs including *488-3p* were inhibited. *MicroRNAs 10a* and *10b* are known to act in a compensatory manner (for review see Feinberg and Moore, 2016). The induction of *miRNAs 29b-3p* and *32-5p* and inhibition of miRNA *488-3p* were subsequently confirmed using real-time PCR (Figures S7B–S7F).

MicroRNA 29b has been shown to participate in coronary artery disease (Chen et al., 2011). To test whether *miR-29b* is involved in the chol-MβD-mediated induction of AdvSCA-1$^+$ progenitor migration, we overexpressed miRNA 29b in the AdvSCA-1$^+$ progenitors by treating them with an mmu-miR-29b-3p mirVana miRNA mimic (Figure 6D). We observed that overexpression of *miR-29* significantly increased SCA-1$^+$ progenitor migration, both stochastically and in the presence of TNF-α. Subsequent experiments using an miRNA29 inhibitor showed a significant reduction in *miR-29* levels and attenuated the migration of chol-MβD-mediated cells (Figure 6E). It is thus tempting to postulate that cholesterol can enhance AdvSCA-1$^+$ progenitors' migratory response toward pro-inflammatory cytokines via *miR-29b* upregulation.

Cholesterol-Induced Migration Is Mediated by Sirtuin-1 Inhibition via MMP-9 Signaling

We next tested whether *Sirtuin-1* (*Sirt1*) played a role in the chol-MβD-mediated effects, since this molecule has been involved in endothelial progenitor migration (Li et al., 2015). Treatment of AdvSCA-1$^+$ progenitor cells with chol-MβD resulted in a suppression of *Sirt1* mRNA levels using RT-PCR (Figure 7A). Furthermore, the overexpression and inhibition of *miR-29* were observed to cause an inhibition and induction of S*irt1* expression, respectively. Additionally the inhibition of SIRT1, using a specific inhibitor (EX-527), markedly increased AdvSCA-1$^+$ progenitor cell migration, both randomly and toward TNF-α (Figure 7B).

Furthermore, treatment with chol-MβD or *miR-29* caused a marked increase in matrix metalloproteinase 9 (MMP-9) expression in AdvSCA-1$^+$ progenitor cells (Figures 7C and 7D). Consistently, the inhibition of *miR-29* and SIRT-1 in progenitor cells, in the presence of cholesterol, caused a reduction and increase in *Mmp-9* expression, respectively (Figure 7E). We also observed that treatment with an MMP-9 inhibitor regulated chol-MβD-mediated migration, even in the presence of TNF-α (Figure 7F). Taken together, these data suggest that chol-MβD can induce *MiR-29*, which in turn suppresses *Sirt1* and upregulates MMP-9 levels to induce AdvSCA-1$^+$ progenitor cell migration.

DISCUSSION

Previous studies have demonstrated that adventitial fibroblasts migrate from the adventitia to the neointima upon balloon injury in rats (Li et al., 2000), while AdvSCA-1$^+$ progenitors potentially participate in neointimal aggravation of vein grafts (Hu et al., 2004). Studies from our laboratory have demonstrated their potential role in native atherosclerosis (Mayr et al., 2008). A recent report using lineage-tracing analysis reported that more than 50% of neointimal

Figure 4. Intervention Targets for Adventitial ApoE KO SCA-1$^+$ Inhibition of Cell Migration toward the Media

(A) 1 × 10^5 WT or ApoE KO AdvSCA-1$^+$ cells were loaded with 10 μg/mL iBMP1 and/or DCN in 0.2% fetal bovine serum (FBS) and allowed to pass through 8.0-μm transwells seeded with 1 × 10^5 WT vSMCs for 24 hr. n = 3 independent experiments, geometric means, error bars with 95% CI. *p < 0.05. Scale bars, 50 μm.

(B) CRISPR/Cas9-edited AdvSCA-1$^+$ cells were induced to migrate in 0.2% FBS passing through 8.0-μm transwells for 24 hr. n = 3 independent experiments. Error bars are SEM. *p = 0.05 compared with control.

(C) Montage microphotographs. WT and ApoE KO AdvSCA-1$^+$ cells were transduced with a nuclear RFP-inducing lentiviral construct. Matrigel plugs containing 1 × 10^6 cells were transplanted to the outer side of the vascular wall, in WT and ApoE KO 6-month-old animals (n = 3 transplantation experiments). Cells were allowed to migrate for 72 hr prior to euthanizing the mice and assessing them with *en face* immunolabeling, using a VE-cadherin antibody to mark the EC layer. DAPI is blue. Quantitative and spatial analysis of RFP$^+$ cells migrating from the adventitia to the lumen from the outer vascular wall. *p < 0.05 and **p < 0.01 compared with the untreated group.

(D) 3 × 10^4 WT or ApoE KO AdvSCA-1$^+$ cells were seeded onto 12-well plates and treated with iBMP1 and/or DCN. Supernatant was collected after 24 hr. ELISA assays were performed against pro-collagens I and II, and collagen III. n = 3 independent experiments. *p < 0.05 compared with untreated control. *p < 0.05, WT compared with ApoE KO.

(E) Heatmap of *Sca-1*^{high} cells differentially expressing *Dcn*, *Bmp1*, *Col1a2*, and *Col3a1*.

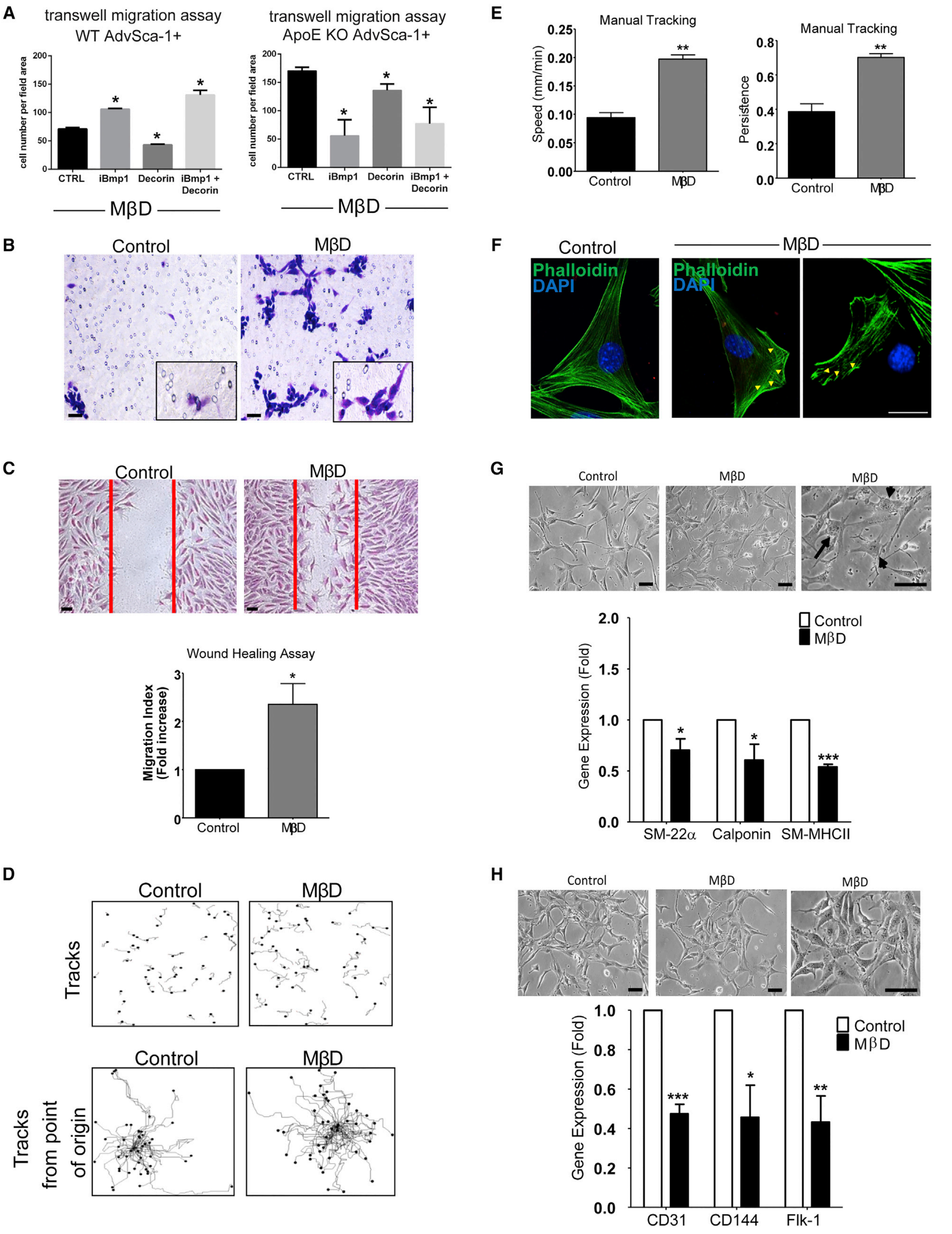

(legend on next page)

SMCs derived from adventitial stem cells (Kramann et al., 2016). We provide definitive evidence that atherosclerosis-prone-derived AdvSCA-1$^+$ progenitors have a different migration pattern to AdvSCA-1$^+$ deriving from atherosclerosis-protected mice. In the present study, we show that hyperlipidemia triggers changes in gene expression in AdvSCA-1$^+$ cells, promoting ECM remodeling and cell migration. Collagen ratios are altered in ApoE KO AdvSCA-1$^+$ progenitors. Increase in collagen I/III ratio due to fatty acid excess has been shown to cause cardiac "stiffness" (Beam et al., 2015). A metabolomics analysis revealed a significant increase in fatty acid metabolites in ApoE KO AdvSCA-1$^+$ progenitors (our unpublished data). The presence of pro-collagen II is an indication of the unavoidable discrepancies that appear between *in situ* and *in vitro* experimental systems. It is also quite possible that since SCA-1$^+$ cells were allowed to migrate for 24 hr, in the absence of leukemia inhibitory factor, they also began producing collagen II due to differentiation.

There are several implications that derive from this study. First, *in situ* single-cell gene sequencing indicates that the majority of AdvSCA-1$^+$ cells are local resident progenitor/precursor cells, since a very small proportion of AdvSCA-1$^+$ displayed bone marrow cell markers. Second, water-soluble cholesterol and modified LDLs are able to alter the gene expression and function of AdvSCA-1$^+$ cells related to migration. Third, by combining single-cell differential gene expression analysis with established *in vitro* and *in vivo* cell migration systems, we reveal the use of potential molecules that can regulate AdvSCA-1$^+$ migration.

We specifically link DCN and BMP1 to extracellular collagen remodeling in cholesterol-loaded AdvSCA-1$^+$ cells; in contrast, *microRNA 29b* induces migration, possibly via *Sirt1* (Schematic Diagram S1). DCN is a small leucine-rich proteoglycan that plays important roles in atherosclerosis (Singla et al., 2011). In Fischer et al. (2000), reported an amelioration of intimal formation upon balloon injury in rats after *Dcn* gene overexpression. Although its expression is primarily observed in the *tunica adventitia*, it affects SMC response to growth factors (Fischer et al., 2001; Nili et al., 2003; Ueda et al., 2015). Decorin overexpression reduces atherosclerosis in ApoE KO mice (Al Haj Zen et al., 2006). Here, we show that in the same mouse model there is a shift of DCN expression/secretion from the adventitia to the medial layer. Whether this is a compensatory mechanism required for neointima stabilization remains to be elucidated. Studies performed in the skin of ApoE and DCN double KOs showed an overall reduced DCN expression, similar to our findings in the aorta (Hiebert et al., 2011). In diabetic nephropathy, DCN acts in a protective manner, by reducing ECM accumulation (Merline et al., 2009); its absence attracts more mononuclear cells to the sites of insult, while in ocular fibroblasts a combination of suramin and DCN specifically inhibits collagen production (Mietz et al., 1997). A recent study in abdominal aortic aneurysm showed that DCN stabilizes the ECM and inflammation, preventing induced pathology (Ueda et al., 2015). The authors cautiously note the dual role of this molecule, promoting macrophage-induced pathology while ameliorating a vSMC-induced one. It is therefore highly likely that DCN's role in atherosclerosis is cell specific, as shown recently, in the human heart (Barallobre-Barreiro et al., 2016).

DCN has been linked to collagen synthesis and BMP1 function (Keene et al., 2000; von Marschall and Fisher, 2010). We found that *Bmp1* is overexpressed in the mutant, while *collagen I* and *collagen III* gene expression was altered. The function of BMP1 on transforming growth factor β (TGF-β) cleavage has been reported (Ge and Greenspan, 2006), while DCN also affects TGF-β's function (Ferdous et al., 2007). BMP1 is a potent pro-collagen cleavage protease that is involved in atherosclerosis (Derwall et al., 2012); in fact, its reduction decreases vascular wall calcification. We hypothesized that these molecules act synergistically in collagen production in AdvSCA-1$^+$ cells. The addition of iBMP1 nearly abolished AdvSCA-1$^+$ migration, with minimal cell toxicity, while DCN addition affected only ApoE KO SCA-1$^+$ cells but not WT SCA-1$^+$ cells. Thus,

Figure 5. Cholesterol Induces SCA-1$^+$ Cell Migration and Inhibits Differentiation

(A and B) 1 × 10^5 WT or ApoE KO AdvSCA-1$^+$ cells were loaded with 20 μg/mL cholesterol along with 10 μg/mL iBMP1 and/or DCN in serum-free media and allowed to pass through 8.0-μm transwells in the presence of 0.2% FBS for 24 hr.

(C) Migration of SCA-1$^+$ progenitor cells was evaluated using a wound-healing assay. Migration index for both assays was defined as the mean number of progenitors counted per 5 random fields of view at 20×.

(D) Vascular progenitor cells were seeded at 1 × 10^4 cells per well of 6-well plate and subjected to 48 hr of 20 μg/mL chol-MβD loading.

(E) Single-cell migration was documented using time-lapse microscopy, and quantified for speed and persistence.

(F) Changes in F-actin distribution was analyzed (yellow arrowheads) using immunofluorescence staining with phalloidin (Alexa 488; green) and DAPI (blue).

(G and H) The effect of cholesterol on smooth muscle cell (G) and endothelial cell (H) differentiation was observed using phase-contrast light microscopy. Black arrows represent a change in cell morphology. Total RNA was harvested for analysis of smooth muscle cell gene expression using real-time RT-PCR. Graphs are shown as mean ± SEM of three independent experiments (n = 3).

*$p < 0.1$, **$p < 0.05$, ***$p < 0.01$ compared with untreated control. Scale bars, 20 μm.

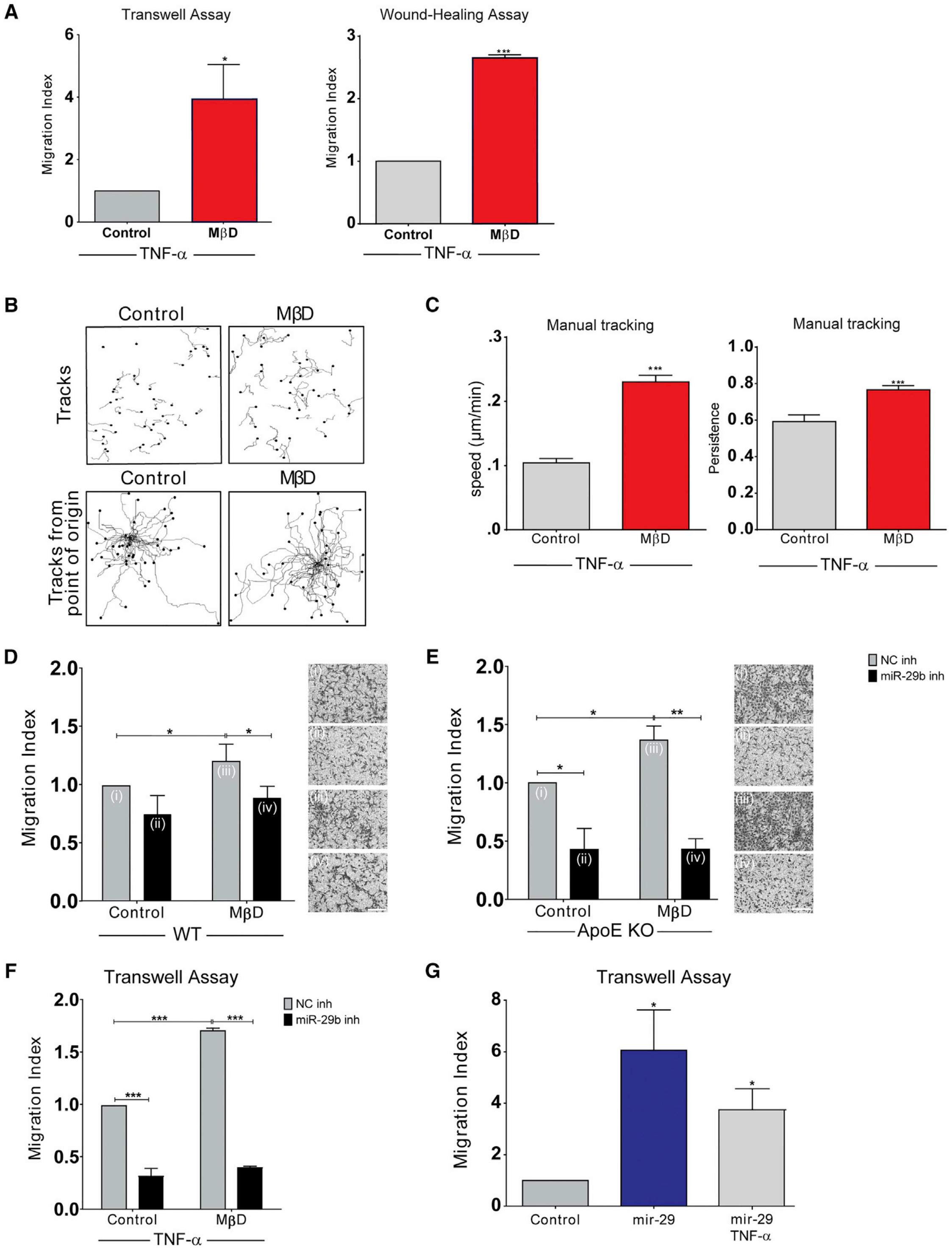

Figure 6. Cholesterol Can Induce SCA-1+ Cell Migration toward Pro-inflammatory Cytokines, along with *MicroRNA 29b*

(A) Migration of vascular progenitor cells, either untreated or loaded with 20 μg/mL chol-MβD for 48 hr, toward 10 ng/mL TNF-α was evaluated using transwell and wound-healing assays.

(B and C) SCA-1+ cells were seeded at 5×10^3 cells per well of 6-well plate and subjected to 48 hr of 20 μg/mL chol-MβD loading and 10 ng/mL TNF-α. Single-cell migration was documented using time-lapse microscopy and quantified for speed and persistence.

(legend continued on next page)

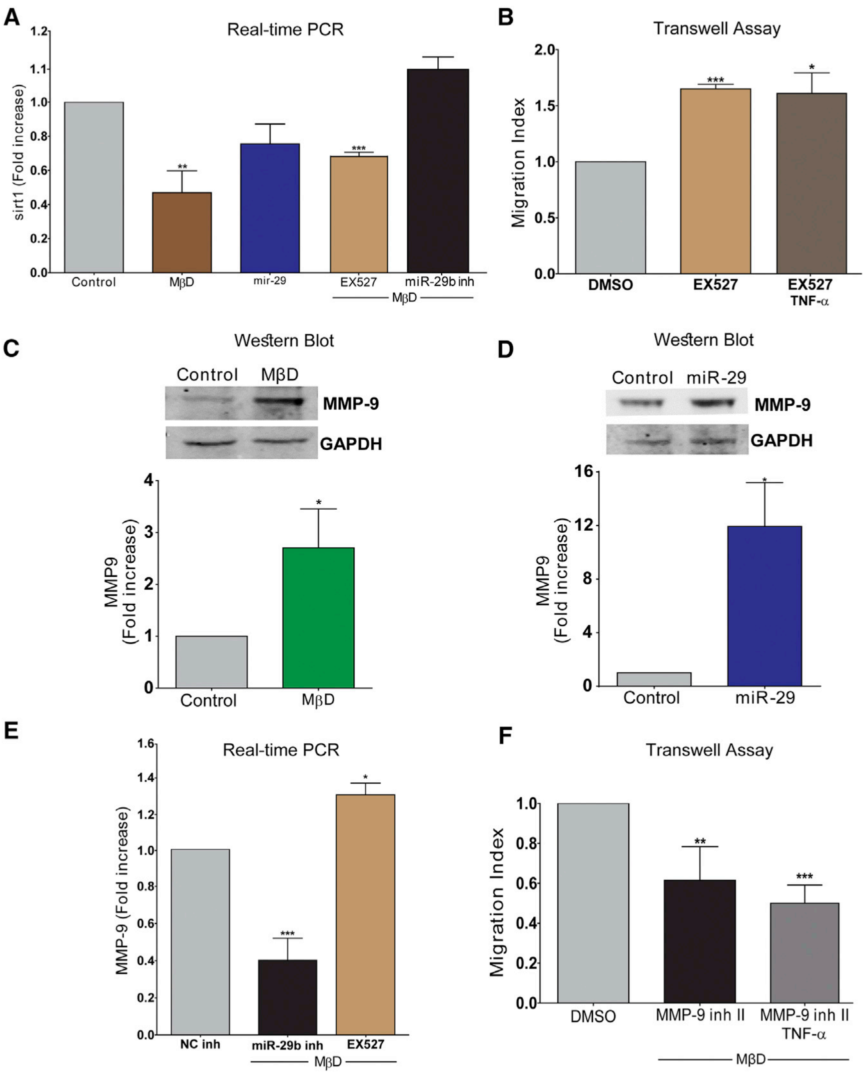

Figure 7. *MicroRNA 29b* Affects Cholesterol-Loaded SCA-1$^+$ Cell Migration via Sirtuin-1/MMP-9

(A) SCA-1$^+$ cells were loaded with 20 μg/mL chol-MβD, overexpressed with *miRNA-29*, pre-treated with 50 μM EX-527 (SIRT-1 inhibitor), or treated with 20 μg/mL chol-MβD and a hsa-miR-29b-3p ID: MH10103 mirVana miRNA inhibitor. A mirVana miRNA inhibitor Negative Control #1 or DMSO were used as controls where appropriate. Cell lysates were harvested and subjected to real-time PCR for the detection of *Sirt-1* mRNA expression.

(B) Migration of SCA-1$^+$ cell toward serum-free media, with TNF-α (10 ng/mL) in the presence of 50 μM EX-527 evaluated using 8.0-μm transwell assays.

(C and D) Cell lysates were collected and western blotting was performed for MMP-9 detection.

(E) SCA-1$^+$ cells were pre-loaded with chol-MβD and treated with a hsa-miR-29b-3p ID: MH10103 *mir*Vana miRNA inhibitor or 50 μM EX-527. Cell lysates were harvested and subjected to real-time RT-PCR.

(F) The effect of MMP-9 inhibition (pre-treatment with 20 μM MMP-9 inhibitor II) on the migration of chol-MβD loaded cells toward serum-free media or TNF-α (10 ng/mL) was evaluated using 8.0-μm transwell assays.

Data in graphs are shown as mean ± SEM of three independent experiments. $^*p < 0.1$, $^{**}p < 0.05$, $^{***}p < 0.01$ compared with untreated control.

addition of DCN or DCN/iBMP1 fine-tunes ApoE KO AdvSCA-1$^+$ cells into a perhaps more WT AdvSCA-1$^+$ migratory (atheroprotective) behavior.

The search for cues important for AdvSCA-1$^+$ migration led us to investigate a cohort of miRNAs in relation to this. Lipid loading showed that ApoE KO progenitors were indeed more prone to migrate toward SMCs, possibly due to their altered ECM gene expression profile. Recently, we reported that AdvSCA-1$^+$ cells respond to vSMC-derived chemokine cues contributing to neointima formation (Yu et al., 2016). Since 1998, when the ApoE KO mouse model was employed for studies of atherosclerosis and hyperlipidemia (Hofker et al., 1998), it has not been shown how LDLs contribute to specific effects on AdvSCA-1$^+$ cells. It has been recently documented that DNA hypomethylation is important for the onset of the disease, along with lifestyle factors (Jiang et al., 2012). Thus, miRNA expression levels may have a strong influence in controlling the disease. Our experiments show that *miR-29b* elicits a response to the migratory potential of lipid-loaded AdvSCA-1$^+$,

(D and E) Migration of SCA-1$^+$ cell toward serum-free media, loaded with 20 μg/mL chol-MβD and in the presence of hsa-miR-29b-3p miRNA inhibitor. Scale bars, 100 μm.

(F and G) Migration of SCA-1$^+$ cell toward serum-free media, or loaded with 20 μg/mL chol-MβD and TNF-α (10 ng/mL) in the presence of *hsa-miR-29b-3p* miRNA and its inhibitor.

Data in graphs are shown as mean ± SEM of three independent experiments. $^*p < 0.1$, $^{**}p < 0.05$, $^{***}p < 0.01$ compared with untreated control.

possibly via *Sirt1* and MMP-9, with the latter documented to play a role in atherosclerosis (Chen et al., 2008; Gough et al., 2006; Pleva et al., 2015), while the former is a molecule now shown to be involved in AdvSCA-1$^+$ progenitors. Of note, it has been reported that human *MiR-29b* has been implicated in ECM remodeling including collagen regulation (Kriegel et al., 2012), while its dysregulation in murine cardiac fibroblasts upon post-myocardial infraction led to decreased collagen formation and fibrosis (van Rooij et al., 2008). It was also interesting how pro-inflammatory cytokines such as TNF-α affected this pathway, meaning that innate immunity may play a crucial role.

Atherosclerosis is a chronic inflammatory disease characterized by lipoprotein accumulation that carries cholesterol inside the arteries. Accumulation of LDL and cholesterol leads to aberrant activation of vSMCs and macrophages within the vessel wall (Xu et al., 2015). Modified LDLs have been also known to increase foam cell formation, influencing lesion development (Cox et al., 2007). Although these molecules have been implicated in pro- and anti-atherosclerotic responses, their effect on Sca-1$^+$ progenitors has not been investigated. Loading of AdvSCA-1$^+$ with MβD had an immediate effect on their migration potential, causing cytoskeletal rearrangements characteristic of increased locomotion. Ox- and Ac-LDL administration had a modest effect on migration, along with affecting SCA-1$^+$ cell proliferation and viability; in line with these results, mRNA levels of known scavenger receptors that bind modified LDL molecules such as *Cd63*, *Cd68*, *Lox-1*, *Msra*, and others remained unchanged. We thus attempted to identify the mechanism by which chol-MβD induces SCA-1$^+$ ECM modifications that induce migration. The role of MMP-9 in this process has been documented in macrophage function and arterial enlargement during atherosclerosis (Chen et al., 2008; Gough et al., 2006; Karwowski et al., 1999; Tronc et al., 2000). While this paper was being written, it was reported that *Mmp-9-deficient* mice had an abnormal cholesterol metabolism, leading to atherosclerosis (Hernandez-Anzaldo et al., 2016). Here, we show that AdvSCA-1$^+$ MMP-9 levels were elevated significantly upon exposure to chol-MβD, underscoring the potential ability of AdvSCA-1$^+$ to alter the ECM. Based on this, we investigated whether *Sirt1* inhibition, whose expression has been reported to inhibit atherosclerosis in vSMCs (Gorenne et al., 2013), also had an effect on SCA-1$^+$ progenitor migration. Indeed, by using a specific inhibitor or loading with chol-MβD, progenitors' migration index was increased in a linearly inverted fashion. *MicroRNA 29b-3p* expression levels affected chol-MβD-loaded AdvSCA-1$^+$. Two of the mechanisms of action were via a modest *Sirt1* change and a massive MMP-9 upregulation. To our knowledge, no report has shown to date that an miRNA can alter the lipid-loaded migratory effect of AdvSCA-1$^+$ progenitors.

In summary, our results provide some clarity of the migratory mechanisms of resident AdvSCA-1$^+$ progenitors. (1) Hyperlipidemic mice possess more HSC-derived progenitors in the adventitia, in concert with previous studies (Hu et al., 2002a, 2002b); (2) AdvSCA-1$^+$ cells from ApoE KO mice are undergoing EMT, which could be an early sign of vascular stiffening; (3) ApoE KO AdvSCA-1$^+$ cells have a higher migratory potential than their WT counterparts; finally, (4) it seems that the AdvSCA-1$^+$ ApoE KO cells show an altered ECM, possibly due to dysfunctional lipid loading, which may trigger or augment an unwanted para-inflammatory immune response, a characteristic of age-related disease (Chen et al., 2012; Xu et al., 2009). Therefore, these results could provide a potential strategy for the treatment of atherosclerosis by directing adventitial cell migration.

EXPERIMENTAL PROCEDURES

Animals

All animal procedures were performed according to the protocol reviewed by the Institution Ethics Committee and approved by the UK Home Office. All mice used were on identical genetic background.

Single-Cell Gene Expression Analysis

Mouse adventitial cells were collected and pooled from six aortas of 6-month-old mice, for each genotype. SCA-1$^+$ cells were isolated from primary cultured cells upon reaching 90% confluence using a microbeads kit (Miltenyi Biotec, Bergisch Gladbach, Germany) containing anti-SCA-1 immunomagnetic microbeads and a magnetic cell-sorting system column. Cells were further analyzed using the Fluidigm C1 machine and workflow following the manufacturer's protocol.

AdvSCA-1$^+$ Progenitor Cell Culture and Differentiation

Mouse adult progenitor cells were derived from an outgrowth of adventitial tissues of vessel grafts as previously described (Hu et al., 2004; Tsai et al., 2012).

Cell Transplantations

For the *in vivo* cell transplantations, 1×10^6 cells were immersed in Matrigel for 30 min on ice prior to transplanting them to the extra-adventitial space of the femoral artery of 6-month-old mice. Decorin and iBMP1 were administered along with the Matrigel, where appropriate. Mice were then euthanized 24 and 72 hr later, and femoral arteries were collected and fixed on 4% paraformaldehyde for 15 min prior to further analysis.

Exogenous Proteins, Antagonists, Cholesterol, and Modified LDL Loading

For a detailed description, see Supplemental Experimental Procedures.

Transwell Chemotaxis and Scratch-Wound Assays

For a detailed description, see Supplemental Experimental Procedures.

MicroRNA Transient Transfection

Manipulation of miRNA levels in vascular progenitor cells (cultured to 60%–70% confluence) was carried out using an *mmu-miR-29b-3p* mouse *mir*Vana miRNA mimic (5 μM), according to the manufacturer's protocol.

SUPPLEMENTAL INFORMATION

Supplemental Information includes Supplemental Experimental Procedures, seven figures, and one schematic diagram and can be found with this article online at http://dx.doi.org/10.1016/j.stemcr.2017.06.011.

AUTHOR CONTRIBUTIONS

I.K. designed and performed experiments, analyzed data, and wrote the majority of the paper; M.M.W. designed and performed experiments, analyzed data, and wrote part of the paper; C.M.F.P. performed experiments; Y.X. performed transplantations; B.Y. and Y.H. provided tissues; D.T.W. assisted with cell tracking; W.N.N. helped in metabolomics and cell surface analysis; A.l.B. provided reagents; Z.N. helped with experiments; C.Z., X.R., L.Z., and E.K. provided help with the study design; Q.X. conceived the major part of the study; L.Z. and Q.X. designed the whole study and obtained funding.

ACKNOWLEDGMENTS

This work was supported by the British Heart Foundation (RG/14/6/31144 and RM/13/2/30158) and the National Natural Science Foundation of China (91639302, 91339102, and 91539103).

Received: January 27, 2017
Revised: June 23, 2017
Accepted: June 23, 2017
Published: July 27, 2017

REFERENCES

Al Haj Zen, A., Caligiuri, G., Sainz, J., Lemitre, M., Demerens, C., and Lafont, A. (2006). Decorin overexpression reduces atherosclerosis development in apolipoprotein E-deficient mice. Atherosclerosis *187*, 31–39.

Alessandri, G., Girelli, M., Taccagni, G., Colombo, A., Nicosia, R., Caruso, A., Baronio, M., Pagano, S., Cova, L., and Parati, E. (2001). Human vasculogenesis *ex vivo*: embryonal aorta as a tool for isolation of endothelial cell progenitors. Lab. Invest. *81*, 875–885.

Barallobre-Barreiro, J., Gupta, S.K., Zoccarato, A., Kitazume-Taneike, R., Fava, M., Yin, X., Werner, T., Hirt, M.N., Zampetaki, A., Viviano, A., et al. (2016). Glycoproteomics reveals decorin peptides with anti-myostatin activity in human atrial fibrillation. Circulation *134*, 817–832.

Beam, J., Botta, A., Ye, J., Soliman, H., Matier, B.J., Forrest, M., MacLeod, K.M., and Ghosh, S. (2015). Excess linoleic acid increases collagen I/III ratio and "stiffens" the heart muscle following high fat diets. J. Biol. Chem. *290*, 23371–23384.

Bobryshev, Y.V., Orekhov, A.N., and Chistiakov, D.A. (2015). Vascular stem/progenitor cells: current status of the problem. Cell Tissue Res. *362*, 1–7.

Campagnolo, P., Tsai, T.N., Hong, X., Kirton, J.P., So, P.W., Margariti, A., Di Bernardini, E., Wong, M.M., Hu, Y., Stevens, M.M., et al. (2015). c-Kit+ progenitors generate vascular cells for tissue-engineered grafts through modulation of the Wnt/Klf4 pathway. Biomaterials *60*, 53–61.

Chen, K.C., Wang, Y.S., Hu, C.Y., Chang, W.C., Liao, Y.C., Dai, C.Y., and Juo, S.H. (2011). OxLDL up-regulates microRNA-29b, leading to epigenetic modifications of MMP-2/MMP-9 genes: a novel mechanism for cardiovascular diseases. FASEB J. *25*, 1718–1728.

Chen, M., Zhao, J., Luo, C., Pandi, S.P., Penalva, R.G., Fitzgerald, D.C., and Xu, H. (2012). Para-inflammation-mediated retinal recruitment of bone marrow-derived myeloid cells following whole-body irradiation is CCL2 dependent. Glia *60*, 833–842.

Chen, Y., Aratani, Y., Osawa, T., Fukuyama, N., Tsuji, C., and Nakazawa, H. (2008). Activation of inducible nitric oxide synthase increases MMP-2 and MMP-9 levels in ApoE-knockout mice. Tokai J. Exp. Clin. Med. *33*, 28–34.

Chen, Y., Wong, M.M., Campagnolo, P., Simpson, R., Winkler, B., Margariti, A., Hu, Y., and Xu, Q. (2013). Adventitial stem cells in vein grafts display multilineage potential that contributes to neointimal formation. Arterioscler Thromb. Vasc. Biol. *33*, 1844–1851.

Cox, B.E., Griffin, E.E., Ullery, J.C., and Jerome, W.G. (2007). Effects of cellular cholesterol loading on macrophage foam cell lysosome acidification. J. Lipid Res. *48*, 1012–1021.

Derwall, M., Malhotra, R., Lai, C.S., Beppu, Y., Aikawa, E., Seehra, J.S., Zapol, W.M., Bloch, K.D., and Yu, P.B. (2012). Inhibition of bone morphogenetic protein signaling reduces vascular calcification and atherosclerosis. Arterioscler Thromb. Vasc. Biol. *32*, 613–622.

Feinberg, M.W., and Moore, K.J. (2016). MicroRNA regulation of atherosclerosis. Circ. Res. *118*, 703–720.

Ferdous, Z., Wei, V.M., Iozzo, R., Hook, M., and Grande-Allen, K.J. (2007). Decorin-transforming growth factor-interaction regulates matrix organization and mechanical characteristics of three-dimensional collagen matrices. J. Biol. Chem. *282*, 35887–35898.

Ferreira, L.S., Gerecht, S., Shieh, H.F., Watson, N., Rupnick, M.A., Dallabrida, S.M., Vunjak-Novakovic, G., and Langer, R. (2007). Vascular progenitor cells isolated from human embryonic stem cells give rise to endothelial and smooth muscle like cells and form vascular networks *in vivo*. Circ. Res. *101*, 286–294.

Fischer, J.W., Kinsella, M.G., Clowes, M.M., Lara, S., Clowes, A.W., and Wight, T.N. (2000). Local expression of bovine decorin by cell-mediated gene transfer reduces neointimal formation after balloon injury in rats. Circ. Res. *86*, 676–683.

Fischer, J.W., Kinsella, M.G., Levkau, B., Clowes, A.W., and Wight, T.N. (2001). Retroviral overexpression of decorin differentially affects the response of arterial smooth muscle cells to growth factors. Arterioscler Thromb. Vasc. Biol. *21*, 777–784.

Ge, G., and Greenspan, D.S. (2006). BMP1 controls TGFbeta1 activation via cleavage of latent TGFbeta-binding protein. J. Cell Biol. *175*, 111–120.

Gorenne, I., Kumar, S., Gray, K., Figg, N., Yu, H., Mercer, J., and Bennett, M. (2013). Vascular smooth muscle cell sirtuin 1 protects against DNA damage and inhibits atherosclerosis. Circulation *127*, 386–396.

Gough, P.J., Gomez, I.G., Wille, P.T., and Raines, E.W. (2006). Macrophage expression of active MMP-9 induces acute plaque disruption in apoE-deficient mice. J. Clin. Invest. *116*, 59–69.

Hernandez-Anzaldo, S., Brglez, V., Hemmeryckx, B., Leung, D., Filep, J.G., Vance, J.E., Vance, D.E., Kassiri, Z., Lijnen, R.H., Lambeau, G., et al. (2016). Novel role for matrix metalloproteinase 9 in modulation of cholesterol metabolism. J. Am. Heart Assoc. *5*, e004228.

Hiebert, P.R., Boivin, W.A., Abraham, T., Pazooki, S., Zhao, H., and Granville, D.J. (2011). Granzyme B contributes to extracellular matrix remodeling and skin aging in apolipoprotein E knockout mice. Exp. Gerontol. *46*, 489–499.

Hill, K.L., Obrtlikova, P., Alvarez, D.F., King, J.A., Keirstead, S.A., Allred, J.R., and Kaufman, D.S. (2010). Human embryonic stem cell-derived vascular progenitor cells capable of endothelial and smooth muscle cell function. Exp. Hematol. *38*, 246–257.e1.

Hofker, M.H., van Vlijmen, B.J., and Havekes, L.M. (1998). Transgenic mouse models to study the role of APOE in hyperlipidemia and atherosclerosis. Atherosclerosis *137*, 1–11.

Hu, Y., Davison, F., Ludewig, B., Erdel, M., Mayr, M., Url, M., Dietrich, H., and Xu, Q. (2002a). Smooth muscle cells in transplant atherosclerotic lesions are originated from recipients, but not bone marrow progenitor cells. Circulation *106*, 1834–1839.

Hu, Y., Mayr, M., Metzler, B., Erdel, M., Davison, F., and Xu, Q. (2002b). Both donor and recipient origins of smooth muscle cells in vein graft atherosclerotic lesions. Circ. Res. *91*, e13–20.

Hu, Y., Zhang, Z., Torsney, E., Afzal, A.R., Davison, F., Metzler, B., and Xu, Q. (2004). Abundant progenitor cells in the adventitia contribute to atherosclerosis of vein grafts in ApoE-deficient mice. J. Clin. Invest. *113*, 1258–1265.

Invernici, G., Madeddu, P., Emanueli, C., Parati, E.A., and Alessandri, G. (2008). Human fetal aorta-derived vascular progenitor cells: identification and potential application in ischemic diseases. Cytotechnology *58*, 43–47.

Jiang, Y., Zhang, H., Sun, T., Wang, J., Sun, W., Gong, H., Yang, B., Shi, Y., and Wei, J. (2012). The comprehensive effects of hyperlipidemia and hyperhomocysteinemia on pathogenesis of atherosclerosis and DNA hypomethylation in ApoE$^{-/-}$ mice. Acta Biochim. Biophys. Sin (Shanghai) *44*, 866–875.

Karwowski, J.K., Markezich, A., Whitson, J., Abbruzzese, T.A., Zarins, C.K., and Dalman, R.L. (1999). Dose-dependent limitation of arterial enlargement by the matrix metalloproteinase inhibitor RS-113,456. J. Surg. Res. *87*, 122–129.

Keene, D.R., San Antonio, J.D., Mayne, R., McQuillan, D.J., Sarris, G., Santoro, S.A., and Iozzo, R.V. (2000). Decorin binds near the C terminus of type I collagen. J. Biol. Chem. *275*, 21801–21804.

Kramann, R., Goettsch, C., Wongboonsin, J., Iwata, H., Schneider, R.K., Kuppe, C., Kaesler, N., Chang-Panesso, M., Machado, F.G., Gratwohl, S., et al. (2016). Adventitial MSC-like cells are progenitors of vascular smooth muscle cells and drive vascular calcification in chronic kidney disease. Cell Stem Cell *19*, 628–642.

Kriegel, A.J., Liu, Y., Fang, Y., Ding, X., and Liang, M. (2012). The miR-29 family: genomics, cell biology, and relevance to renal and cardiovascular injury. Physiol. Genomics *44*, 237–244.

Li, G., Chen, S.J., Oparil, S., Chen, Y.F., and Thompson, J.A. (2000). Direct *in vivo* evidence demonstrating neointimal migration of adventitial fibroblasts after balloon injury of rat carotid arteries. Circulation *101*, 1362–1365.

Li, W., Du, D., Wang, H., Liu, Y., Lai, X., Jiang, F., Chen, D., Zhang, Y., Zong, J., and Li, Y. (2015). Silent information regulator 1 (SIRT1) promotes the migration and proliferation of endothelial progenitor cells through the PI3K/Akt/eNOS signaling pathway. Int. J. Clin. Exp. Pathol. *8*, 2274–2287.

Lusis, A.J. (2000). Atherosclerosis. Nature *407*, 233–241.

Mayr, M., Zampetaki, A., Sidibe, A., Mayr, U., Yin, X., De Souza, A.I., Chung, Y.L., Madhu, B., Quax, P.H., Hu, Y., et al. (2008). Proteomic and metabolomic analysis of smooth muscle cells derived from the arterial media and adventitial progenitors of apolipoprotein E-deficient mice. Circ. Res. *102*, 1046–1056.

Merline, R., Lazaroski, S., Babelova, A., Tsalastra-Greul, W., Pfeilschifter, J., Schluter, K.D., Gunther, A., Iozzo, R.V., Schaefer, R.M., and Schaefer, L. (2009). Decorin deficiency in diabetic mice: aggravation of nephropathy due to overexpression of profibrotic factors, enhanced apoptosis and mononuclear cell infiltration. J. Physiol. Pharmacol. *60* (*Suppl 4*), 5–13.

Mietz, H., Chevez-Barrios, P., Lieberman, M.W., Wendt, M., Gross, R., and Basinger, S.F. (1997). Decorin and suramin inhibit ocular fibroblast collagen production. Graefes Arch. Clin. Exp. Ophthalmol. *235*, 399–403.

Nili, N., Cheema, A.N., Giordano, F.J., Barolet, A.W., Babaei, S., Hickey, R., Eskandarian, M.R., Smeets, M., Butany, J., Pasterkamp, G., et al. (2003). Decorin inhibition of PDGF-stimulated vascular smooth muscle cell function: potential mechanism for inhibition of intimal hyperplasia after balloon angioplasty. Am. J. Pathol. *163*, 869–878.

Noseda, M., Harada, M., McSweeney, S., Leja, T., Belian, E., Stuckey, D.J., Abreu Paiva, M.S., Habib, J., Macaulay, I., de Smith, A.J., et al. (2015). PDGFRalpha demarcates the cardiogenic clonogenic Sca1+ stem/progenitor cell in adult murine myocardium. Nat. Commun. *6*, 6930.

Passman, J.N., Dong, X.R., Wu, S.P., Maguire, C.T., Hogan, K.A., Bautch, V.L., and Majesky, M.W. (2008). A sonic hedgehog signaling domain in the arterial adventitia supports resident Sca1^{+} smooth muscle progenitor cells. Proc. Natl. Acad. Sci. USA *105*, 9349–9354.

Pleva, L., Kusnierova, P., Plevova, P., Zapletalova, J., Karpisek, M., Faldynova, L., Kovarova, P., and Kukla, P. (2015). Increased levels of MMP-3, MMP-9 and MPO represent predictors of in-stent restenosis, while increased levels of ADMA, LCAT, ApoE and ApoD predict bare metal stent patency. Biomed. Pap. Med. Fac. Univ. Palacky Olomouc Czech Repub *159*, 586–594.

Psaltis, P.J., Puranik, A.S., Spoon, D.B., Chue, C.D., Hoffman, S.J., Witt, T.A., Delacroix, S., Kleppe, L.S., Mueske, C.S., Pan, S., et al.

(2014). Characterization of a resident population of adventitial macrophage progenitor cells in postnatal vasculature. Circ. Res. *115*, 364–375.

Seidelmann, S.B., Lighthouse, J.K., and Greif, D.M. (2014). Development and pathologies of the arterial wall. Cell Mol. Life Sci. *71*, 1977–1999.

Seo, M., Lee, W.H., and Suk, K. (2010). Identification of novel cell migration-promoting genes by a functional genetic screen. FASEB J. *24*, 464–478.

Simpson, K.J., Selfors, L.M., Bui, J., Reynolds, A., Leake, D., Khvorova, A., and Brugge, J.S. (2008). Identification of genes that regulate epithelial cell migration using an siRNA screening approach. Nat. Cell Biol *10*, 1027–1038.

Singla, S., Hu, C., Mizeracki, A., and Mehta, J.L. (2011). Decorin in atherosclerosis. Ther. Adv. Cardiovasc. Dis. *5*, 305–314.

Sirker, A.A., Astroulakis, Z.M., and Hill, J.M. (2009). Vascular progenitor cells and translational research: the role of endothelial and smooth muscle progenitor cells in endogenous arterial remodelling in the adult. Clin. Sci. (Lond.) *116*, 283–299.

Tang, Z., Wang, A., Yuan, F., Yan, Z., Liu, B., Chu, J.S., Helms, J.A., and Li, S. (2012). Differentiation of multipotent vascular stem cells contributes to vascular diseases. Nat. Commun. *3*, 875.

Tavian, M., Zheng, B., Oberlin, E., Crisan, M., Sun, B., Huard, J., and Peault, B. (2005). The vascular wall as a source of stem cells. Ann. N. Y. Acad. Sci. *1044*, 41–50.

Tigges, U., Komatsu, M., and Stallcup, W.B. (2013). Adventitial pericyte progenitor/mesenchymal stem cells participate in the restenotic response to arterial injury. J. Vasc. Res. *50*, 134–144.

Tilki, D., Hohn, H.P., Ergun, B., Rafii, S., and Ergun, S. (2009). Emerging biology of vascular wall progenitor cells in health and disease. Trends Mol. Med. *15*, 501–509.

Torsney, E., Hu, Y., and Xu, Q. (2005). Adventitial progenitor cells contribute to arteriosclerosis. Trends Cardiovasc. Med. *15*, 64–68.

Tronc, F., Mallat, Z., Lehoux, S., Wassef, M., Esposito, B., and Tedgui, A. (2000). Role of matrix metalloproteinases in blood flow-induced arterial enlargement: interaction with NO. Arterioscler Thromb. Vasc. Biol. *20*, E120–E126.

Tsai, T.N., Kirton, J.P., Campagnolo, P., Zhang, L., Xiao, Q., Zhang, Z., Wang, W., Hu, Y., and Xu, Q. (2012). Contribution of stem cells to neointimal formation of decellularized vessel grafts in a novel mouse model. Am. J. Pathol. *181*, 362–373.

Ueda, K., Yoshimura, K., Yamashita, O., Harada, T., Morikage, N., and Hamano, K. (2015). Possible dual role of decorin in abdominal aortic aneurysm. PLoS One *10*, e0120689.

van Rooij, E., Sutherland, L.B., Thatcher, J.E., DiMaio, J.M., Naseem, R.H., Marshall, W.S., Hill, J.A., and Olson, E.N. (2008). Dysregulation of microRNAs after myocardial infarction reveals a role of miR-29 in cardiac fibrosis. Proc. Natl. Acad. Sci. USA *105*, 13027–13032.

von Marschall, Z., and Fisher, L.W. (2010). Decorin is processed by three isoforms of bone morphogenetic protein-1 (BMP1). Biochem. Biophys. Res. Commun. *391*, 1374–1378.

Williams, K.J., and Tabas, I. (1995). The response-to-retention hypothesis of early atherogenesis. Arterioscler Thromb. Vasc. Biol. *15*, 551–561.

Wong, M.M., Winkler, B., Karamariti, E., Wang, X., Yu, B., Simpson, R., Chen, T., Margariti, A., and Xu, Q. (2013). Sirolimus stimulates vascular stem/progenitor cell migration and differentiation into smooth muscle cells via epidermal growth factor receptor/extracellular signal-regulated kinase/beta-catenin signaling pathway. Arterioscler Thromb. Vasc. Biol. *33*, 2397–2406.

Wu, S.M., Chien, K.R., and Mummery, C. (2008). Origins and fates of cardiovascular progenitor cells. Cell *132*, 537–543.

Xu, Q., Zhang, Z., Davison, F., and Hu, Y. (2003). Circulating progenitor cells regenerate endothelium of vein graft atherosclerosis, which is diminished in ApoE-deficient mice. Circ. Res. *93*, e76–86.

Xu, H., Chen, M., and Forrester, J.V. (2009). Para-inflammation in the aging retina. Prog. Retin. Eye Res. *28*, 348–368.

Xu, X., Zhang, A., Li, N., Li, P.L., and Zhang, F. (2015). Concentration-dependent diversification effects of free cholesterol loading on macrophage viability and polarization. Cell Physiol. Biochem. *37*, 419–431.

Yatera, Y., Shibata, K., Furuno, Y., Sabanai, K., Morisada, N., Nakata, S., Morishita, T., Toyohira, Y., Wang, K.Y., Tanimoto, A., et al. (2010). Severe dyslipidaemia, atherosclerosis, and sudden cardiac death in mice lacking all NO synthases fed a high-fat diet. Cardiovasc. Res. *87*, 675–682.

Yu, B., Wong, M.M., Potter, C.M., Simpson, R.M., Karamariti, E., Zhang, Z., Zeng, L., Warren, D., Hu, Y., Wang, W., et al. (2016). Vascular stem/progenitor cell migration induced by smooth muscle cell-derived chemokine (C-C motif) ligand 2 and chemokine (C-X-C motif) ligand 1 contributes to neointima formation. Stem Cells *34*, 2368–2380.

Zengin, E., Chalajour, F., Gehling, U.M., Ito, W.D., Treede, H., Lauke, H., Weil, J., Reichenspurner, H., Kilic, N., and Ergun, S. (2006). Vascular wall resident progenitor cells: a source for postnatal vasculogenesis. Development *133*, 1543–1551.

Stem Cell Reports, Volume *9*

Supplemental Information

Adventitial SCA-1^{+} Progenitor Cell Gene Sequencing Reveals the Mechanisms of Cell Migration in Response to Hyperlipidemia

Ioannis Kokkinopoulos, Mei Mei Wong, Claire M.F. Potter, Yao Xie, Baoqi Yu, Derek T. Warren, Witold N. Nowak, Alexandra Le Bras, Zhichao Ni, Chao Zhou, Xiongzhong Ruan, Eirini Karamariti, Yanhua Hu, Li Zhang, and Qingbo Xu

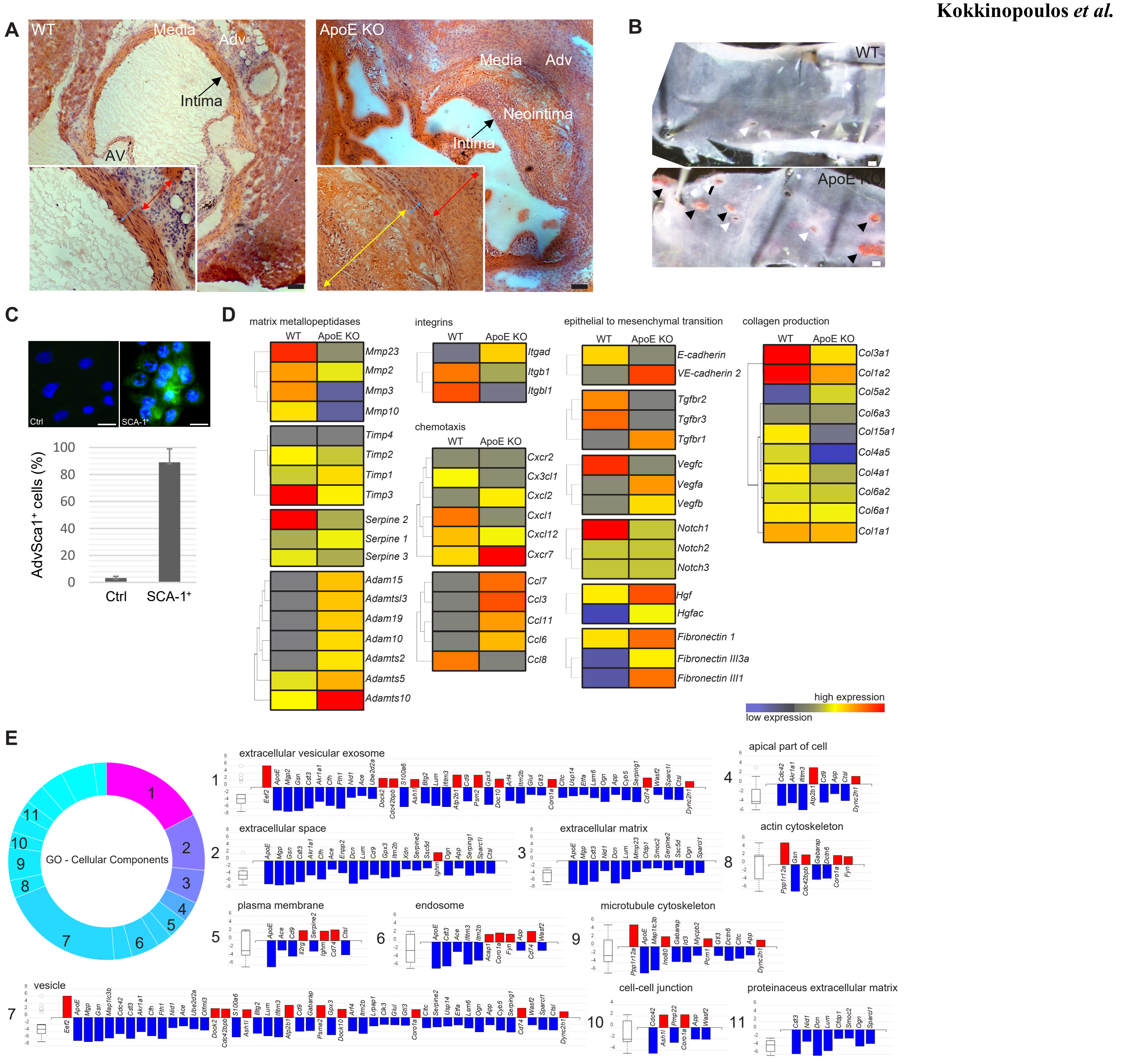

F

bone marrow HSC genes	*Ptprc (Cd45)*	*Thy-1 (Cd90)*	*Cd38*	*Cd34*	*C-kit*	*Cd105*
**WT**	N/A	N/A	N/A	**5%**	N/A	N/A
**ApoE KO**	**10%**	**5%**	N/A	**20%**	N/A	N/A

Figure S1. Single-cell gene expression profile indicates an altered response to extracellular components of adventitial ApoE KO SCA-1^{+} in comparison to WT AdvSCA-1^{+} cells

(**A**) H&E cross-sections of WT and ApoE KO aortic roots, indicating the significant morphological alteration caused in the ApoE KO 6-month old mice, with the formation of neointimal areas (black arrow), and the overall thickening of both media (yellow arrow) and adventitia (red arrow), in comparison to the WT.

(**B**) Flattened thoracic aortic tissues stained with Oil Red, indicating the lipid lesions predominantly appearing in the ApoE KO (black arrowheads).

(**C**) Immunostaining for SCA-1^{+} cells isolated with micro-beads. Aortic adventitial cells from ApoE KO mice were dissociated with collagenase and isolated with micro-beads coupled with an anti-SCA-1 antibody. The isolated cells were labelled with normal rat Ig (Ctrl) or anti-SCA-1 antibody, visualised with anti-rat Ig-conjugated with FITC and counterstained for nuclear visualisation. Means SEM of three experiments.

(**D**) Matrix metallopeptidases and integrins, involved in cell migration, showed an altered gene expression profile in the ApoE KO AdvSCA-1^{+} cell population, in comparison to the WT. Genes involved in chemotaxis were differentially expressed in the ApoE KO, while genes involved in Epithelial to mesenchymal transition (EpMT), showed that ApoE KO AdvSCA-1^{+} had a genotypic profile closer to a more mesenchymal than an epithelial cell, in comparison to WT. Collagen production was showed an altered gene expression pattern, predominantly affecting *Collagens I, III, IV and V*.

(**E**) GO cellular components revealed that genes involved in cell membrane responses to the extracellular environment were generally downregulated in the ApoE KO AdvSCA-1^{+} cell population, in comparison to the WT (Erim pruning, >5 differentially expressed genes). This indicated that the former would behave differently in the presence of different stimuli(s) from the microenvironment, than the latter.

(**F**) Haematopoietic stem cell (HSC) genes were expressed in subpopulations of AdvSCA-1^{+} cells, demonstrating a heterogeneous population.

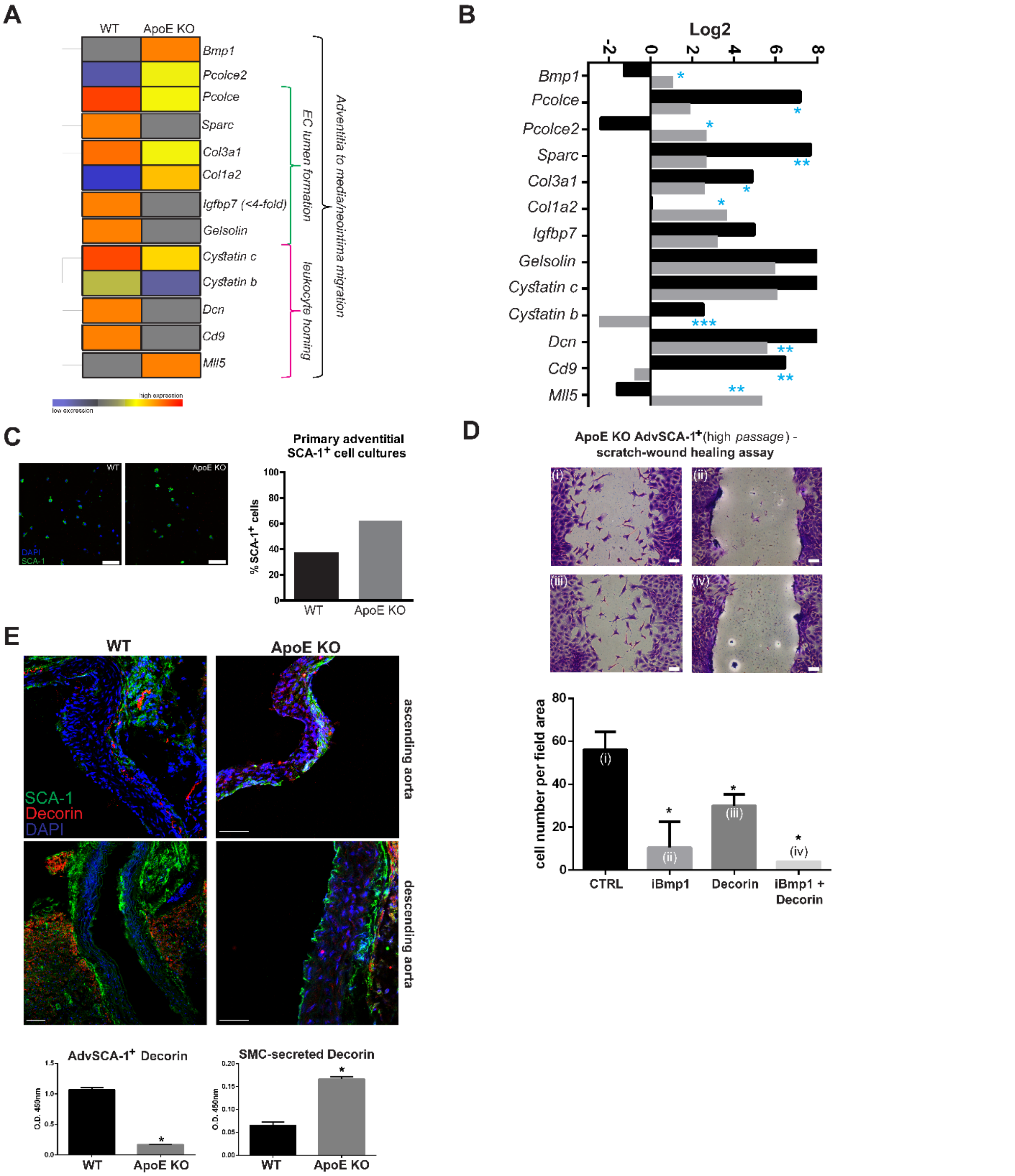

Figure S2. Potential targets for AdvSCA-1$^+$ migration intervention

(**A,B**) Single cell differential gene expression statistical analysis revealed potential targets for Endothelial Cell (EC) lumen formation, leukocyte homing and migration. Black bars represent WT, grey bars ApoE KO.

(**C**) Confirmation of increased SCA-1$^+$ expression in primary ApoE KO adventitia primary cells, in comparison to WT.

(**D**) Wound-healing assays of ApoE KO AdvSCA-1$^+$ cells showed that the use of and inhibitor of BMP1 (iBMP1) with or without DCN, nearly abolished migration, with no apparent cell loss, after 24 hours in culture. DCN administration also showed a statistical decrease in cell migration.

(**E**) DCN expression is downregulated in ApoE KO AdvSCA-1$^+$ 6-month old mice in comparison to age-matched WT animals. SCA-1 (green), DCN (red) and DAPI (blue). Sandwich ELISA indicated the decreased presence of secreted DCN *in vitro*, while in ApoE KO smooth muscle cells (SMCs), DCN was over-secreted, in comparison to WT SMCs.

Graphs are shown as mean ± SEM of three independent experiments. One Way ANOVA with Whitman test. *$*p<0.1$, $**p<0.05$ $***p<0.01$.*

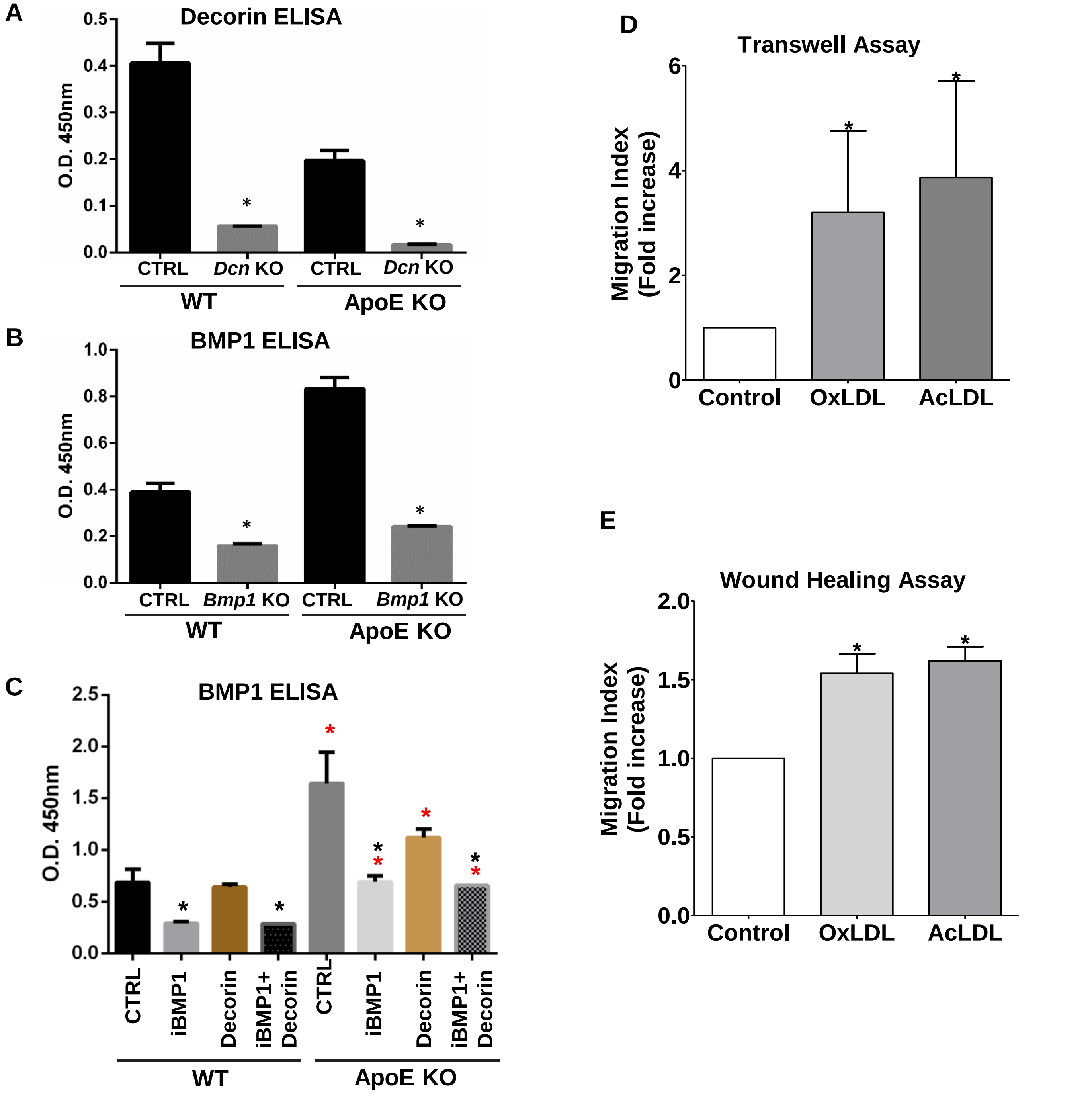

<u>Figure S3</u>. CRISPR KO and BMP1 ELISA assays. Oxidised-LDL and Acetylated-LDL can also induce SCA-1$^+$ cell migration.

(**A,B**) 100,000 cells WT or ApoE KO AdvSCA-1$^+$ *Dcn* and *Bmp1* KO were seeded on petri dishes. Supernatant were collected after 24 hours and ELISA assays were performed. **p<0.05, CTRLs* compared to *CRISPR KO*.

(**C**) 30,000 WT or ApoE KO AdvSCA-1$^+$ cells were seeded onto 12 wells plates and treated with iBMP1 and/or DCN. Supernatant were collected after 24 hours. An ELISA assay was performed against BMP1. Graphs are shown as mean ± SEM of three independent experiments. One Way ANOVA with Whitman test. **p<0.1*, compared with untreated control. **p<0.05* compared *WT* to *ApoE KO*.

(**D**) ApoE KO AdvSCA-1$^+$ were treated either with 5µg/ml Ox-LDL or 20µg/ml Ac-LDL in medium containing 0.2% FBS for 48 hours prior to migration assays. Untreated cells were used as a control. Chemotaxis of vascular progenitor cells in 8.0 µm transwells was documented following 1% crystal violet staining.

(**E**) Migration of vascular progenitor cells was evaluated using a wound-healing assay. Migration index for both assays were defined as the mean number of progenitor cells counted per 5 random fields of view at 20x.

Graphs are shown as mean ± SEM of three independent experiments. **p<0.05* compared with untreated control.

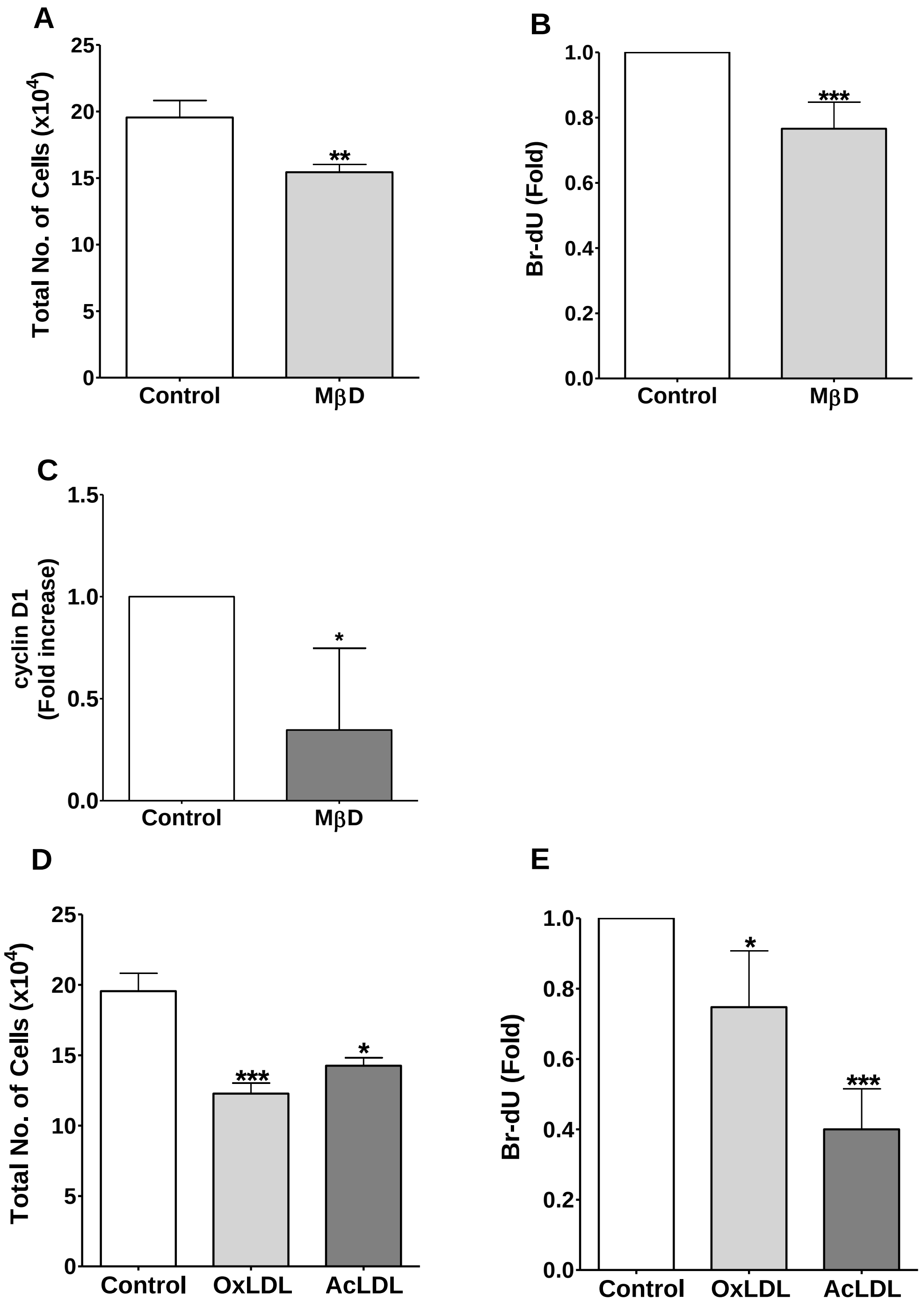

<u>Figure S4</u>. Cholesterol, oxidised-LDL and acetylated-LDL suppresses ApoE KO AdvSCA-1^{+} progenitor cell proliferation Evaluation of cell proliferation in response to 48 hours of chol-MβD loading, 5µg/ml Ox-LDL or 20µg/ml Ac-LDL was confirmed using (**A, D**) quantification of total cell number, (**B, E**) a BrdU incorporation assay and (**C**) detection of cyclin D1 mRNA expression using real time PCR. Graphs are shown as mean ± SEM of three independent experiments. **$p<0.1$, **$p<0.05$, ***$p<0.01$* compared with untreated control.

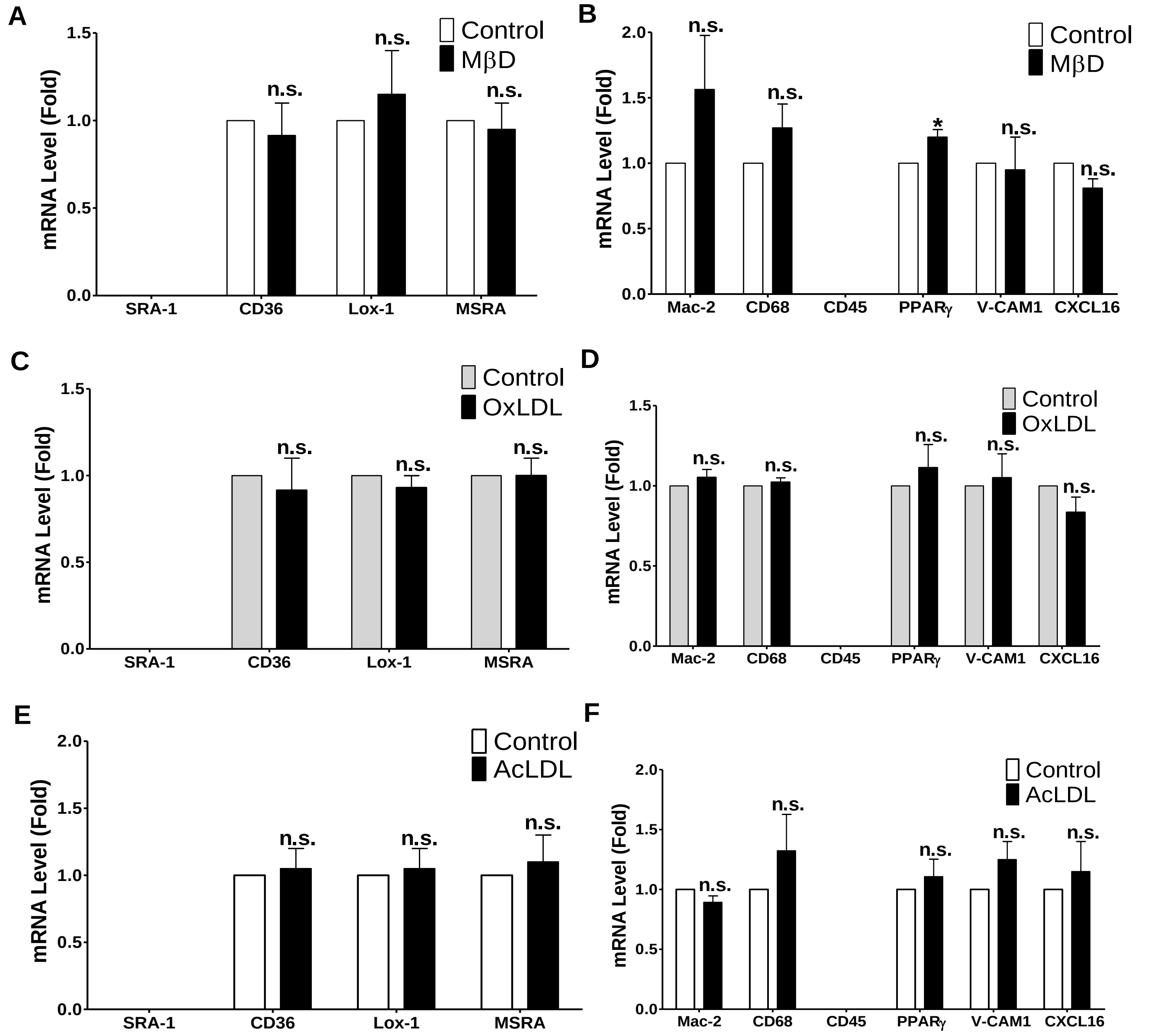

Figure S5. Neither cholesterol, Ox-LDL, or Ac-LDL induce ApoE KO AdvSCA-1$^+$ progenitor cell foam cell-differentiation or scavenger receptor gene-expression changes

(**A, B**) SCA-1$^+$ progenitor cells were loaded with 20μg/ml chol-MβD for 48 hours and cell lysates were subject to real time RT-PCR for detection of foam cell markers at the gene level.

(**C, D**) SCA-1$^+$ progenitor cells were treated for 48 hours with 5μg/ml Ox-LDL and cell lysates were subject to real time RT-PCR for detection of foam cell markers at the gene level.

(**E, F**) SCA-1$^+$ progenitor cells were treated with 20μg/ml Ac-LDL for 48 hours and cell lysates were subject to real time RT-PCR for detection of foam cell markers at the gene level.

SRA-1: steroid receptor RNA activator-1; Lox-1: oxidised low-density lipoprotein receptor-1; MSRA: methionine sulfoxide reductase A; PPAR-γ: peroxisome proliferator-activated receptor-γ; V-CAM1: vascular cell adhesion protein 1; CXCL16: chemokine (C-X-C motif) ligand 16. Graphs are shown as mean ± SEM of three independent experiments. *$p<0.05$ compared with untreated control.

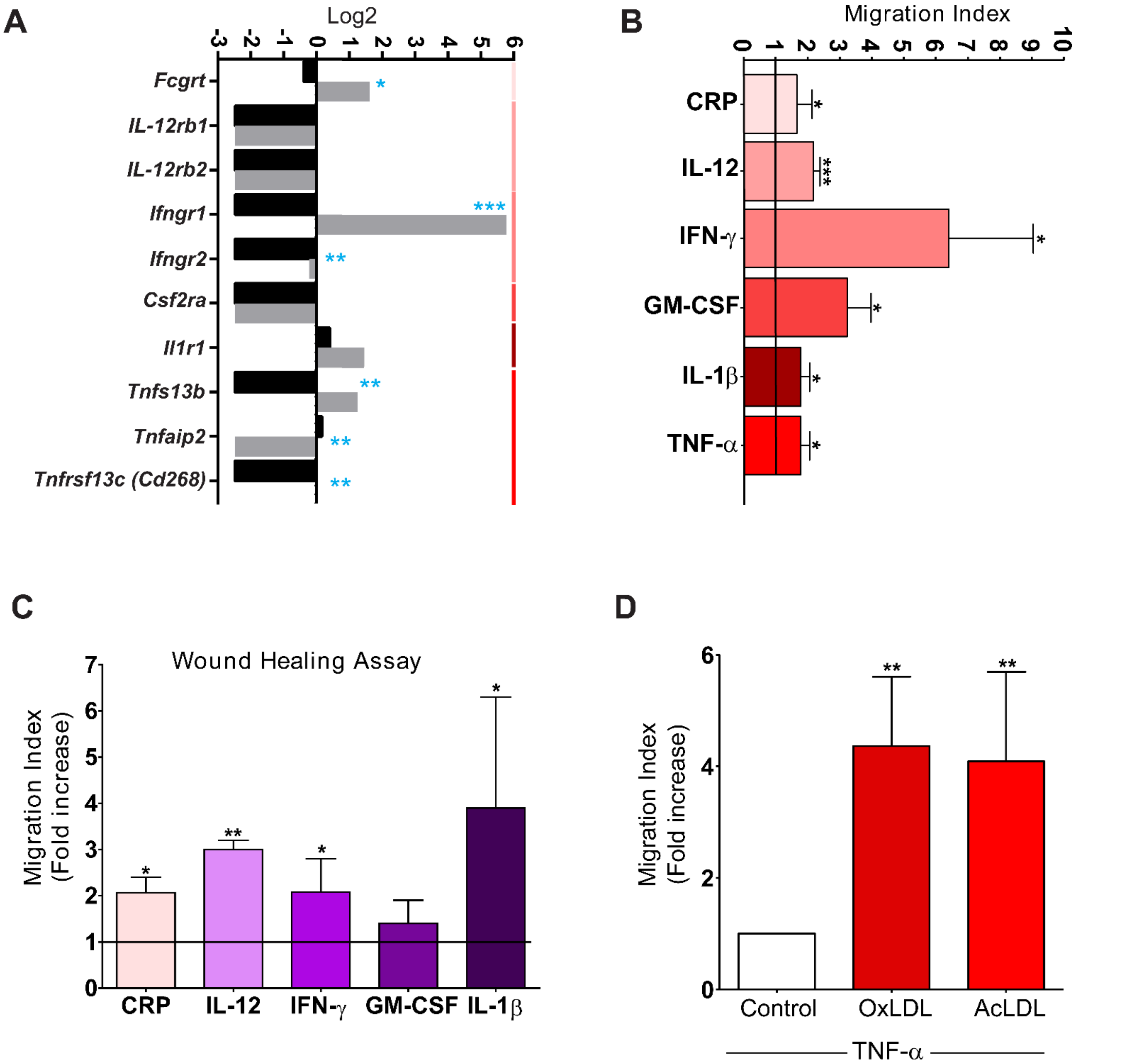

Figure S6. Cholesterol can induce SCA-1+ cell migration towards TNF-α, and a panel of pro-inflammatory cytokines

(**A**) Single-cell gene expression of surface receptors that respond to different cytokines. Black bars represent WT, grey bars ApoE KO.

(**B, C**) Migration of chol-MβD-loaded (or untreated) progenitor cells in response to either CRP (50 ng/ml), IL-12 (10 ng/ml), IFN-γ (50ng/ml), GM-CSF (50ng/ml), IL-1β (10ng/ml) and TNF-α (10ng/ml) was carried out using 8.0µm transwell and wound healing assays.

(**D**) Cells were treated for 48 hours either with 5µg/ml Ox-LDL or 20µg/ml Ac-LDL in medium containing 0.2% FBS prior to migration assays, where they were loaded with TNF-α (10ng/ml). Chemotaxis of vascular progenitor cells in 8.0µm transwells was documented following 1% crystal violet staining.

Migration index for both assays were defined as the mean number of vascular progenitor cells counted per 5 random fields of view at 20x. **p<0.1, **p<0.05, ***p<0.01*.

A

Mature miRNA ID or Gene Symbol	Fold Regulation
mmu-miR-29b-3p	1.9053
mmu-miR-30a-5p	1.5052
mmu-miR-130a-3p	1.4241
mmu-miR-27b-3p	1.5052
mmu-miR-125b-5p	1.5157
mmu-miR-32-5p	2.1287
mmu-miR-28a-5p	1.7654
mmu-miR-10a-5p	1.8277
mmu-miR-10b-5p	-1.4845
mmu-miR-99a-5p	1.6133
mmu-miR-488-3p	-1.3947
mmu-miR-141-3p	3.387

B

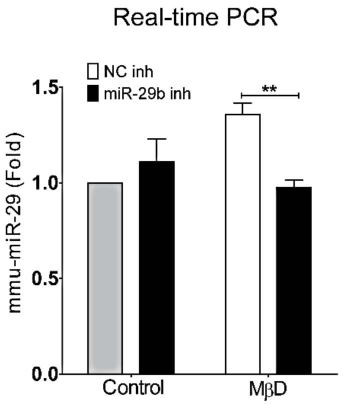

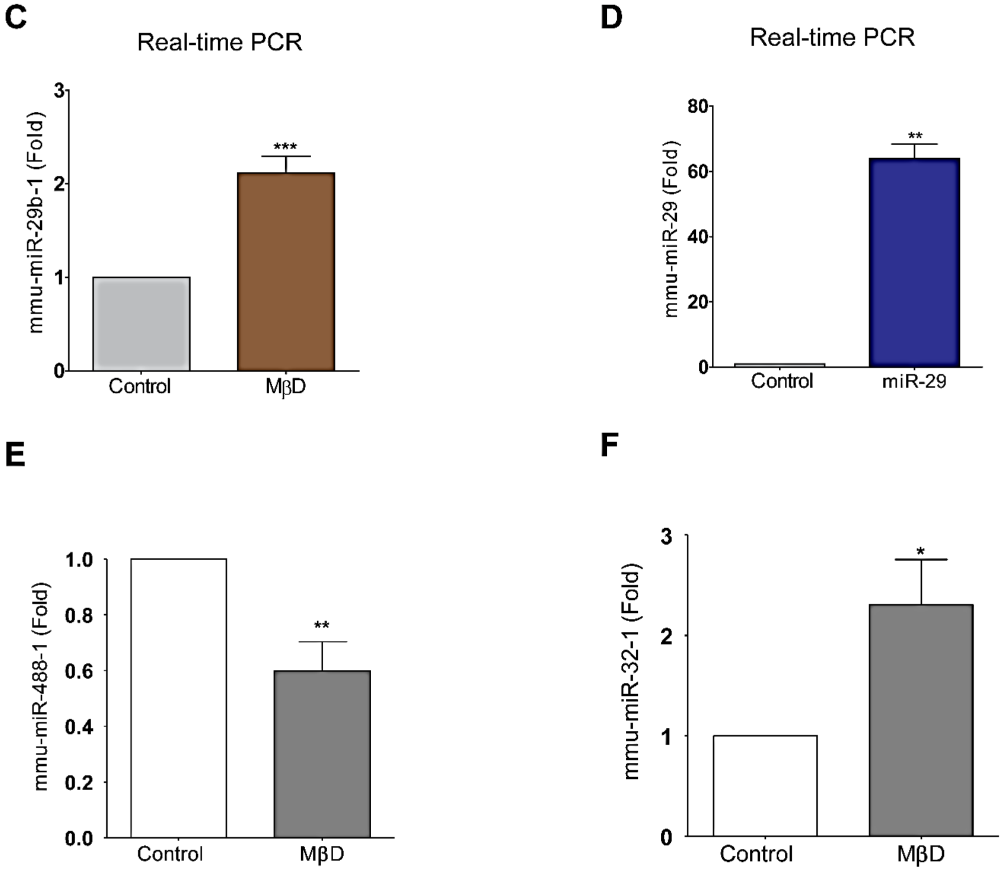

<u>**Figure S7**</u>. Cholesterol can induce SCA-1$^+$ progenitor cell migration via *miRNA-29b-1* upregulation

(**A**) SCA-1$^+$ vascular progenitor cells loaded with 20µg/ml chol-MβD for 48 hours were harvested and subjected to a miScript miRNA PCR Array Mouse miFinder. The miRNAs that showed a statistically significant difference have been highlighted.

(**B**) The induction of miRNA-29b-1 expression in progenitor cells following chol-MβD loading was confirmed using real time RT-PCR.

(**C**) miRNA-29 was over-expressed in SCA-1$^+$ progenitor cells after treatment with a mmu-miR-29b-3p mouse mirVana® miRNA mimic and confirmed using real time RT-PCR. A mirVana ™ miRNA Mimic Negative Control #1 was used as a non-targeting control.

(**D**) miRNA-29b expression in chol-MβD loaded cells was inhibited by treatment with a MH10103 mirVana™ miRNA inhibitor or a mirVana™ miRNA inhibitor Negative Control #1.

Progenitor cells were loaded with 20µg/ml chol-MβD and lysates were harvested for detection of either (**E**) miRNA-488-1 or (**F**) miRNA-32-1 expression using real time RT-PCR.

Graphs are shown as mean ± SEM of three independent experiments. $*p<0.1$, $**p<0.05$, $***p<0.01$ compared with untreated control.

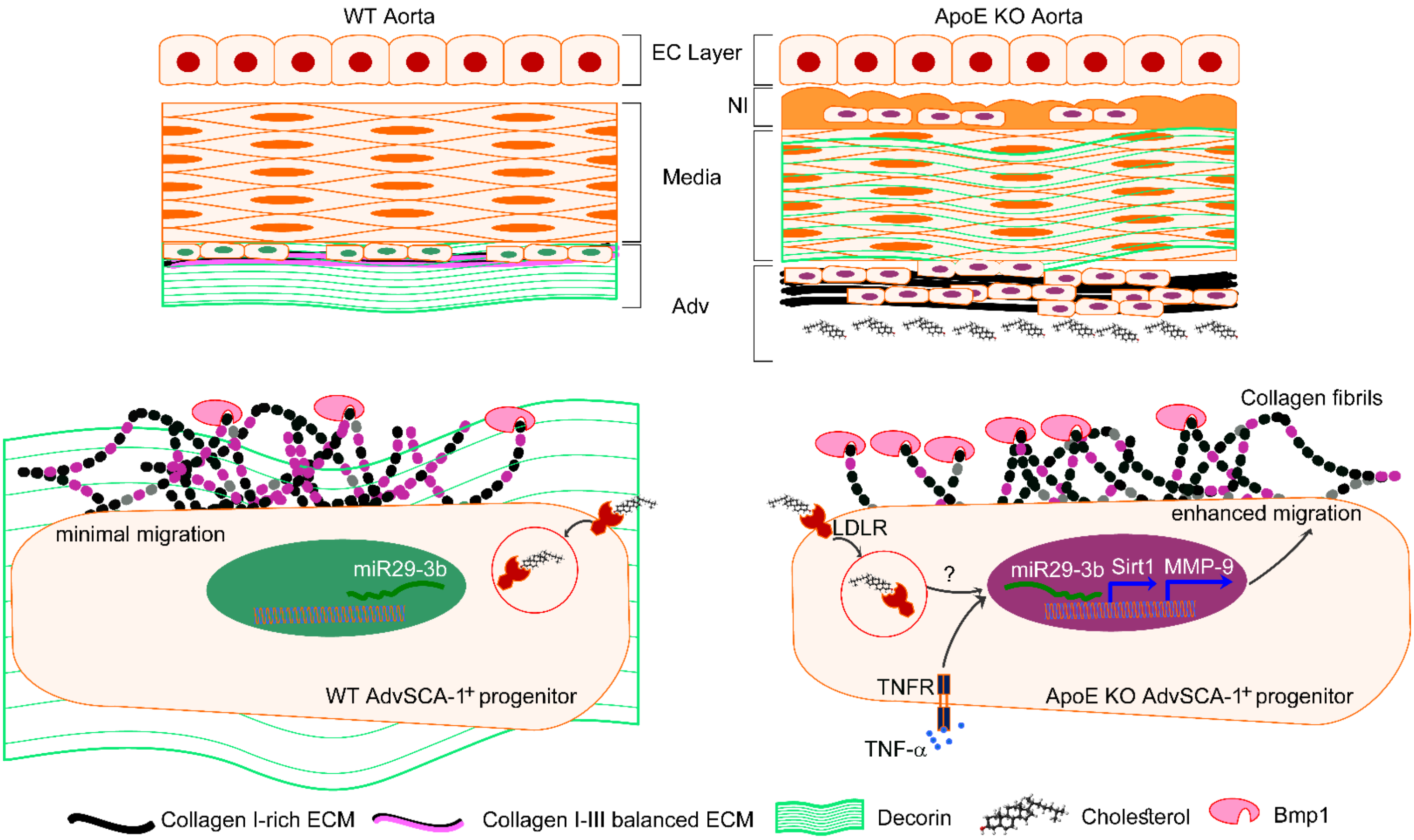

<u>Schematic Diagram S1</u>. Schematic representation of the vascular wall and the intracellular mechanism affecting AdvSCA-1$^+$ migration.

Supplemental Experimental Procedures

Single-cell gene expression analysis

Date from Fluidigm were processed to obtain FASTAQ raw counts. RAW counts were converted to BAM files using Partek Suite, passing QA/QC. For analysis, iPathway software was used for Gene Ontology and DNASTAR Lasergene software for genome alignment (*Mus musculus* mm9) and differential gene expression, using Student's T-test and FDR.

Vascular Progenitor Cell Culture and Differentiation

Tissue grafts were harvested with dexterity from vessel graft samples under a dissection microscope and explanted. Adventitial SCA-1^{+} cells were obtained as described earlier. The purity of isolated AdvSCA-1^{+} cells was confirmed using flow cytometry as shown previously (Hu et al., 2004; Xiao et al., 2007). The SCA-1^{+} progenitor cells were cultured on 2% gelatin-coated flasks in complete stem cell medium (American Type Culture Collection, Rockville, Massachusetts, USA) containing 10% FBS, leukaemia inhibitory factor (10 ng/ml), 0.1mM 2-mercaptoethanol, penicillin (100 U/ml) and streptomycin (100 mg/ml). Smooth muscle differentiation of vascular progenitor cells was performed by culturing the cells on mouse collagen IV (5µg/ml)-coated plates in differentiation medium (DMEM supplemented with 10% FBS, 2mM L-glutamine, 100mg/l gentamicin and 0.5 mM 2-mercapotoethanol) for 5 days. Endothelial differentiation was carried out by culturing the cells on mouse collagen IV (5µg/ml)-coated plates in differentiation medium in the presence of VEGF (10ng/ml) with medium change every 2 days for 10 days. Differentiation was evaluated using real-time RT-PCR to detect the expression of either smooth muscle cell (SMC) markers such as SM-22α, Calponin, and SM-MHCII, or EC markers such as CD31, CD144 and Flk-1.

Oil Red O and H and E Staining

Aortas were obtained from WT and ApoE KO mice aged between 6 and 9 months for staining with Oil Red O. Mouse hearts and aortic roots were obtained from 6 month-old WT and ApoE KO mice and snap frozen in liquid. The tissues were mounted in OCT and sliced into 8 µm thick sections which were mounted on slides and stained with haematoxylin and eosin.

Real-time RT-PCR for RNA detection

Total RNA was isolated from vascular progenitor cells using an RNeasy Mini kit (QIAGEN Inc.) according to manufacturer's instructions. In brief, 2 µg RNA were reverse-transcribed into cDNA with random primers by MMLV reverse transcriptase (RT) (Promega) and real time RT-PCR was performed using 2ng of cDNA per sample with a SYBR Green Master Mix in a 25-µl reaction. Ct values were measured using ABI PRISM 7000 Sequence Detector (Applied Biosystems) and GAPDH RNA was used as an endogenous control to normalize the amounts of RNA in each sample. Sequences of primer sets used in this study are as follows;
Sirt1:5'>GTAAGCGGCTTGAGGGTAAT<3' 5'>GTTACTGCCACAGGAACTAGAG<3',
Lox-1:5'>CAGATGTTAGCCCAGCAGAA<3' 5'>CTCCTCCTGCTCTTTGGATTT<3',
Msra:5'>GTAACAGCCAAACACCATGTC<3' 5'>GAAGCAGCCCATTCCAAATAC<3',
Mac-2:5'>AGGAGAGGGAATGATGTTGCC <3' 5'>GGTTTGCCACTCTCAAAGGG<3',
Cd68:5'>TTGGGAACTACACACGTGGGC<3' 5'>CGGATTTGAATTTGGGCTTG<3',
Cd45:5'>CAGAGCATTCCACGGGTATT<3' 5'>GGACCCTGCATCTCCATTTAT<3',
Ppar-y:5'>GAACCTGCATCTCCACCTTATT<3' 5'>TGGAAGCCTGATGCTTTATCC<3',
Cxcl16:5'>CCCTTGTCTCTTGCGTTCTT<3' 5'>TCTGGGTGCCAGAAGAAATG<3',
Cyclin D1:5'> AGACCTGTGCGCCCTCCGTA<3' 5'>GGCCAGCGGGAAGACCTCCT<3'.
Sequences of other SMC markers 28, 29, EC markers5, CD366, SRA-16 and V-CAM16 primer sets used were as previously described both by our laboratory and others, respectively. *Mmp-9* mRNA detection was based on the use of fully validated primer sequences designed and purchased from Primer Design, UK.

Immunocytochemistry

Progenitor cells were seeded in gelatin-coated chamber slides (BD Biosciences) prior to loading with 20µg/ml chol-MβD or treatment with 5µg/ml Ox-LDL and 20µg/ml Ac-LDL. Cells cultured in the absence of cholesterol or the modified LDL were used as controls. After a 48 hour incubation, the cells were fixed with 4% paraformaldehyde, permeabilized with 0.1% Triton X-100 in PBS and blocked with 10% normal swine serum (Dako). Incubation of cells with phalloidin (either Alexa Fluor® 488 or Alexa Fluor® 546) was performed at 4°C overnight, followed by at least 3 thorough washes with PBS of 5 mins each time. Cells were counterstained with DAPI (1:1000 in PBS) for 3mins at room temperature and mounted with fluorescent mounting medium (Dako) before image acquisition using an Axio Imager.M2 microscope and AxioVision Digital Imaging System (Carl Zeiss Ltd.).

Immunohistochemistry (*en face* and cryosections)

Aortas were fixed by perfusion with 4% PFA before permeabilisation with 0.2% Triton X-100. Primary antibodies were (VE-Cadherin Santa-Cruz, #sc-6458, 1:250 and SCA-1 Abcam, #ab51317, 1:100, DCN Abcam, # ab137508, 1:100) and incubations were carried out overnight at 4°C. Secondary antibody (AlexaFluor 488 and AlexaFluor 594 1:500) incubations were for 1 hour at room temperature. The stained arch and 5 mm thick aortic rings were separated and opened on to slides before mounting in a hard set mounting medium. When dry slides were viewed using a Leica SP5 Confocal Microscope. Additionally, aortas were isolated from WT and ApoE KO mice, dissected laterally and the intima and medial layer peeled away allowing for the staining of the adventitia as previously described.

Enzyme-linked immunosorbent assays (ELISA) assays

ELISAs were employed to compare the level of proteins both in cell extracts and supernatants between WT and ApoE KO AdvSCA-1^+ cells, according to the manufacturers' protocols; Decorin (Abcam, #ab207618), Procollagen I alpha 1 (Abcam, #ab210579), procollagen II (MyBiosource, #MBS2000208), collagen II (MyBiosource, #MBS720538) and BMP1 (MyBiosource, #MBS2019285). All assays were sandwich ELISAs, following the manufacturers' instructions.

Exogenous Proteins, Antagonists, Cholesterol and Modified Low-Density Lipoprotein (LDL) Loading

Cholesterol (chol-MβD) was obtained from Sigma-Aldrich, whilst human copper (Cu++) oxidised (Ox-LDL) and acetylated (Ac-LDL) low-density lipoproteins were obtained from Cell Biolabs Inc. and Molecular Probes, respectively. Murine recombinant proteins of pro-inflammatory cytokines TNF-α, IL-12, IFN-Y, GM-CSF, and IL-1β were purchased from Peprotech UK. Mouse recombinant C-reactive protein (CRP) was obtained from R&D Systems. Inhibitor EX-527 (C13H13ClN2O) was purchased from Santa Cruz Biotechnology and MMP-9 Inhibitor II (C16H17F2N3O3S) from Merck Millipore. The inhibitors were used at concentrations of 50µM and 20µM, respectively.

Cholesterol was loaded into SCA-1^+ cells using a Chol-MβD complex obtained as "water-soluble cholesterol" that contained ≈50 mg of cholesterol/g solid (molar ratio, 1:6 cholesterol/MβD). Chol-MβD was reconstituted in dH20 and kept in the fridge for up to 7 days. Progenitor cells were incubated with Chol-MβD (20 µg/ml) for 48 hours in DMEM, alpha-MEM containing only 0.2% FBS. Progenitor cells were also incubated with either Ox-LDL (5µg/ml) or Ac-LDL 20 µg/ml) for 48 hours in medium containing 0.2% FBS. Cells incubated for 48 hours in medium (containing 0.2% FBS) without Chol-MβD, Ox-LDL or Ac-LDL served as controls for all experiments.

Western Blot Analysis

Harvested progenitor cells were lysed with IP-A buffer (25 mM Tris-HCl pH 7.5, 150 mM NaCl, 1 mM EDTA pH 8.0, 1% Triton X-100 plus protease inhibitors) and proteins were sequentially measured using the Bradford method. 30 µg of lysate was applied to SDS-PAGE before being transferred to a nitrocellulose membrane (Amersham Biosciences), followed by a standard western blotting procedure. Polyclonal antibody against MMP-9 was purchased from Santa Cruz Biotechnology, Inc. and used to detect the respective protein. The bound primary antibody was detected using an HRP-conjugated secondary antibody and an ECL detection system (Amersham Biosciences).

CRISPR/Cas9 genome editing

Dcn and *Bmp1* gene sequences were disrupted using Genescript's *Dcn* and *Bmp1* CRISPR guide RNA in the pSpCas9 BB-2A-Puro (PX459) v2.0 plasmid along with the creation of supercoiled plasmids for optimal delivery and cellular expression. For plasmid delivery (2.4 µg per cell type) to both $5x10^5$ WT and ApoE KO AdvSCA-1^+ cells, an 4D Amaxa nucleofector™ core unit and the reagents provided were used (program FI-115, P3 solution), following the manufacturer's protocol. Plasmid uptake and expression were tested using a constitutively expressed GFP construct (data not shown). Transduced cells were treated with 4 µg puromycin (Gibco) after plating for 48 hours. Viable cells were *passaged* >4 times to excluded possible non-integrated plasmid puromycin resistance cassette expression along with consecutive rounds of puromycin treatment. WT and ApoE KO AdvSCA-1^+ *Dcn* and *Bmp1* KO cells were tested for protein expression using ELISA (for DCN and BMP1) on supernatants, in order to confirm the knockdown of both proteins.

Transwell Chemotaxis & Scratch-wound Assays

Migration assays were carried out using transwell inserts with 8.0 micron pore membrane filters (Becton Dickinson Labware, USA). Progenitor cells were incubated for 48 hours in medium containing only 0.2% FBS in the absence or presence of either chol-MβD, Ox-LDL or Ac-LDL. Cells were harvested using trypsin-EDTA and subsequently loaded onto the upper chamber at $5x10^4$ cells/ml of serum free medium. The bottom chamber contained serum-free medium with either TNF-α (10ng/ml) or other pro-inflammatory cytokines (i.e. IL-12 (10ng/ml), IFN-γ (50ng/ml), GM-CSF (50ng/ml), CRP (50ng/ml) and IL-1β (10ng/ml)). After an overnight incubation, non-migrating cells on the upper side of filters were washed with care using PBS and removed using cotton tip applicators. Vascular progenitors on the underside of the membrane were fixed with 4% PFA for 10 mins before staining with 1% crystal violet solution (diluted with dH20) at room temperature for at least 10 mins. Data was expressed as the mean number of migrated vascular progenitor cells in 5 random fields of view (at 20x). For experiments

that involved inhibitors (i.e. EX-527, MMP-9 inhibitor), progenitor cells were pre-treated with the respective chemicals for an hour before loading onto transwells. Serum free medium in the bottom chamber also contained the respective antagonists/inhibitors.

Progenitor cells were seeded in a 12-well plate at $4x10^5$ cells per well in complete culture medium. Upon reaching confluency, the cells were incubated for 48 hours in medium containing 0.2% FBS in the absence or presence of either chol-MβD, Ox-LDL or Ac-LDL. The next day, a straight scratch was made using a 1ml pipette tip to generate a 'wound'. The pipette tip was kept at an angle of around 30 degrees during the scratch to ensure that scratch widths were consistent in every well. The wells were gently washed twice with PBS to remove all cellular debris as a result of the scratch prior to treatment with either TNF-α (10ng/ml) or other pro-inflammatory cytokines (IL-12 (10ng/ml), IFN-γ (50ng/ml), GM-CSF (50ng/ml), CRP (50ng/ml) and IL-1β (10ng/ml). After an overnight incubation, the migration of vascular progenitor cells into the 'wound' area was documented using a phase contrast microscope and subsequently quantified. Data was expressed as the mean number of migrated vascular progenitor cells in 5 random fields of view of the 'wound' (at 20x). For experiments that involved inhibitors (i.e. EX-527, MMP-9 inhibitor or DMSO as vehicle control), the vascular progenitor cells were pre-treated with the respective chemicals for an hour before performing the scratch. Medium that was used during the migration also contained the respective antagonists/inhibitors.

BrdU Cell Proliferation Assay

Upon reaching confluence, $SCA-1^+$ progenitors were cultured overnight in the absence (serum free medium only) or presence of either chol-MβD, Ox-LDL or Ac-LDL. Progenitor cell proliferation was quantified using a Cell Proliferation ELISA Assay, BrdU (colorimetric) (Roche) according to manufacturer's instructions. Cells were incubated in BrdU labelling solution (10µM) for 2 hours at 37°C before adding 200µl of FixDenat solution into each well for 30 mins at room temperature. The solution was removed thoroughly prior to incubation with BrdU conjugated antibody for 90 mins. After 3 washes with PBS, 100µl of substrate solution was added and incubated at room temperature until a change in colour was detected. An amount of 25µl 1M H2SO4 was immediately added to stop the reaction before measuring the absorbance at 450nm (correction at 690nm).

Single Cell Migration and Tracking using Time-Lapse Microscopy

ApoE KO $AdvSCA-1^+$ progenitor cells were seeded at $5x10^3$ cells per well of a 6-well plate and allowed to adhere to the bottom of the wells. Subsequently, the cells were loaded either with chol-MβD, Ox-LDL or Ac-LDL in the presence of medium containing 0.2% FBS for 48 hours. The plate was then placed into a microscope chamber of a fully motorised, multi-field time-lapse microscope (Eclipse TE 2000-E; Nikon) with a charge-coupled device camera (ORCA; Hamamatsu Photonics), and maintained at 37˚C and 5% CO2. Images were acquired from 10 random fields per well, every 5 minutes for 20 hours, using a 4x objective with bright field settings. Images were acquired using a Volocity software (PerkinElmer) and 10 random single cells in each field were tracked using the ImageJ manual tracking plugin (National Institute of Health). Analysis of migrational speed and persistence was performed using Mathematica 6.0 (Wolfram Research Ltd, USA) custom-written notebooks kindly provided from Professor Graham Dunn & Daniel Soong, King's College London.

Micro-RNA Extraction, Reverse Transcription and Real-Time RT-PCR

Extraction of total RNA including miRNA was carried out using a miRNeasy mini kit, according to the manufacturer's protocol. Briefly, 700µl of QIAzol Lysis Reagent was added to each sample and incubated for 5mins at room temperature. Subsequently, 140µl of chloroform was added to the tubes, shaken vigorously and incubated at room temperature for 2-3mins before centrifugation for 15mins at 12,000xg at 4˚C. The upper aqueous phase was mixed with 1.5 volumes of 100% ethanol and transferred into an RNeasy® Mini column before further centrifugation at ≥8000xg for 15s. A total of 700µl Buffer RWT was added followed by 500µl Buffer RPE twice; samples were centrifuged ≥8000xg and flow through was discarded between every step. In a new collection tube, 40µl RNase-free water was added to the mini columns and centrifuged for 1min at ≥8000xg to elute RNA.

Micro-RNA reverse transcription and amplification of 1µg of sample were performed using a miScript® II RT Kit with miScript HiSpec Buffer as described by the manufacturer. Briefly, template RNA was added to tubes containing a master mix (5x miScript HiSpec Buffer, 10x miScript Nucleics Mix, RNase-free water and miScript Reverse Transcriptase Mix). The tubes were incubated for 60mins at 37˚C, followed by 95˚C for 5mins.

A total of 1ng of cDNA (per well) was subjected to a miScript miRNA PCR Array Mouse miFinder (cat. no. MIMM 001Z) that was used in combination with a miScript SYBR Green PCR Kit to assess the expression of a panel of miRNAs and carried out according to the manufacturer's protocol. Individual miRNA of mmu-miR-29b-3p, mmu-miR-488-3p and mmu-miR-32-5p were confirmed using respective miScript miRNA PCR Arrays, also in combination with miScript SYBR Green PCR Kits. In brief, template cDNA was dispensed into individual wells of a 96-well PCR plate containing a reaction mix (2x QuantiTect SYBR Green PCR Master Mix, 10x miScript Universal Primer, 10x miScript Universal Assay and RNase-free water) before performing the cycler program (15mins at 95˚C and 15s at 94˚C, 30s at 55˚C, 30s at 70˚C for 40 cycles) on a

qPCR machine (Eppendorf Mastercycler® ep Realplex2, Eppendorf UK). All reagents were otherwise purchased from Qiagen.

Micro-RNA Transient Transfection

An mmu-miR-488-3p mouse mirVana® miRNA mimic (5µM), a mmu-miR-32-5p mouse mirVana® miRNA mimic (5µM), a mmu-miR-29b-3p mouse mirVana® miRNA mimic (5µM), or a non-targeting control (mirVana™ miRNA Mimic Negative Control #1, 5µM) (Ambion Life Technologies). Inhibition of miR-29b was performed using a hsa-miR-29b-3p ID: MH10103 mirVana™ miRNA inhibitor (60nM) or a mirVana™ miRNA inhibitor Negative Control #1 (60nM) (Ambion Life Technologies). All transfections were performed on SCA-1^+ progenitor cells seeded at $2x10^5$ cells/well of 6-well plates using Lipofectamine™ RNAiMAX (Invitrogen), according to the manufacturer's protocol.

Quantification of Mean Cell Size

ApoE KO AdvSCA-1^+ progenitor cells were seeded in a $T75cm^2$ flask at $5x10^5$ cells and cultured complete medium. The next day, the cells were cultured overnight in the absence (serum free medium only) or presence of either chol-MβD (cholesterol), Ox-LDL or Ac-LDL. The cells were then trypsinized and subjected to cell size calculation using a Multisizer 3 Coulter Counter (Beckman Coulter) according to the manufacturer's instructions.

Statistical Analysis

All data in this study were presented as the mean and standard error of the mean (SEM) of at least three separate experiments. The analysis was performed using GraphPad Prism V.4 (GraphPad Software, San Diego CA) using statistical analysis with analysis of variance (ANOVA) followed by Dunnett’s multiple comparison tests, or Student’s T-Test. Significance was considered when $p<0.05$. For Figures 3 and 4, a 2-way ANOVA was performed for comparison between similar time points or treatments against the untreated control. For Figure 4B, a Student’s one-tailed T-Test was performed.

Supplemental References

Hu, Y., Zhang, Z., Torsney, E., Afzal, A.R., Davison, F., Metzler, B., and Xu, Q. (2004). Abundant progenitor cells in the adventitia contribute to atherosclerosis of vein grafts in ApoE-deficient mice. J Clin Invest *113*, 1258-1265.

Xiao, Q., Zeng, L., Zhang, Z., Hu, Y., and Xu, Q. (2007). Stem cell-derived SCA-1+ progenitors differentiate into smooth muscle cells, which is mediated by collagen IV-integrin alpha1/beta1/alphav and PDGF receptor pathways. Am J Physiol Cell Physiol *292*, C342-352.
